# CCM signaling complex (CSC) couples both classic and non-classic Progesterone receptor signaling

**DOI:** 10.1186/s12964-022-00926-z

**Published:** 2022-08-15

**Authors:** Johnathan Abou-Fadel, Xiaoting Jiang, Brian Grajeda, Akhil Padarti, Cameron C. Ellis, Esmeralda Flores, Alyssa-Marie D. Cailing-De La O, Jun Zhang

**Affiliations:** 1 Department of Molecular and Translational Medicine (MTM), Texas Tech University Health Science Center El Paso, 5001 El Paso Drive, El Paso, TX 79905 USA; 2 Department of Biological Sciences, University of Texas at El Paso, El Paso, TX 79902 USA

**Keywords:** Cerebral cavernous malformation (CCM), Signaling complex (CSC), Progesterone (PRG), Mifepristone (MIF), Prognostic biomarkers, Classic nuclear progesterone receptors (nPRs), Non-classic membrane progesterone receptors (mPRs/PAQRs), Proteomics, RNAseq

## Abstract

**Background:**

Breast cancer, the most diagnosed cancer, remains the second leading cause of cancer death in the United States, and excessive Progesterone (PRG) or Mifepristone (MIF) exposure may be at an increased risk for developing breast cancer. PRG exerts its cellular responses through signaling cascades involving classic, non-classic, or combined responses by binding to either classic nuclear PRG receptors (nPRs) or non-classic membrane PRG receptors (mPRs). Currently, the intricate balance and switch mechanisms between these two signaling cascades remain elusive. Three genes, *CCM1-3*, form the CCM signaling complex (CSC) which mediates multiple signaling cascades.

**Methods:**

Utilizing molecular, cellular, Omics, and systems biology approaches**,** we analyzed the relationship among the CSC, PRG, and nPRs/mPRs during breast cancer tumorigenesis.

**Results:**

We discovered that the CSC plays an essential role in coupling both classic and non-classic PRG signaling pathways by mediating crosstalk between them, forming the CmPn (CSC-mPRs-PRG-nPRs) signaling network. We found that mPR-specific PRG actions (PRG + MIF) play an essential role in this CmPn network during breast cancer tumorigenesis. Additionally, we have identified 4 categories of candidate biomarkers (9 intrinsic, 2 PRG-inducible, 1 PRG-repressive, 1 mPR-specific PRG-repressive, and 2 mPR-responsive) for Luminal-A breast cancers during tumorigenesis and have confirmed the prognostic application of *RPL13* and *RPL38* as intrinsic biomarkers using a dual validation method.

**Conclusions:**

We have discovered that the CSC plays an essential role in the CmPn signaling network for Luminal-A breast cancers with identification of two intrinsic biomarkers.

**Video Abstract**

**Supplementary Information:**

The online version contains supplementary material available at 10.1186/s12964-022-00926-z.

## Introduction

Breast cancer remains the second leading cause of cancer death in the United States, and excessive Progesterone (PRG) or Mifepristone (MIF) exposure, such as females under Hormone Replacement Therapy (HRT) or taking hormonal contraceptives during their reproductive ages, may be at an increased risk for developing breast cancer. PRG exerts its cellular responses through signaling cascades involving classic, non-classic, or combined responses by binding to either classic nuclear PRG receptors (nPRs) or non-classic membrane PRG receptors (mPRs) [1]. It has been well-defined that PRG commonly binds to nPRs as transcriptional factors defined as classic actions [2]. nPRs have two major isoforms (PR1/2) which are alternative transcripts with different cellular functions [2]. PRG can evoke both genomic and non-genomic actions, defined as membrane-initiated responses, through classic nPRs [3]. Recently, two new groups of membrane-bound PRG receptors, which are unrelated to nPRs, have been identified, including 5 members of the membrane progestin receptors (mPRs)/Class II progestin and adipoQ receptors (PAQRs) and 4 members of the b5-like heme/steroid-binding protein family known as sigma2 receptor (S2R)/PRG receptor membrane components (PGRMC1/2) [4, 5]. It has been shown that major mPRs are highly expressed in reproductive tissues [6] and function by coupling to G-proteins [7] to execute rapid non-genomic actions. Interestingly, homozygous *mPR*s mutant fish were generated but with no phenotype, suggesting redundancy among mPRs [8].

Three genes, *KRIT1* (*CCM1*), *MGC4607* (*CCM2*), and *PDCD10* (*CCM3*), form the CCM signaling complex (CSC) that mediates multiple signaling cascades [9–11]. All three CCM proteins are differentially expressed among various cancerous tissues/cells, in which nearly half of *CCMs* gene expression changes (44%) were found in reproductive tumors [6, 10], suggesting the CSC might play a significant role in reproductive tumorigenesis [6, 10]. Among all the reproductive cancers with altered *CCMs* gene expression, breast tumors seemed to have the most drastic changes [6].

Given the important functions of nPRs/mPRs for PRG signaling, the hypersensitive response of breast cancer tumor cells to hormonal changes, and our recent discovery of the CSC’s differential expression in reproductive tumors [6, 10], we utilized The Cancer Genome Atlas’s (TCGA) database, to evaluate the basal expression of key members of nPRs, mPRs and the CSC among breast cancer tissues. Our results identified significant differences in gene expression across multiple diagnostic parameters, supporting their possible use as diagnostic markers to distinguish between luminal-like breast cancer subtypes. Additionally, we identified significant differences in gene expression among various TNM staging of tumors reflecting the size and extent of the primary tumor (T), lymph node infiltration (N), and metastases (M) mechanisms in Luminal-A breast cancer subtypes. These preliminary results gave us the foundation to start exploring expression levels *in-vitro*, using Luminal-A subtype breast cancer cell line T47D and MCF7, which demonstrated that the CSC is essential for coupling both classic and non-classic PRG receptors by mediating crosstalk between them and forming the novel CSC-mPRs-PRG-nPRs (CmPn) signaling network. Depletion of any of the three *CCMs* genes results in the disruption of either classic or non-classic PRG receptors-mediated signaling. Similarly, silencing any of the classic nPRs or non-classic mPRs/PAQRs leads to disruption of the CmPn signaling network. We further discovered that mPR-specific PRG actions (treatment with PRG and MIF, only targeting mPRs) result in disruption of the CSC, indicating that the CmPn signaling network relies on an intricate feedback system to balance multiple signaling pathways to maintain homeostasis. mPR-specific PRG actions has recently been validated in nPR(-) Triple Negative Breast Cancer cells (TNBC, which only express mPRs) and nPR(-) vascular endothelial cells [12–14]. Additionally, utilizing omic approaches, we have elucidated alterations in signaling mechanisms under mPR-specific PRG actions, or disrupted CSC conditions, affecting numerous essential signaling cascades, further solidifying the essential role of the CmPn network during breast cancer tumorigenesis. Finally, combining our omics approaches using Luminal-A breast cancer cells, under mPR-specific PRG actions, and systems biology analysis utilizing luminal-like clinical breast tumor samples, we identified 14 potential diagnostic biomarkers with significant differential expression between the three luminal-like breast cancer subtypes. Focusing only on Luminal-A and normal-like breast cancer tissues (identical receptors’ status as Luminal-A), we confirmed significant Kaplan–Meier (KM) survival curves for 13/14 diagnostic biomarkers utilizing two cohorts of patient clinical expression data for Luminal-like breast cancers. Finally, we assessed the overlaps between the two Luminal-A breast cancer cohorts (dual validation) among our Luminal-A breast cancer cell omics data validating two intrinsic candidate biomarkers with significant clinical KM survival curves for Luminal-A breast cancers. Together, these results reinforce the potential role of the CmPn signaling network during breast cancer tumorigenesis and demonstrate that disruption of this network could lead to worsened outcomes.

## Methods

### Cell culture, treatments, and performance assays

#### Cell culture and treatments

T47D and MCF7 cells were cultured in RPMI1640 medium following manufacturer’s instructions (ATCC). When cells reached 80% confluency, cells were treated with either vehicle control (ethanol/DMSO, VEH), mifepristone (MIF, 20 µM), progesterone (PRG, 20 µM), estrogen (EST, 10 nM), gradient concentrations of progesterone (PRG, 1–80 µM) and mifepristone (MIF, 5–160 µM), mPR-specific PRG treatment (PRG + MIF; 20 µM each), or media only (Untreated), respectively for steroid treatments. For RNA knockdown experiments, 80% confluent T47D breast cancer cells were transfected with a set of siRNAs, targeting specific genes (Additional file 1: Table S1), by RNAiMAX (Life Technologies) as described before [6, 10].

#### Cell migration assay

Briefly, a cell scratch was pressed through the confluent T47D and MCF7 breast cancer cell monolayers in the plate. The cells were swept away on one side of that line. Vehicle control (EtOH, DMSO), MIF (20 µM), PRG (20 µM), or mPR-specific PRG treatment (MIF + PRG, 20 µM each) were added to start the experiments. Migration was monitored temporally. The migration area was visualized using a Nikon Biostation and recorded with a high-resolution digital camera. The migration area was measured temporally from four different fields under 20 × magnifications for each condition and cell type (n = 3) (more details in Additional file 1).

#### Wound healing assay

T47D and MCF7 cells were cultured in 24-well plates (5 × 10^4^ cells/well) to reach confluency. Cells were starved in FBS-free medium for 3 h, then a 100 µl pipette tip was used to scratch the monolayer to create a middle wound groove. Cells were cultured in triplicates in FBS-free medium in the vehicle control (EtOH, DMSO), MIF (20 µM), PRG (20 µM), or mPR-specific PRG treatment (MIF + PRG, 20 μM each). More details in Additional file 1.

### Immunohistochemistry (IHC) and immunofluorescence (IF)

IF/IHC staining methods were performed as previously described [6]. For detailed methodology and antibody information, please see Additional file 1 and Additional file 1: Table S2.

### RNA extraction, RT-qPCR, and RNAseq for various cell lines

Total RNAs were extracted with TRIZOL reagent (Invitrogen) following the manufacturer’s protocol. For cultured breast cancer cells, monolayer was rinsed with ice cold PBS and then lysed directly in a culture dish by adding 1 ml of TRIZOL reagent per flask and scraping with a cell scraper. The cell lysate was passed several times through a pipette and vortexed thoroughly. The quality (purity and integrity) of each RNA sample was assessed using a Bioanalyzer (Agilent) before downstream applications. All RNA-seq data were produced using Illumina HiSeq 2000; clean reads for all samples were over 99.5%; 60–80% of reads were mapped to reference genomes.

#### Real time quantitative PCR analysis (RT-qPCR)

RT-qPCR assays were designed using primer sets (Additional file 1: Table S3) and applied to quantify the RNA levels of the endogenously expressed *CCMs* (*1/2/3*) and *mPRs* (*PAQR5/6/7/8/9*) using Power SYBR Green Master Mix with ViiA 7 Real-Time PCR System (Applied Biosystems). RT-qPCR plates with nPR( +) breast cancer cell-lines were prepared using an epMotion 5075 automated liquid handling system (Eppendorf). RT-qPCR data were analyzed with DataAssist (ABI) and Rest 2009 software (Qiagen). The relative expression levels (2^−ΔCT^) were calculated from all samples and normalized to β-actin; fold change (2^−ΔΔCT^) comparisons were performed by further normalizing to control groups [15]. All experiments were performed with triplicates.

#### RNA-seq processing of files to assemble interactomes for nPR(+) breast cancer cells

The RNA-seq files were obtained through paired-end (PE) sequencing with 100 bp reads (2X100) in Illumina HiSeq2000. The data consisted of 6 FASTQ files, 2 PE FASTQ files for each of the three groups: T47D_Veh 48 h, T47D_MIF + PRG treated 48 h, and T47D_MIF treated 48 h. All cohorts consisted of two samples and the RNA-seq files were processed and analyzed as previously described [11]. The overlaps were inputted into the PANTHER [16] classification system (GeneONTOLOGY) as well as iDEP [17] (Integrated Differential Expression and Pathway Analysis) program to build signaling networks (details in Additional file 1: Fig. S7 legend).

### Protein extraction, Western blots and proteomics analysis for various cell lines

#### Protein extraction and quality assessment

Cells were harvested and lysed using a digital sonifier (Branson model) in ice cold lysis buffer containing 50 mM Tris–HCl (pH 7.5), 150 mM NaCl, 0.5% NP-40 (Sigma), 50 mM sodium fluoride (Sigma), 1 mM PMSF (Sigma), 1 mM dithiothreitol (Invitrogen) and 1 EDTA-free complete protease inhibitor tablet (Roche). Protein concentration of lysates were measure by Qubit assay (Invitrogen) before proceeding. For proteomics analysis, proteins were prepped using the filter-aided sample preparation (FASP) method following manufacturer’s instructions (Expedeon, San Diego, CA). Finally, samples were digested with trypsin (Sigma-Aldrich, St. Louis, MO) and peptides were eluted using 0.1% formic acid.

#### Western blots (WB)

The relative expression levels of candidate proteins were measured with WB. Equal amount of protein lysates from different treatments and cell lines were loaded into Criterion precast gels for SDS-PAGE gel electrophoresis and transferred onto PVDF membranes at 4 °C, then probed with antibodies (Additional file 1: Table S2) as described before [6, 10, 14, 18].

#### Liquid chromatography-tandem mass spectrometry (LC–MS/MS)

The cell lysates were generated from seven cohorts, T47D_Veh 72 h, T47D_MIF + PRG treated 72 h, T47D_MIF treated 72 h, T47D_scramble 72 h, T47D_CCM1-Knockdown (KD) 72 h, T47D_CCM2-KD 72 h, and T47D_CCM3-KD 72 h. All cohorts consisted of three replicates and were processed and analyzed as previously described [11]. Additional details in Additional file 1: Fig. S8 legend.

#### Proteomics processing of files for T47D breast cancer cells

Proteomic data analysis was performed as previously described [11]. The Human Database was downloaded in FASTA format on May 1, 2020, from UniProtKB; http://www.uniprot.org/; 177,661 entries. Common contaminants such as trypsin autolysis fragments, human keratins, and protein lab standards were included in the contaminants database [19]. Proteomic samples were analyzed via Students *t*-test. A cutoff of *p* ≤ 0.05 was executed to determine significance in the comparisons.

#### Processing of proteomic files to assemble interactomes for T47D breast cancer cells

A Python script was created to identify shared differentially expressed proteins (DEPs) between cohorts. A scoring system of proteins identified in at least 3 groups were used to improve data validation. The overlaps were inputted into the PANTHER [16] classification system (GeneONTOLOGY) as well as iDEP [17] program to build signaling networks.

#### Omics analysis to assemble interactomes for T47D breast cancer cells at both the transcriptional and translational levels

A Python script was created to identify shared differentially expressed genes/proteins between cohorts. The overlaps were inputted into the PANTHER [16] classification system (GeneONTOLOGY) as well as iDEP [17] program to build signaling networks. Identified overlaps were compared between proteomics and RNA-seq data. The agreements in differential expression were noted using the Python comparison script.

### Prognostic effects for identified candidate biomarkers associated with a perturbed CmPn signaling network

#### Assessing metastasis transformation using Epithelial-mesenchymal transition (EMT) score data from 7 pan-breast cancer cohorts

We utilized EMTome software to analyze EMT signature profile data (EMT scores) from 7 publicly available pan-breast cancer cohorts [20–26] for our identified candidate biomarkers. To do this we averaged EMT scores (mean ± SEM) from all 7 databases and graphed them using box and whisker plots to assess the metastasis transformation potential.

#### Analysis of differential expression of our identified candidate biomarkers and key CmPn members using microarray expression data

Differential expression profiling was performed by utilizing the TCGA TARGET GTEx database using two types of 'normal' tissues; (1). “tissue normal” which are taken from normal tissue, near the tumor site and (2). ‘normal tissue’ from individuals without cancer. Data used for this analysis is from the UCSC RNA-seq Compendium, where TCGA, TARGET, and GTEx samples are re-analyzed (re-aligned to human hg38 reference genome and expressions are analyzed using RSEM and Kallisto methods) by the same RNA-seq pipeline which allows all samples to be processed using a uniform bioinformatics pipeline, therefore batch effects are eliminated. Analysis was automatically performed using the Xena platform with default parameters.

Microarray data containing nPR(+) breast cancer tumors were analyzed using kmplotter [27]. The tumor samples were divided into two groups based on nPR status determined by IHC. This resulted in 925 nPR(−) and 926 nPR(+) breast cancer samples of which expression data was obtained for the identified candidate biomarkers in this study. Additionally, we also assessed expression profiles of key CmPn members using the same databases to include phenotypic filters including tissue type, sample type, receptor status, PAM50 classification, as well as other diagnostic parameters [28].

#### Construction of Kaplan–Meier (KM) survival curves for identified candidate biomarkers to determine prognosis effects

Publicly available microarray data (22,277 probes) from 1809 breast cancer patients was analyzed using KMplotter [27] to integrate gene expression and clinical data simultaneously. Additionally, publicly available microarray data was also assessed from TCGA to integrate gene expression and clinical data simultaneously [28] to confirm the initial analysis performed using kmplotter. To ensure the patients in the database reflected cohorts seen in the everyday clinical practice, we filtered the patient data by only selecting cohort data similar to SEER published prevalence’s [27]. Breast cancer patients were additionally filtered to only analyze patient samples classified as ER(+)/nPR(+)/HER2(−)/Luminal-A subtype (indentical to T47D subtype). Logrank *P*-values were calculated by the software [27] as well as hazard ratios and 95% confidence intervals.

### Statistical analysis

For RT-qPCR analysis**,** all pairwise multiple comparison procedures were analyzed using Tukey and Student’s *t*-test. For Western blots, all pairwise multiple comparison procedures were analyzed using Tukey and Student’s *t*-test. For cell migration/invasion and wound healing assays, two-way analysis of variance (ANOVA) was used to detect the differences in the mean temporal values among the treatment groups in migration and wound healing assays. For invasion assays, all pairwise multiple comparison procedures were analyzed using Tukey and Student’s *t*-test. For immunohistochemistry analysis**,** Student’s *t*-test was used to detect the differences in the mean values among the treatment groups. For transcriptomics/proteomics analysis**,** all pairwise multiple comparison procedures were analyzed using Tukey and Student’s *t*-test. For microarray analysis, statistical significance was performed with students *t*-test or one-way ANOVA (depending on comparing groups). All graphs/plots/charts were constructed and produced by SigmaPlot 12.0 (Systat Software, Inc.), GraphPad Prism 8 and the Xena platform.

## Results

### Altered expression of *nPRs*, *mPRs* and *CCM* genes across clinical tumor data suggests their involvement in breast cancer tumorigenesis

**Fig. 1:**
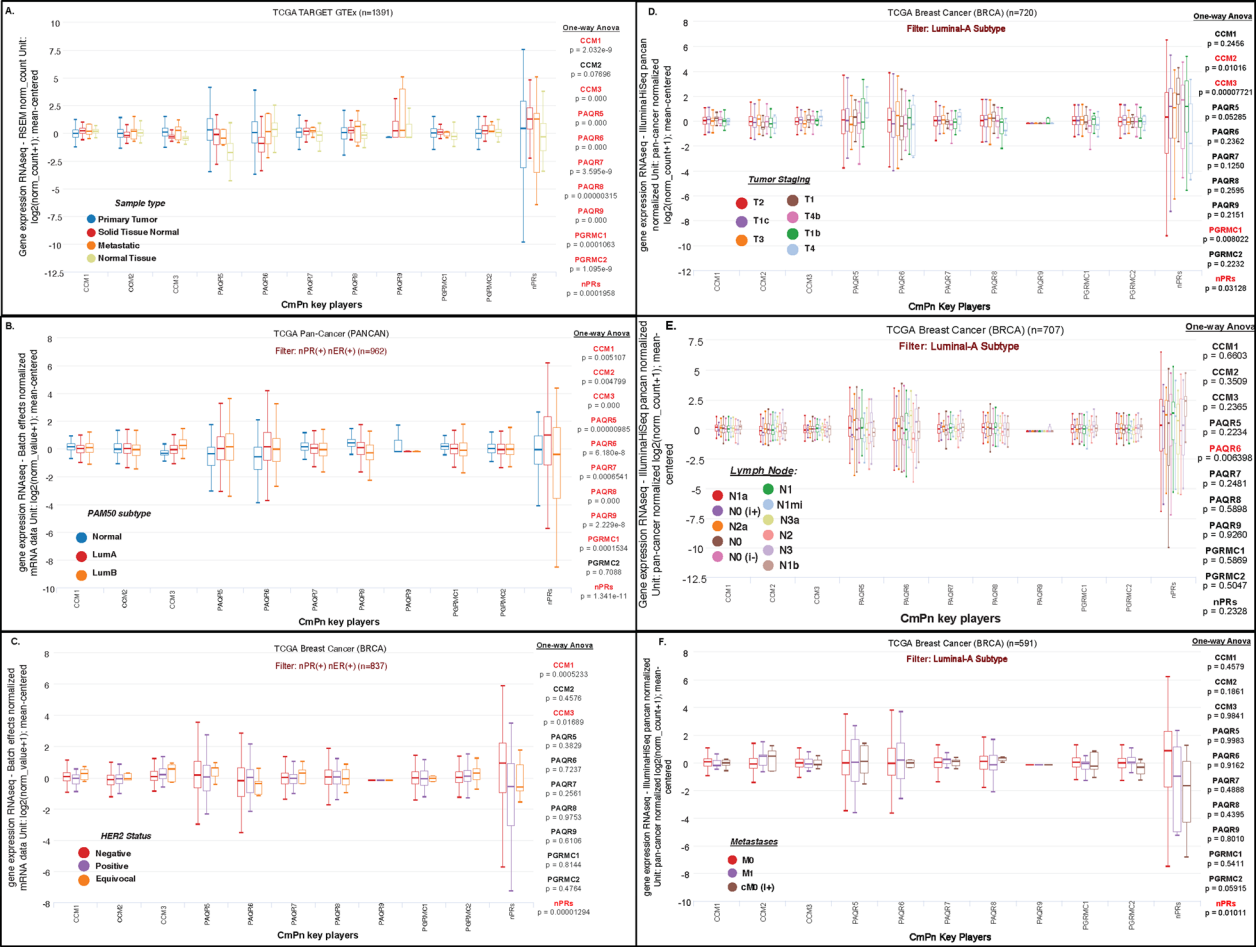
RNAseq expression profiling for key CSC/mPR/nPR players utilizing The Cancer Genome Atlas (TCGA) breast cancer database: We investigated key CSC, mPRs and nPR players expression analysis using both types of 'normal' tissues; “tissue normal” which are taken from normal tissue, near the tumor site, and “normal tissue” from individuals without cancer. **A** *CmPn expression profiles based on tissue type.* **B** *CmPn expression profiles between Luminal-type cancers*. **C** *CmPn expression profiles, based on HER2 status, between Luminal-type breast cancers.* **D** *CmPn expression profiles based on clinical tumor (T) staging in Luminal-A breast tumors.* **E** *CmPn expression profiles based on lymph node (N) staging in Luminal-A breast tumors.* **F** *CmPn expression profiles based on metastases (M) staging in Luminal-A breast tumors.* For all graphs, X axis details genes profiled, while Y axis details Log2 RSEM normalized RNAseq expression data (panel **A**), Log2 batch-effect normalized RNAseq expression data (panels **B**–**C**) or Log2 PANCAN normalized (panels **D**–**F**) RNAseq expression data (mean-centered). All graphs were produced using the Xena platform. Significance was performed automatically by the Xena platform using One-way ANOVA and default parameters. For legend details for panels **D**–**F**, see end of Additional file 1: Fig S10 legend

Given our recent findings on the potential role of the CSC during reproductive tumorigenesis, and the important functions of nPRs/mPRs for PRG signaling [6, 10], we evaluated the basal expression of key players of the CSC (*CCM1-3*), mPRs (*PAQR5-9, PGRMC1/PGRMC2*) and nPRs (*PR1/2*) in breast cancer tumor tissues using publicly available databases including The Cancer Genome Atlas (TCGA). We first assessed expression profiles using the TCGA-TARGET-GTEX database which allows for analysis in not only metastatic tumor tissues but also “solid tissue normal” which are taken from normal tissue, near the tumor site, as well as containing expression data from ‘normal tissue’ from individuals without cancer. In this analysis, we observed significant differences in expression of almost all 11 genes profiled (except for *CCM2*) suggesting their involvement during breast cancer tumorigenesis (Fig. 1A). We next sought to repeat our analysis, this time utilizing the TCGA PANCAN database, which allows for expression analysis by filtering tissues based on PAM50 classification, allowing us to filter breast cancer tissues for only luminal-like subtypes. We included in this analysis Luminal-A, normal-like tissue (identical receptors’ status as Luminal-A), and Luminal-B (also similar to Luminal-A, but with flexibility in HER2 receptor status, and high levels of Ki-67). We observed significant differences in expression of almost all 11 genes profiled (except *PGRMC2*) between the three sub-types of luminal-like breast cancers (Fig. 1B) further suggesting their involvement during breast cancer tumorigenesis, and identifying for the first time, their diagnostic potential in distinguishing between luminal-like breast cancers. Based on this, we again repeat our analysis, but this time focusing on HER2 receptor status, to determine if there are any connections between CmPn members’ expression levels and corresponding HER2 receptor status. Interestingly our analysis demonstrated significant expression differences for only *CCM1, CCM3*, and *nPRs* (Fig. 1C) in luminal-type breast cancers, based on HER2 expression, suggesting a novel relationship between expression levels for these three genes and HER2 expression. Together, these results reinforce the potential involvement of the CmPn signaling network during breast cancer tumorigenesis and provide further validation for their potential use as diagnostic biomarkers to distinguish between Luminal-type breast cancers. Given these results, we next assessed whether altered expression of these genes was associated with a worst prognosis in Luminal-A breast cancers.

### Differential expression patterns of *nPRs*, *mPRs* and *CCM* genes across Luminal-A breast tumors and their associated prognostic effects

The tumor size (T), regional lymph node involvement (N), and metastases of the primary tumor (M), defined as TNM staging, is a well-recognized standard in prognostic cancer staging. Since the TCGA database contains phenotypic filters that allow for filtering of tissues, based on TNM staging, we evaluated the basal expression of the CSC, nPRs, and mPRs for each category, using only Luminal-A breast cancer tissue subtypes. Interestingly, our analysis revealed significant alterations in the expression of *CCM2, CCM3, PGRMC1,* and *nPRs* across various tumor-staging categories (Fig. 1D). These results suggest the involvement of the CmPn signaling network in influencing the size and extent of the primary tumor in Luminal-A subtypes. Next, we repeated expression profiling to evaluate genes potentially involved in infiltration mechanisms into the lymph nodes. We discovered significant differences in the expression of *PAQR6* across various tumor lymph node status clinical samples, suggesting this key mPR may be potentially involved in lymph node infiltration mechanisms (Fig. 1E). Finally, we assessed expression profiles to evaluate genes potentially involved in metastases mechanisms. Our analysis revealed significant changes in gene expression of *nPRs* across various stages of tumor metastases, suggesting the potential involvement of *nPRs* in Luminal-A breast cancer metastases.

**Fig. 2:**
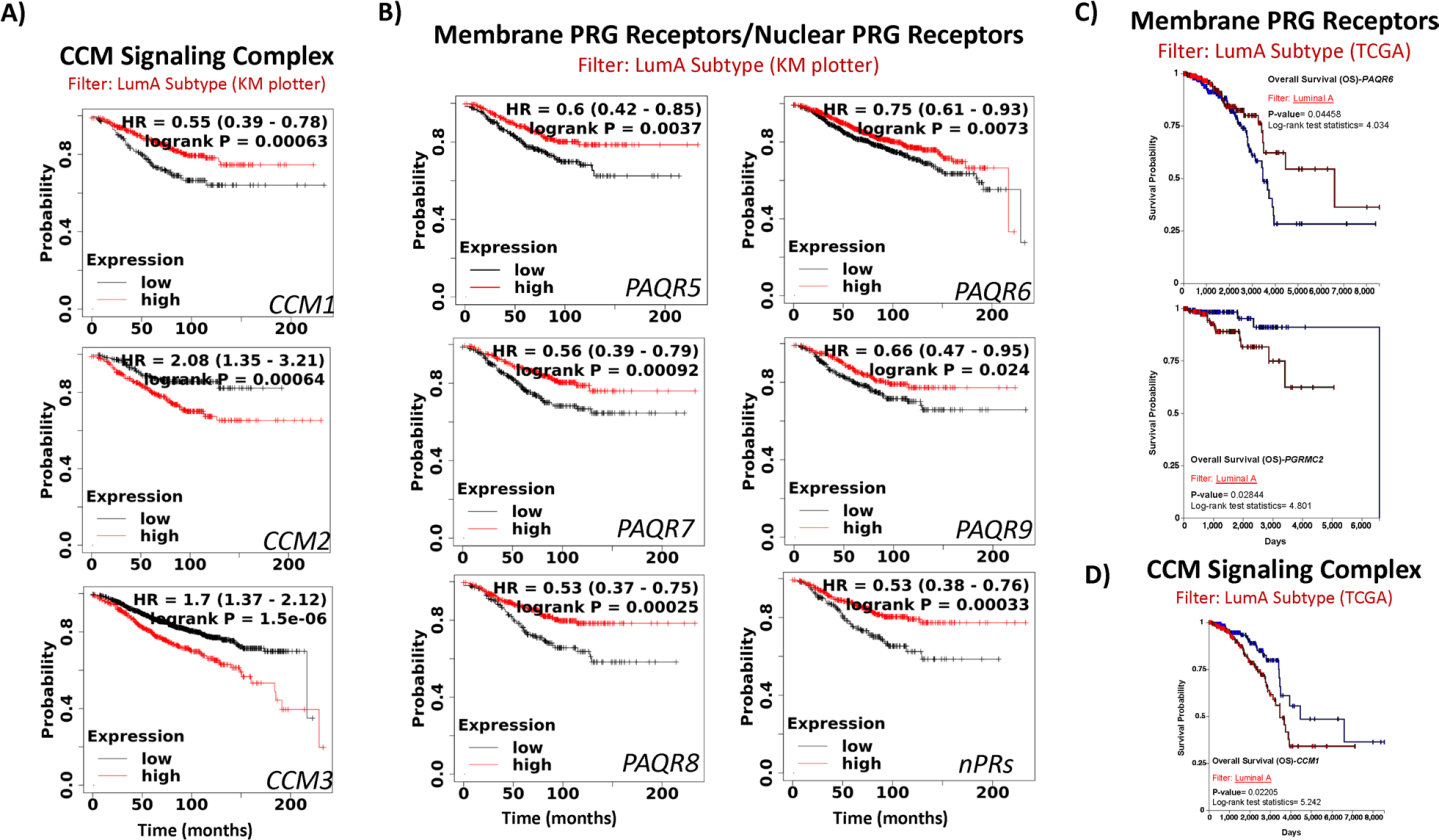
Prognostic effects for key CmPn players utilizing microarray data of breast cancer patients. Publicly available microarray data from breast cancer patients was analyzed using either kmplotter or Xena browser (TCGA database) to integrate gene expression and clinical data simultaneously to generate the displayed Kaplan–Meier (KM) survival curves. **A** Prognostic effects [Overall survival (OS)] for *CSC expression in Luminal-A type breast cancers using kmplotter.* **B** OS for *mPRs/nPRs expression in Luminal-A type breast cancers using kmplotter.* **C** OS for *mPRs expression in Luminal-A type breast cancers using TCGA.* **D** OS for *CSC expression in Luminal-A type breast cancers using TCGA.* Logrank *P*-values are calculated and displayed as well as hazard ratio (and 95% confidence intervals) for panels **A**–**B** automatically calculated by the software using default parameters. Logrank *P*-values are calculated and displayed as well as logrank test statistics for panels **C**–**D** automatically calculated by the software using default parameters. Red line demonstrates high gene expression (all panels), while black line (panels **A**–**B**) or blue line (panels **C**–**D**) demonstrates low gene expression

To further evaluate prognostic effects for altered CSC, nPRs, and mPRs expression in Luminal-A subtype breast cancer tissues, we utilized publicly-available breast cancer tumor tissue gene expression data (microarray) [27] integrating gene expression and clinical data simultaneously to generate Kaplan–Meier (KM) survival curves for all members of the CmPn network. First, breast cancer tumor data were filtered to only analyze patient samples classified as Luminal-A subtypes (ER(+)/nPR(+)/HER2(−)). When assessing significant expression differences for members of the CmPn network, our analysis revealed that decreased expression of *CCM1*, while increased expression of *CCM2/CCM3* had worst prognostic effects in Luminal-A breast cancers (Fig. 2A), re-affirming our previous notion of the essential role of the CSC during breast cancer tumorigenesis. Our analysis also revealed that decreased expression of all 5 major mPRs (*PAQR5-9*), as well as *nPRs,* had worst prognostic effects in Luminal-A breast cancers (Fig. 2B), providing support for the essential roles of both mPRs/nPRs during Luminal-A breast cancer tumorigenesis. Interestingly, when we repeated our analysis utilizing a different breast cancer database (TCGA), we confirmed that decreased expression of *PAQR6* had the worst overall survival (OS) (Fig. 2C, top panel) for Luminal-A breast cancer patients, validating our previous results using a separate cohort (Fig. 2B). Additionally, this new cohort demonstrated that increased expression of *PGRMC2* also resulted in decreased OS rates in Luminal-A breast cancers that were not previously identified (Fig. 2C). Finally, increased expression of *CCM1* was confirmed to significantly decrease OS (Fig. 2D) in Luminal-A breast cancers. Together, these results further support the potential involvement of the CSC, nPRs, and mPRs signaling during breast cancer tumorigenesis, and elucidate the great potential of assessing expression data for the CSC, nPRs, and mPRs for prognostic applications in Luminal-A breast cancers.

### Upregulation of CCM proteins validate their potential involvement in breast cancer tumorigenesis

#### Significantly increased expression levels of CCM proteins in breast cancer tissues

To validate our TCGA expression profiling and our recent findings that CCM2 expression is frequently perturbed in breast tumors [6, 10], we further investigated additional breast cancer tissue pairs using different panels with larger sample sizes. Increased CCM2 expression was observed in breast tumor tissues compared to normal tissues (Additional file 1: Fig. S1A, left panels), with statistical significance from the entire collection (Additional file 1: Fig. S1A, right panel). We next examined co-expression levels of CCM1/3 proteins in identified human breast cancers using immunofluorescence (IF) imaging. The coordinated increase of both CCM1/3 proteins was observed in breast tumors compared to normal tissues (Additional file 1: Fig. S1B, left panel) with statistical significance from the entire collection (Additional file 1: Fig. S1B, right panel). Western blots further validated our IF imaging data (Additional file 1: Fig. S1C). Assessment of mPRs expression demonstrated increased PAQR7 expression in breast tumor tissue compared to normal tissue (Additional file 1: Fig. S1D, left and middle panels), with statistical significance from the entire collection (Additional file 1: Fig. S1D, right panel), supporting previous observations of increased *PAQR7* RNA expression in breast cancers [29]. Overall, expression levels of all three CCM proteins are upregulated in breast tumors compared to normal tissues, in concordance with increased expression of PAQR7, suggesting a coordinated relationship between the CSC and mPRs during breast cancer tumorigenesis [6].

#### Highly expressed CCM proteins in Luminal-A breast cancer cell lines

To elucidate mechanisms of the CSC’s involvement in breast cancer tumorigenesis and based on our initial TCGA expression profiling (Fig. 1), we screened candidate tumor cell lines and identified Luminal-A breast cancer cell line, T47D, as having higher endogenous expression, and a richer composition, of *CCM2* isoforms (Additional file 1: Fig. S2), which were recently defined [10]. As a well-differentiated breast cancer cell line, derived from an invasive ductal carcinoma, T47D is categorized as ER-positive(+), nPR(+), and HER2-negative(−) [30]. Additionally, nPRs in T47D cells, with equally expressed isoforms (PR1/2), are estrogen insensitive [31], but markedly susceptible to PRG [32], making it an ideal model for dissecting the CSC, nPRs, and mPRs signaling networks, under mPR-specific PRG actions.

### *CSC couples both classic and non-classic PRG signaling in nPR(* +*) T47D cells*

#### PRG and MIF work synergistically to disrupt the CSC

**Fig. 3:**
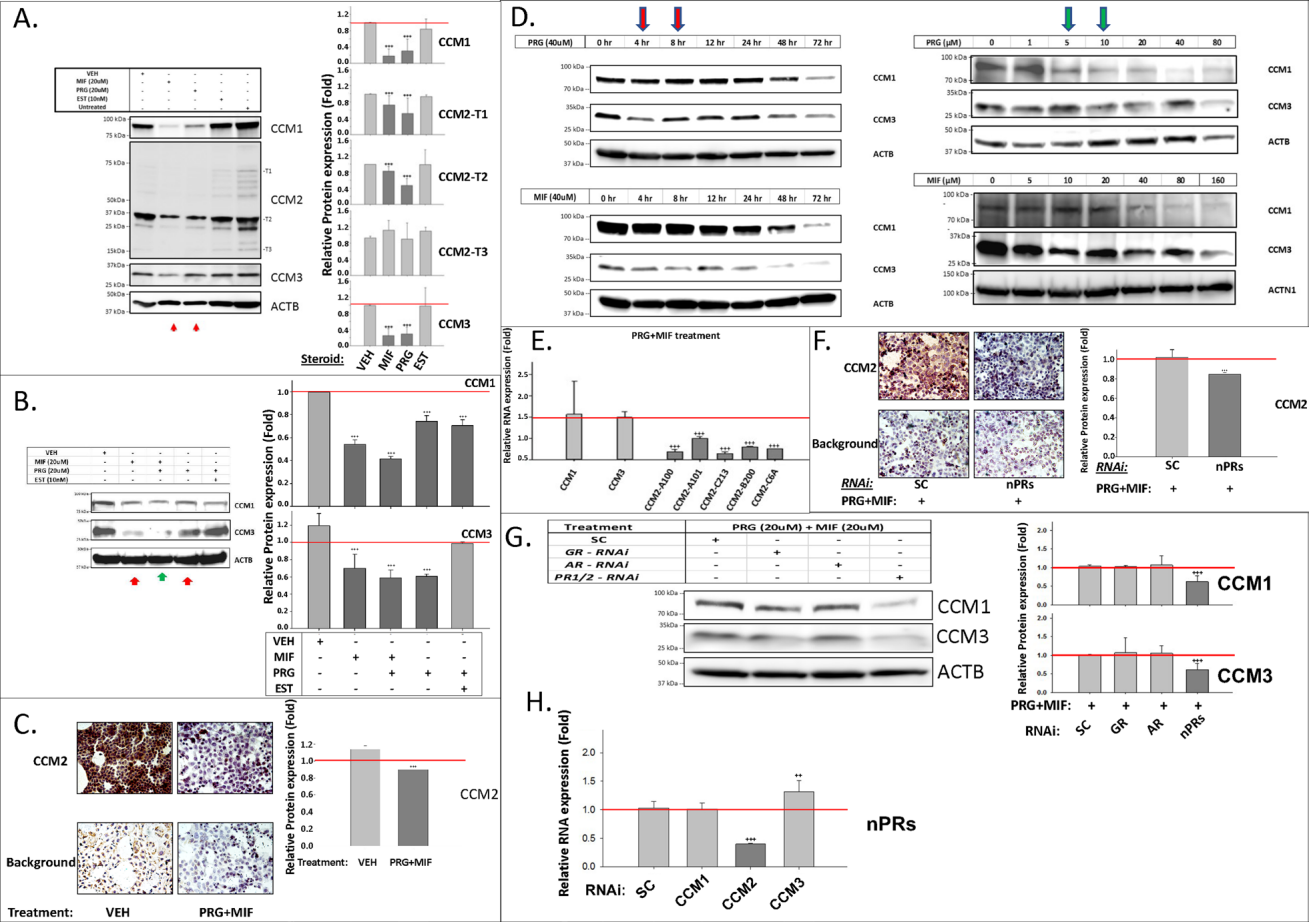
Expression levels of CCMs in nPR(+) T47D breast cancer cells are modulated by both Progesterone (PRG), Mifepristone (MIF), or mPR-specific PRG actions (PRG + MIF). **A** *Expression levels of CCM1, CCM2, and CCM3 in T47D cells can be suppressed independently with either PRG or MIF treatment*. Cells were treated with vehicle control (ethanol/DMSO, VEH), MIF (20 µM), PRG (20 µM), estrogen (EST, 10 nM), or media only (Untreated). Relative protein expression levels of three selected CCM2 isoforms (T1, T2, T3), CCM1, and CCM3 was performed through quantification of band intensities using Western Blots (WB); data were normalized against β-actin (ACTB) followed by vehicle control (red line, right panel, n = 5). **B** *PRG and MIF work synergistically to enhance their inhibitory roles in expression of CCM proteins.* This independent as well as synergistic inhibition was statistically significant (right panel). **C** *Decreased expression levels of CCM2 proteins in T47D cells with mPR-specific PRG treatment.* T47D cells were treated with mPR-specific PRG actions (PRG + MIF, 20 µM each) and stained utilizing immunohistochemistry (IHC) applications with HRP/DAB detection system (left panel). Quantification of CCM2 proteins (right panel, ~ 10,000 ROI/section) was normalized to background staining (red line, n = 4). **D** *Both PRG and MIF can independently induce decreased protein expression of CCM1 and CCM3 in both time and dose-dependent manners.* T47D cells were treated with PRG (40 µM) or MIF (40 µM) for the times indicated in the left panel or treated for 72 h with a series of concentrations (0–80 µM) of PRG or (0–160 µM) of MIF (right panel). **E** *Only mRNA expression of CCM2 isoforms is suppressed by mPR-specific PRG treatment.* T47D cells were treated with mPR-specific PRG actions (20 µM each). Relative RNA expression levels were measured through RT-qPCR (triplicates per experiment, n = 3). **F** *Silencing of classic nPRs further enhances the suppression of* CCM2 *protein expression in T47D cells under mPR-specific PRG actions*. T47D cells were stained with HRP/DAB after silencing nPRs for 24 h, followed by mPR-specific PRG treatment (20 µM each) for another 48 h (left panel). Background controls were not probed with CCM2 antibody to prove the specificity of our CCM2-IHC antibody. Quantification of CCM2 in T47D cells (right panel) was normalized to background staining (red line, n = 4). **G** *Both CCM1 and CCM3 proteins are also sensitized to mPR-specific PRG actions*. T47D cells were treated with RNAi-KD for *nPR*s, androgen receptor (*AR*) or glucocorticoid receptor (*GR*) for 24 h, followed by mPR-specific PRG treatment (20 µM each) for 48 h (Left and right panels, n = 4). **H** *RNA expression levels of classic nPRs is influenced by the CSC in T47D cells.* After silencing *CCM*s (1, 2 or 3) for 48 h, the relative transcriptional changes of *nPR* isoforms in *CCM*s-KD in T47D cells were measured by qPCR (Fold) (n = 3). Red line indicates control baseline for fold change measurements (−/+). **, *** above bar indicates *P* ≤ 0.01 or 0.001 for paired *t*-test, respectively. For additional quantification details see Additional file 1: Fig. S1 legend for IHC details

To elucidate the underlying mechanisms, we systematically tested the effects of major female reproductive hormones, including estrogen (EST), PRG, and its antagonist, MIF, on the expression levels of all three CCM proteins in T47D cells, using Western blots (WB). Our data showed that PRG and MIF, but not EST, have significant negative impacts on the protein expression of CCM1, CCM3 and three major CCM2 isoforms (Fig. 3A). Surprisingly, we discovered that PRG works together with MIF, to further inhibit protein expression of CCM1 and CCM3 (Fig. 3B), as well as CCM2 (Fig. 3C) in T47D cells, suggesting synergistic actions. Although EST can rescue CCM3 stability, when combined with PRG (Fig. 3B), it is incapable of rescuing CCM1, suggesting only a partial positive effect of EST on CSC stability. No synergistic actions on CCM protein expression were observed between EST and PRG (Fig. 3B), suggesting that this newly defined signaling pathway involving the CSC, under mPR-specific PRG actions, likely bypasses classic nPRs. Next, we found that PRG and MIF can suppress protein expression of CCM1/3 in both time-dependent (Fig. 3D, left panel) and dose-dependent manners (Fig. 3D, right panel), within the range of non-cytotoxic concentrations [33], validating our previous observation that both PRG and MIF can independently inhibit the expression of all three CCMs (Fig. 3A–C). Time-course data also indicated that this PRG/MIF-modulated inhibition of CCM proteins utilizes a rapid non-genomic mechanism (Fig. 3D, left panel). Real-time quantitative PCR (RT-qPCR) experiments showed that mPR-specific PRG actions can only inhibit RNA expression of *CCM2* but has no effect on the RNA expression of either *CCM1/3* (Fig. 3E), suggesting that *CCM2* might be the direct target for mPR-specific PRG actions at the transcriptional level, and identifies the central role of *CCM2* in this network. Down-regulation of nPRs has been previously observed at both the transcriptional and translational levels under PRG/MIF separately [32], and although MIF had long been observed as a “partial agonist” to PRG actions with unknown mechanisms [34], this “agonist” role of MIF was thought to only occur in the absence of PRG [34]. Our data represent the first demonstration that either PRG or MIF can work independently, and synergistically to influence expression levels of CCM proteins, in a non-classic mechanism, termed mPR-specific PRG actions, at both the transcriptional and translational levels in Luminal-A T47D cells.

#### PRG and MIF synergistic disruption of the CSC is inhibited by classic nPRs

To investigate the relationship between *nPRs* and *CCM2* under mPR-specific PRG actions, *nPR* genes were silenced by RNAi, followed by mPR-specific PRG treatment in T47D cells. Our results demonstrated significantly decreased expression of CCM2 in *nPR*-silenced cells (Fig. 3F), compared to scrambled controls (SC), suggesting the protective role of *nPRs* on the stability of CCM2 under mPR-specific PRG actions. MIF has been reported to bind to all three major steroid receptors: nPRs (PR1/2), GR (glucocorticoid receptor), and AR (androgen receptors), acting as an antagonist for all three receptors [35, 36]. The degree of nPRs, GRs, and ARs inhibition by MIF are variable, depending on specific cell types [37]. Both ARs and nPRs are highly expressed, but the expression of GRs is low in T47D cells [38]. Using RNAi, we silenced these steroid hormone receptors (*AR, GR, nPRs*) followed by mPR-specific PRG treatment. Interestingly, nPRs-silenced T47D cells showed significantly enhanced inhibitory effects of mPR-specific PRG actions on the expression of CCM1/3 proteins, compared to SC controls (Fig. 3G), supporting the protective role of nPRs on the stability of the CSC under mPR-specific PRG actions. Both AR and GR play no role in the negative effects of mPR-specific PRG actions on the stability of the CSC (Fig. 3G). Knocking down *CCM1, CCM2*, and *CCM3* independently revealed significantly decreased RNA expression levels of *nPRs* only by silencing *CCM2* (Fig. 3H), implying that *nPRs* and *CCM2* reciprocally influence the expression of each other. The novel synergism of mPR-specific PRG actions on the CSC is quite surprising since MIF binds to nPRs with greater affinity than PRG, forming nPRs-MIF complexes, inhibiting nPRs-PRG-associated actions, as an antagonist for nPR-mediated signaling. Therefore, MIF has been widely used as an antiprogestin for contraception and termination of early pregnancies [39]. However, as a type-II antiprogestin, MIF can also act as an agonist in supporting PRG-associated actions in a cell-specific manner with unknown etiology [40].

#### PRG and MIF work synergistically to influence expression of both nPRs and mPRs through the CSC in T47D cells

**Fig. 4:**
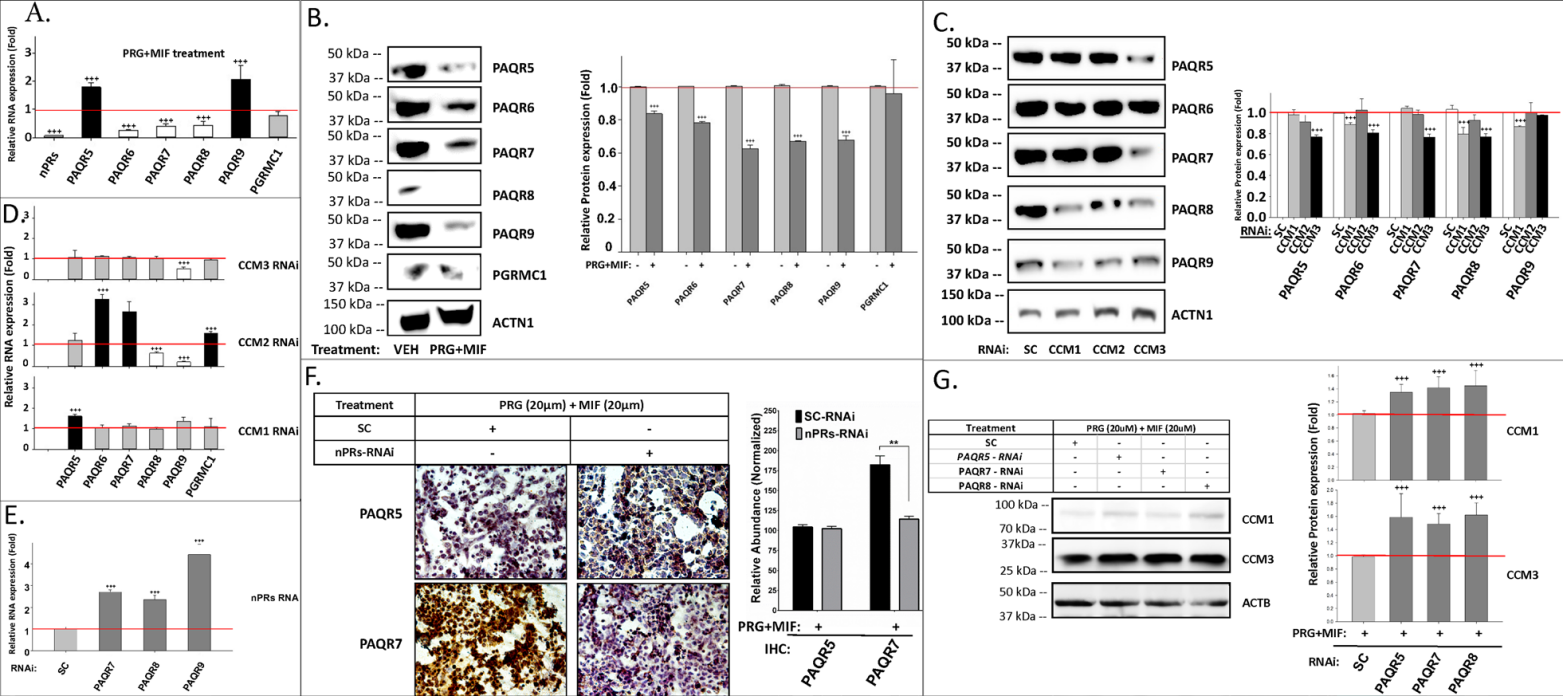
Expression levels of classic nPRs and non-classic mPRs/PAQRs are modulated by mPR-specific PRG actions and the CSC in nPR(+) T47D cells. **A** *RNA expression levels of classic nPRs and mPRs (PAQR5-9) are influenced by mPR-specific PRG actions.* After mPR-specific PRG treatment (20 µM each) for 48 h, RNA expression levels of classic *nPRs*, major *mPRs* (*PAQR5-9*) and *PGRMC1* were measured (n = 3). **B** *Protein expression levels of mPRs (PAQRs) are suppressed by mPR-specific PRG actions* (20 µM each) for 48 h (n = 3). **C** *Protein expression levels of mPRs (PAQRs) are mainly suppressed by silencing CCM1 and CCM3 genes.* After silencing all three *CCM*s for 48 h, protein expression levels of mPRs (PAQR5-9) were performed (n = 3). **D** *RNA expression levels of major mPRs (PAQRs, PGRMC1) are mainly modulated by CCM2*. After silencing all three CCMs for 48 h, altered RNA expression of major *mPRs* were measured (triplicates per experiment, n = 3). **E** *RNA expression levels of nPRs is significantly increased by silencing mPR (PAQRs) genes.* After silencing mPR (*PAQR7-9*) genes for 48 h, RNA expression levels of *nPRs* were observed (triplicate per experiment, n = 3). **F** *Decreased protein expression of PAQR7 by silencing nPRs under mPR-specific PRG actions. nPR*s deficient T47D cells compared to scramble controls were stained utilizing IHC approaches with HRP/DAB (n = 4). **G** *Both CCM1 and CCM3 proteins are stabilized to mPR-specific PRG actions after silencing mPRs in T47D cells.* T47D cells were treated to silence major mPRs (*PAQR5/7/8*) for 24 h, followed by mPR-specific PRG treatment (20 µM each) for an additional 48 h (n = 4). Relative RNA expression changes were measured by qPCR (Fold changes) and normalized to scramble control (red line, triplicates per experiment, n = 3). Relative expression levels of proteins were measured through quantification of band intensities and normalized against either α-actinin (ACTN1) or β-actin (ACTB) followed by SC controls (red line). In all bar plots, red line is the control baseline for fold change measurements (−/+). **, *** above bar indicates *P* ≤ 0.01 or 0.001 for paired *t*-test, respectively

Our previous observations of decreased endogenous CCM1/3 expression initially observed at 4 h under mPR-specific PRG actions (Fig. 3D), suggest the involvement of rapid non-classic PRG actions, most likely through mPRs. Non-genomic effects of PRG mediated by mPRs have been reported to induce rapid intracellular changes [4]. Furthermore, potential crosstalk mechanisms between nPRs and mPRs have been suggested in mediating PRG actions [41], but without any supporting data, yet. In one recent report, mPRα (PAQR7) and β (PAQR8) transactivate PR-2 (nPR isoform 2) in nPR( +) myometrial cells [42], suggesting potential cross-talk between nPRs/mPRs signaling [42]. Our data also correlated expression between CCM proteins (Additional file 1: Fig. S1A–B) and PAQR7 (Additional file 1: Fig. S1D) in breast tumors [6]. RT-qPCR analysis revealed significantly decreased RNA expression levels of *nPRs* under mPR-specific PRG actions in T47D cells (Fig. 4A), in line with a previous report [43]. RNA expression levels among the majority of mPRs (*PAQR6-8*) were significantly decreased, while increased expression of *PAQR5* and *PAQR9* was observed (Fig. 4A). Overall, mRNA levels of *nPRs* and most *mPRs* were remarkably diminished in mPR-specific PRG treated T47D cells, suggesting inhibition of *nPRs* and most *mPRs* by mPR-specific PRG actions at the transcriptional level (Fig. 4A). Surprisingly, WB data demonstrated significantly decreased expression levels of all PAQRs, under mPR-specific PRG actions (Fig. 4B), indicating that the overall expression of all PAQRs can be modulated by mPR-specific PRG actions at both the transcriptional and translational levels (Fig. 4A, B).

#### *The expression levels of nPRs/mPRs/CSC are modulated by intricate feedback mechanisms, suggesting the existence of a shared signaling network in nPR(* +*) T47D cells*

Significantly decreased expression of PAQR proteins was observed mostly in either *CCM1*-Knockdown (KD) or *CCM3*-KD (Fig. 4C) conditions. Interestingly, increased RNA expression levels of *PAQR5* in *CCM1*-KD and decreased RNA expression levels of *PAQR9* in *CCM3*-KD were observed, respectively (Fig. 4D, upper and lower panels). RNA expression patterns of PAQRs were mostly altered in *CCM2*-KD, with increased RNA expression levels of *PAQR6, PAQR7*, and *PGRMC1* while decreased RNA expression levels of *PAQR8* and *PAQR9* were observed (Fig. 4D, middle panel). Thus, our data suggest that *CCM2* might be the key player influencing expression levels of all *mPRs* at both the transcriptional and translational levels, while CCM1/3 proteins only influence the expression levels of mPRs at the translational level.

To further delineate the cellular relationship among nPRs, mPRs/PAQRs, AR, and GR, we next knocked down *AR, GR*, and *nPRs* and discovered no change in mPRs/PAQRs expression either at the protein (Additional file 1: Fig. S3A) or RNA level (Additional file 1: Fig. S3B), indicating nPRs, AR, and GR do not regulate the expression of mPRs/PAQRs either at the protein or RNA level. However, when silencing *mPRs/PAQRs*, significantly increased RNA expression of *nPRs* was observed (Fig. 4E). We also observed that when silencing *nPRs*, a significant decrease in PAQR7 protein expression was observed under mPR-specific PRG actions (Fig. 4F). Together, these data suggest that mPRs might negatively influence *nPRs* expression at the transcriptional level, while *nPRs* expression positively influences mPRs expression at the translational level.

#### mPR-specific PRG actions destabilize the CSC through mPRs

To further delineate the relationship between the CSC and mPRs/PAQRs, we silenced three major mPRs (*PAQR5, 7, 8*) under mPR-specific PRG actions; *mPRs*-silenced T47D cells significantly reduced the inhibitory effect of mPR-specific PRG actions on the expression of CCM1/3 proteins, compared to SC (Fig. 4G), supporting the notion that mPRs destabilize the CSC under mPR-specific PRG actions, opposite to nPRs (Fig. 3G).

### CCM2 is the cornerstone for the stability and functionality of the CSC

In the CSC, it has been previously reported that CCM1 can stabilize ICAP1α and CCM2 through its interactions with both [44–46]. Then, it was found that CCM1/CCM2 can enhance protein stability reciprocally, and this relationship was further extended to all CCM proteins [9], leading us to re-examine the relationship among the three core members of the CSC.

**Fig. 5:**
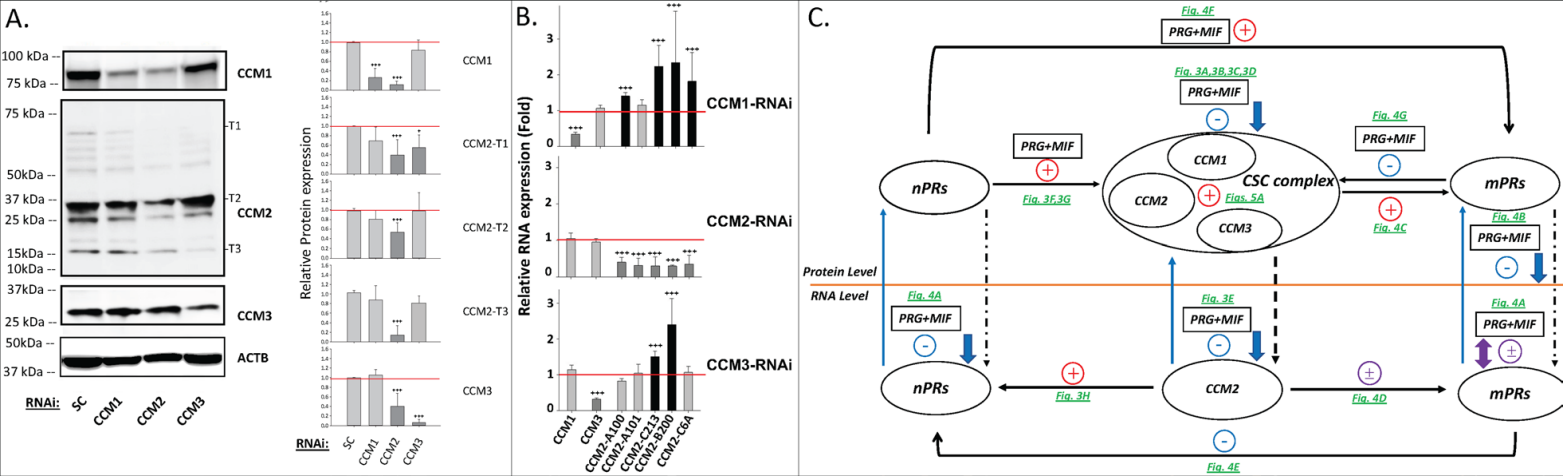
CCM2 is a cornerstone for the essential stability of the CSC. Luminal-A T47D breast cancer cells were treated with siRNA targeting members of the CSC, followed by measurement of their corresponding RNA/protein expression levels. **A** *Silencing of CCM2 decreases the expression level of both CCM1/3 proteins in T47D cells.* T47D cells were treated to silence all three CCMs (1, 2 or 3) for 48 h (Left upper and lower panels). The relative expression levels of CCM1/3 and three major isoforms of CCM2 proteins were measured through quantification of band intensities and normalized against β-actin (ACTB) followed by SC controls, (right panel, n = 3). **B** *Significantly increased RNA levels of CCM2 isoforms in silenced CCM1 (CCM1-KD) and CCM3 (CCM3-KD) T47D cells.* T47D cells were treated to silence all three CCMs (1, 2 or 3) for 48 h and the relative RNA expression levels of *CCM1, CCM3*, and 5 isoforms of *CCM2* were measured by RT-qPCR (Fold, n = 3). **C** *The summarized CmPn signaling network among the CSC, nPRs, and mPRs under mPR-specific PRG actions for Luminal-A breast cancer tumorigenesis.* This diagram details the relationships within the novel CSC-mPRs-PRG-nPRs (CmPn) signaling network, under mPR-specific PRG actions in Luminal-A breast cancer cells. Yellow line separates transcriptional and translational levels. The + symbols represent enhancement,—symbols represent inhibition while ± symbols represent various regulation for the expression of targeted genes/proteins. Red-colored symbols represent positive effects of mPR-specific PRG actions, blue-colored symbols/lines represent negative effects of treatment, while purple-colored symbols/lines represent variable effects. Dark green-colored letters indicate the direct supporting data generated from this work. Arrows indicate effect direction, solid line is the direct impact, dotted line for indirect effects. In all bar plots, red line is the control baseline for fold change measurements (−/+). *, **, *** above bar indicates *P* ≤ 0.05, 0.01, or 0.001, respectively, for paired *t*-test

Surprisingly, silencing of *CCM2* resulted in significantly decreased expression of CCM1/CCM3 proteins, while silencing *CCM1/CCM3* did not influence the expression of CCM2 (Fig. 5A). RT-qPCR however, demonstrated significantly increased RNA expression levels of *CCM2* isoforms with silencing either *CCM1* or *CCM3* while no change of expression of *CCM1/CCM3* RNAs was observed with silencing *CCM2* (Fig. 5B). These results suggest the existence of a feedback regulatory loop influencing the expression of *CCM2*, based on cellular levels of CCM1 and CCM3 at the translational level. Identical results were obtained from 293 T cells (Additional file 1: Fig. S4A1–2), human brain microvascular endothelial cells (HBMVECs) (Additional file 1: Fig. S4B1–2) and zebrafish *Ccm1/Ccm2* mutant strains (Additional file 1: Fig. S4C1–2), further validating our notion that CCM2 is the cornerstone for CSC stability across multiple *in-vitro* and *in-vivo* models.

#### mPRs and nPRs signaling cascades are coupled through the CSC in nPR(+) T47D cells

The novel finding of balanced modulation of the CSC stability through either the positive effects of nPRs or negative effects of mPRs/PAQRs is quite exciting. Our findings not only emphasize the importance of the balance of both, classic and non-classic PRG receptors on CSC functions but also demonstrate the existence of feedback regulations in this relationship. In addition, we also identified the unique role of the CSC in the crosstalk between nPRs/mPRs-specific signaling in Luminal-A breast cancer cells. Our notion is further supported by a previous observation that PRG can act simultaneously on both nPRs and mPRs, and activation of mPR signaling can potentiate hormone-activated nPR isoform (PR-2) expression [42]. This intricate feedback regulatory cascade, defined here as the novel CSC-mPRs-PRG-nPRs (CmPn) signaling network, under mPR-specific PRG actions in Luminal-A breast cancer cells, can be described with an integrated model of the CSC modulating PRG signaling at both classic and non-classic PRG receptors (Fig. 5C). In this model, mPR-specific PRG actions are realized through the balanced efforts between nPRs and mPRs and are further fine-tuned by the CSC. This model is summarized in a schematic representation where key feedback regulatory relationships, at both the transcriptional and translational levels, are illustrated with respective supporting data (Fig. 5C).

### Differential tumorigenic performances between nPR(+) breast cancer cells under mPR-specific PRG actions

#### Temporally differential effects of mPR-specific PRG actions on migration potential of Luminal-A breast cancer cells

**Fig. 6:**
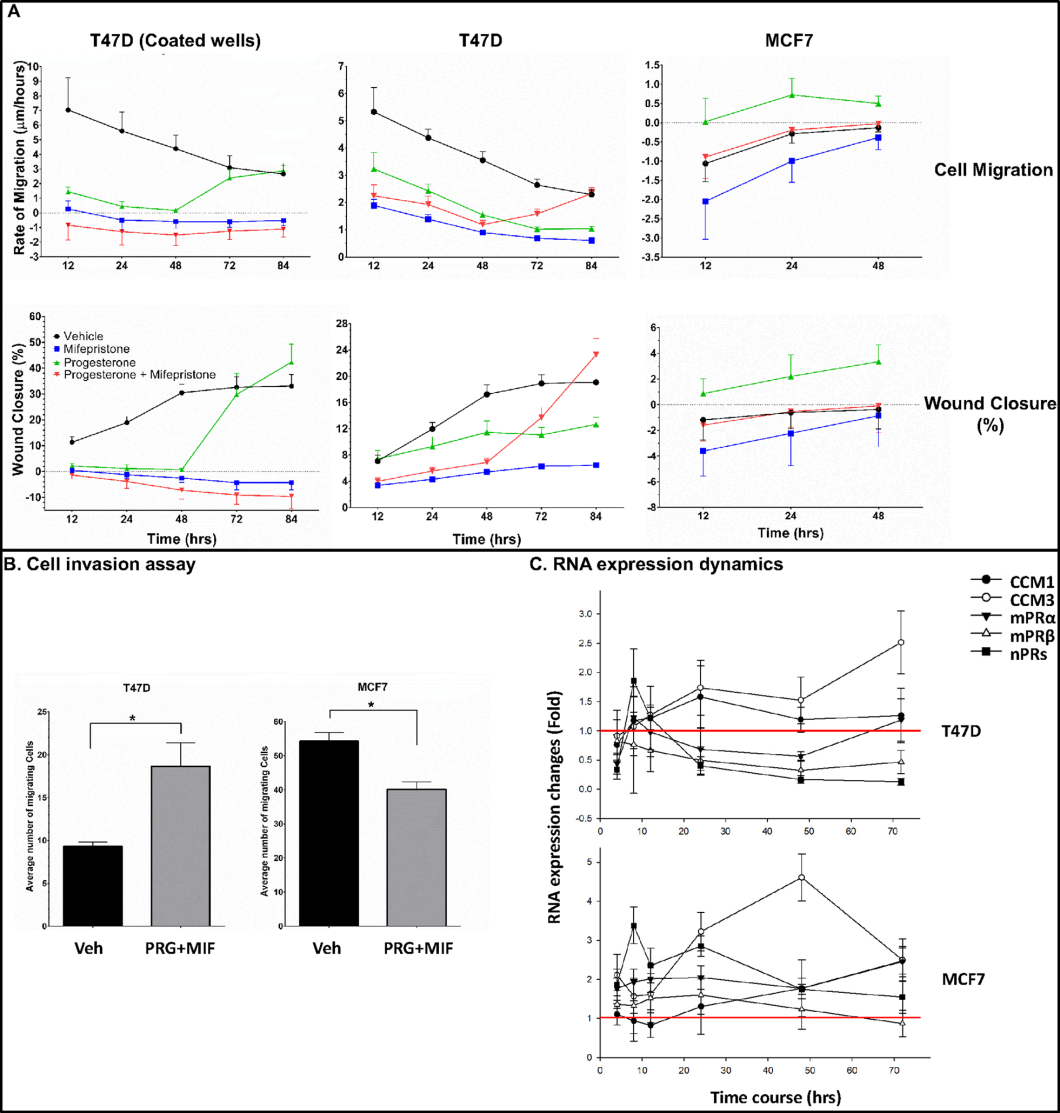
Tumorigenic assessment of two Luminal-A breast cancer cell lines under mPR-specific PRG actions. Two Luminal-A breast cancer cell lines, T47D and MCF7, were treated in triplicates in FBS-free medium containing vehicle control (EtOH, DMSO), MIF (20 µM), PRG (20 µM), or mPR-specific PRG treatment (MIF + PRG, 20 µM each). **A** T47D cell migration was measured with all hormone treatments when plated on Collagen-I coated plates, (top left panel). T47D cells were also cultured on non-coated plates (top middle panel). MCF7 cell migration was measured with all hormone treatments on non-coated plates (top right panel). *Similar trends in wound healing experiments were observed.* T47D wound closure was measured with all hormone treatments, when plated on Collagen-I coated plates (bottom left panel). T47D cells were also cultured on non-coated plates (bottom middle panel). MCF7 cells wound closure was also measured on non-coated plates (Bottom right panel). **B** Cell invasion assays for T47D cells under combined mPR-specific PRG actions (left panel) and MCF7 under mPR-specific PRG actions (right panel). **C** Temporal RNA expression patterns of 5 key CmPn network genes, *CCM1*, *CCM3,* *nPRs*, *mPRα* (PAQR7) and *mPRβ* (PAQR8) genes, were demonstrated in a time course analysis of the response to mPR-specific PRG treatment for T47D cells (top panel) and MCF7 cells (bottom panel). RNA expression of both *nPR* and *mPRs (PAQR7/PAQR8)* genes in T47D cells (top panel) and MCF7 cells (bottom panel). Statistical significance for migration and wound healing was performed using two-way ANOVA or with students *t*-test for invasion assay. All experiments were performed with triplicates per experiment (n = 3); for all panels, * above bars indicate *P* ≤ 0.05

T47D cells (Luminal-A subtype) displayed a significant decrease in cell migration, at early time points, with all hormone treatments, compared to vehicle controls, when plated on collagen-I coated wells, with a surprising surge of cell migration in the PRG treated group only after 48 h (Fig. 6A, top left panel). Interestingly, the same significant decreased cell migration trend, at earlier time points, was also seen when T47D cells were cultured on non-coated wells, compared to vehicle controls, but this time with a surge of cell migration observed under mPR-specific PRG actions at 48 h (Fig. 6A, top middle panel). MCF7 cells (also Luminal-A subtype) displayed the opposite trend observed in T47D cells, with only a slight increase in cell migration observed in the PRG treated group (Fig. 6A, top right panel). These results suggest that Luminal-A breast cancer cells have different migration potential under mPR-specific PRG actions and suggest cellular heterogeneity within Luminal-A breast cancer cells. These results also reinforce the existence of crosstalk between integrin and PRG receptors-mediated signaling cascades.

#### Temporal differential effects of steroid hormones on wound healing ability of Luminal-A cells

Similar trends in wound healing experiments were also observed in which T47D cells displayed a significant decrease in wound closure with all hormone treatments, at earlier time points, compared to vehicle controls, when plated on collagen-I coated wells, with a surprising surge of wound closure at 48 h with PRG (Fig. 6A bottom left panel), correlating with the observed increased cell migration also seen in the PRG treated group after 48 h (Fig. 6A, top left panel). The same significant decreased wound closure trend, at earlier time points, was also seen when T47D cells were cultured in non-coated wells, compared to vehicle controls, but this time a surge of wound closure was seen in the mPR-specific PRG treated group after 48 h (Fig. 6A bottom middle panel), correlating with the increased cell migration also seen (Fig. 6A top middle panel). MCF7 cells displayed the opposite trends observed in T47D cells, with only a slight increase in wound closure capabilities observed in the PRG treated group (Fig. 6A, Bottom right panel), also correlating with the increased cell migration seen in the PRG treated group (Fig. 6A, top right panel). In sum, the different migration and wound healing performances between Luminal-A breast cancer cells, T47D and MCF7, suggest there are intrinsic differences among their cellular signaling pathways under mPR-specific PRG actions. It is worthy to note that observations after 48 h timepoint are most likely influenced by nutrient depletion in the media as well as prolonged treatment stress which will be further explored in the future. Furthermore, the different wound healing performances of T47D cells, on coated and non-coated wells, reaffirm the existence of crosstalk between integrin signaling and the CmPn signaling network.

#### Differential effects of steroid hormones on cell invasion of Luminal-A breast cancer cells

Utilizing cell invasion assays, a significantly increasing trend of cell invasion for T47D cells under mPR-specific PRG actions, was observed (Fig. 6B, left panel) while decreasing cell invasion trends were observed for MCF7 cells under equal treatment, compared with vehicle controls (Fig. 6B, right panel). In sum, opposite performances of cell invasion between Luminal-A breast cancer cells, T47D and MCF7, further validate our previous notions of their intrinsic differences among cellular signaling pathways under mPR-specific PRG actions.

#### Temporal RNA expression of key factors within the CmPn signaling network

mPR-specific PRG actions can induce RNA expression of both *CCM1* and *CCM3* in T47D cells (Fig. 6C, top panel) with similar results obtained in MCF7 cells (Fig. 6C, bottom panel), although *CCM1* induction, in MCF7, was not as drastic. RNA expression of both *nPR* and *mPRs (PAQR7/PAQR8)* was dramatically decreased in T47D cells (Fig. 6C, top panel), while relatively unchanged in MCF7 cells (Fig. 6C, bottom panel). These differences in expression patterns might explain the differential tumorigenic performances previously observed (Fig. 6A, B). Additionally, we observed that while no induced RNA expression of *AR* was observed, inducible RNA expression of *GR* was observed under mPR-specific PRG actions in both Luminal-A breast cancer cells (Additional file 1: Fig. S5), suggesting *GR* induction may result from its strong binding affinity to MIF, unrelated to their differential tumorigenic performances (Fig. 6A, B).

### Sub-cellular compartmentation of key factors of the CmPn network in T47D cells under mPR-specific PRG actions

Immunofluorescent (IF) imaging revealed modulation in the relative intensity of CCM1 staining in T47D cells, under mPR-specific PRG actions, with decreased expression observed at 72 h (Additional file 1: Fig. S6A-1). Additionally, the majority of CCM1 resides in the cytoplasm with no drastic changes in the localization of CCM1 (Additional file 1: Fig. S6A-1). Similarly, CCM3 staining in T47D cells, under mPR-specific PRG actions, also showed decreased expression observed at 72 h with the majority of CCM3 residing in the cytoplasm with no drastic changes in the localization of CCM3 (Additional file 1: Fig. S6A-2). Surprisingly, more PAQR8 staining was observed inside the nucleus at 72 h in T47D cells, under mPR-specific PRG actions (Additional file 1: Fig. S6A-3), suggesting possible nuclear localization of mPRs in nPR( +) T47D cells, similar to our previous observations in TNBC cells [47, 48].

#### Temporally altered expression of key factors of the CmPn signaling network in T47D cells under mPR-specific PRG actions

Temporal modulation of CCM1 in T47D cells, under mPR-specific PRG actions, revealed a gradual decrease in the relative intensity of CCM1 staining in T47D cells with decreased expression observed after 4 h (Additional file 1: Fig. S6B-1), opposite to our previous observations in TNBCs [47, 48]. Like CCM1, relative expression of total CCM3 proteins in T47D cells, under mPR-specific PRG actions, also revealed a gradual decrease in the relative intensity of CCM3 observed after 8 h (Additional file 1: Fig. S6B-2). Alternatively, relative expression of total PAQR8 proteins in T47D cells, under mPR-specific PRG actions, revealed an increased intensity observed at 24 h (Additional file 1: Fig. S6B-3), in agreement with our previous observations in TNBCs [47, 48]. Together, these results suggest that protein expression of key players of the CmPn signaling network are capable of being modulated under mPR-specific PRG actions in nPR( +) T47D cells, in line with our previous findings (Fig. 3A, B).

### *Relationship among nPRs/mPRs and the CSC in hormone treated nPR(* +*) T47D cells using omic approaches*

Bioinformatics analyses identified saturable, high-affinity binding sites for PRG [4, 7], but no binding site for MIF in mPRs has been defined yet [4]. Unlike mPRs, which bear the same binding site for both PRG and MIF, nPRs higher binding affinity to MIF might differentiate classic PRG actions from non-classic mPR-specific PRG actions [4, 7]. Due to the relationship mentioned above, MIF competes with PRG for the same binding sites on nPRs as an antagonist for nPR-mediated signaling. In contrast, MIF works synergistically with PRG in mPR-mediated signaling as an agonist, and we have therefore defined MIF + PRG treatment as mPR-specific PRG actions.

To define MIF binding partners in the synergistic action with PRG, we examined expression patterns of T47D cells under MIF-only or mPR-specific PRG actions, at both the transcriptional and translational levels using high throughput omics, including RNAseq and Liquid Chromatography-Tandem Mass Spectrometry (LC–MS/MS). Among the identified differentially expressed genes (DEGs), we were able to visualize hierarchical clustering and found similar patterns in both intersection and union of DEGs between the two treatments (Additional file 1: Fig. S7A–B). Similar patterns in observed DEGs were found between the two treatments (Additional file 1: Fig. S7C–D), suggesting shared signaling cascade variations between them. Finally, several key signaling cascades involved in tumorigenesis, observed with both treatments, were frequently perturbed through KEGG pathways analysis including cellular senescence, cell cycle, p53, microRNA’s in cancer, AMPK, MAPK, WNT, RAS, and hormone signaling pathways (Additional file 1: Fig. S7E–F). Similar results were observed in nPR(−) breast cancer cells [47, 48], leading us to propose that MIF binds to mPRs allosterically with PRG, and works synergistically with PRG, in a similar fashion. Interestingly, our RNAseq data (available upon request) confirmed decreased expression of *CCM2* isoforms previously observed under mPR-specific PRG actions (Fig. 3E). Similarly, in the same RNAseq dataset, we also observed decreased expression of *PAQR8* in both MIF and mPR-specific PRG treated samples as well as increased expression of *PAQR5* in accordance with our RT-qPCR data (Fig. 4A), further validating our results and supporting our proposed CmPn model (Fig. 5C).

Using a similar approach, we analyzed our proteomic data (available upon request) with additional samples from silencing three *CCM* genes. After comparing each *CCMs*-KD sample to its respective control, we pooled the significant, differentially expressed proteins (DEPs) to represent a disrupted CSC since all 3 proteins are required to form a fully functional CSC [9–11]. Hierarchical clustering showed similar patterns between both hormone treatments (Additional file 1: Fig. S8A–B), supporting our RNA data and further emphasizing shared regulatory mechanisms of mPR-specific PRG actions at both the transcriptional and translational levels. However, there are significant differences in the CSC disrupted group (Additional file 1: Fig. S8C) compared to both hormone-treated groups, indicating the independent role of the CSC in the CmPn signaling network (Additional file 1: Fig. S8A–C). Interestingly, we found that all three treatment groups displayed a larger amount of significantly down-regulated compared to up-regulated proteins (Additional file 1: Fig. S8D–F), which was not the case for our transcriptomics analysis, suggesting a potential feedback auto-regulation among potential key players of the CmPn network. Similar to our KEGG analysis performed using RNAseq data, our proteomics data for both hormone treatments (Additional file 1: Fig. S8G–H), also showed differentially expressed proteins in the cell cycle, AMPK, and apoptosis signaling pathways (compare Additional file 1: Figs. S7E–F and S8G–H). Shared pathways between hormone treatments at the translational level included proteoglycans in cancer (Additional file 1: Fig. 8G–H), further supporting the intricate balance between the CSC and mPR-specific PRG actions during breast cancer tumorigenesis. When evaluating a disrupted CSC, it was no surprise to see that the greatest number of DEPs are involved in pathways in cancer signaling cascades, solidifying the CSC’s novel role in tumorigenesis (Additional file 1: Fig. S8I). Additionally, Ras signaling, apoptosis, tight junction, and regulation of actin/cytoskeleton were among some of the other signaling pathways affected by a disrupted CSC in Luminal-A T47D breast cancer cells (Additional file 1: Fig. S8I). Overall, our proteomics data further supports our RNAseq conclusions that mPR-specific PRG actions modulate the expression of key factors of the CmPn network in a synergistic fashion.

### Relationship of nPRs/mPRs/CSC in the CmPn signaling network under mPR-specific PRG actions in Luminal-A T47D breast cancer cells using systems biology

**Fig. 7:**
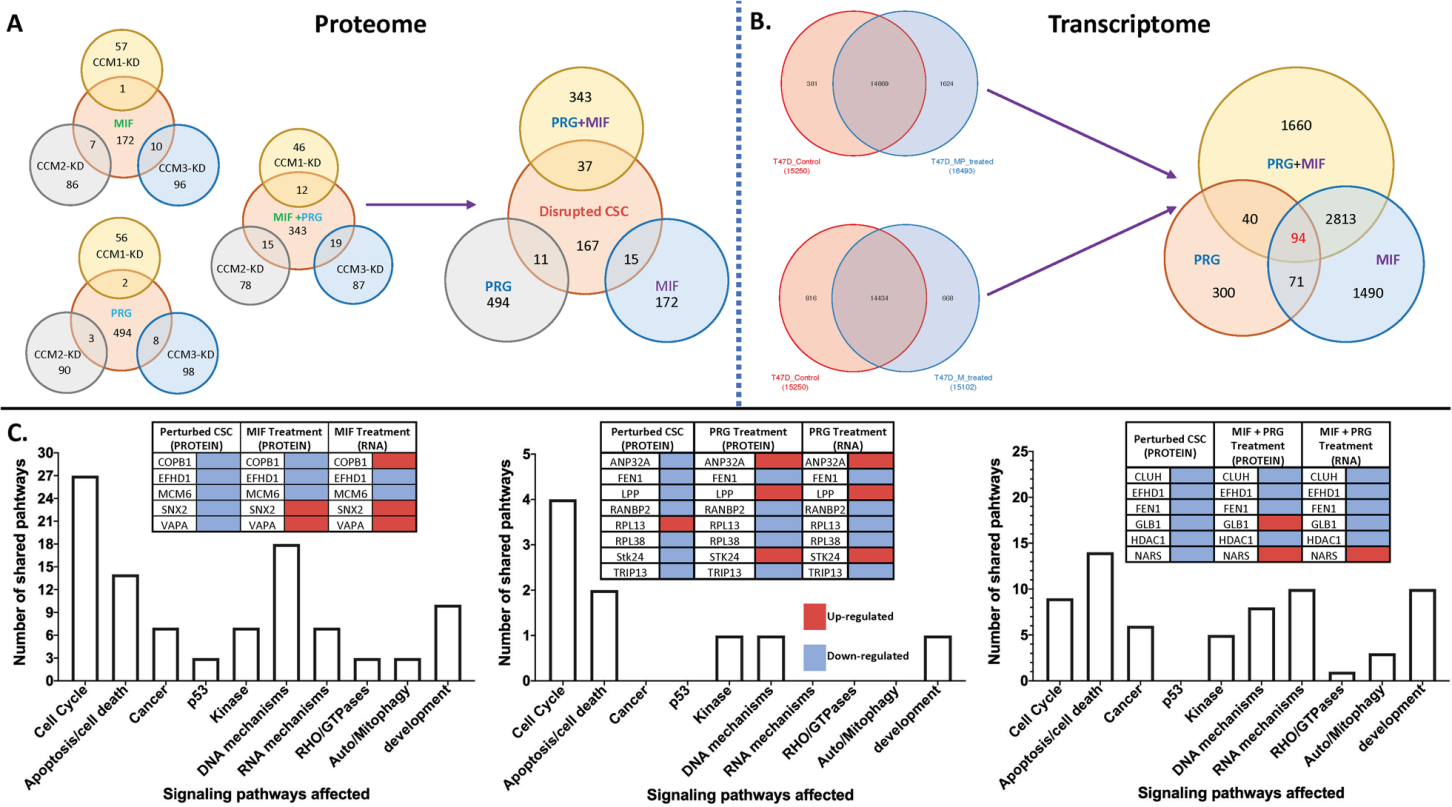
Overlapped Differentially Expressed Genes (DEGs)/Proteins (DEPs) using systems biology approaches. Gene overlaps were calculated at both the **A** translational, **B** transcriptional, and **c** both transcriptional and translational levels to elucidate any shared signaling pathways between treatment groups. Proteomic overlaps (panel **A**) are demonstrated for both CSC disturbance as well as hormone treatments. Individual comparisons between hormone treatments and CCM knockdowns are illustrated, and the combined knockdowns (disrupted CSC) compared to hormone treatments. Transcriptomic overlaps (panel **B**) are demonstrated for hormone treatments. Individual comparisons between hormone treatments are illustrated as well as the combined hormone treatments comparison. Pathway classification and functional enrichment was performed on overlapped genes at both the proteome and transcriptome levels to evaluate any shared signaling mechanisms altered through a disturbed CSC and hormone treatments (panel **C**). For all analysis, PRG-only comparisons (proteome and transcriptome) were conducted using published proteomic/transcriptomic databases to assemble a working database that could be compared to our MIF and MIF + PRG treated samples. If available, fold changes and *p*-values were obtained from the databases to increase the depth of analysis of the multi-omics comparison. Raw data can be provided upon request

In addition to our preliminary differential expression analysis of CmPn members across various breast cancer tumors (Fig. 1), we recently identified differential expression patterns of the CSC are correlated with certain types and grades of major human cancers, especially in breast cancers [6, 11, 49], further validating a role for the CSC during breast cancer tumorigenesis. To further delineate the role of key players within the CmPn network under mPR-specific PRG actions, we compared our two omic approaches to identify shared altered signaling pathways at both the transcriptional and translational levels. Before doing this, we wanted to identify the signaling cascades and genes altered with each respective hormone combination. PRG-only comparisons (proteome and transcriptome) were assembled using published data [50, 51] to create a working database that could be compared to our mPR-specific PRG treatment results. First, we identified DEGs/DEPs at both the translational (Fig. 7A) and transcriptional (Fig. 7B) levels for each hormone treatment. Once identified, we matched any shared DEGs/DEPs among hormone treatments as well as for our disrupted CSC model. We identified 5 DEGs/DEPs overlapped with a disrupted CSC and MIF-only treatment (weaker mPR-specific PRG actions, Fig. 7C, left panel), 8 genes overlapped with a disrupted CSC and PRG-only treatment (Fig. 7C, middle panel), while 6 overlaps were identified with a disrupted CSC and mPR-specific PRG treatment (Fig. 7C, right panel). Using functional enrichment data for initially generated comparisons, we then identified shared pathways among the three hormone treatment groups shared with a disrupted CSC (Fig. 7C), which included cell cycle, apoptosis/cell death, kinase, DNA mechanisms, and development signaling cascades. Shared Signaling between a disrupted CSC, MIF-only, and mPR-specific PRG actions included cancer, RHO/GTPases, RNA mechanisms, and autophagy/mitophagy pathways (Fig. 7C). The 15 altered genes identified in our omics approaches were then evaluated to assess their potential as Luminal-A specific breast cancer biomarkers.

#### Candidate biomarkers associated with a perturbed CmPn signaling network in nPR(+) T47D cells

**Fig. 8:**
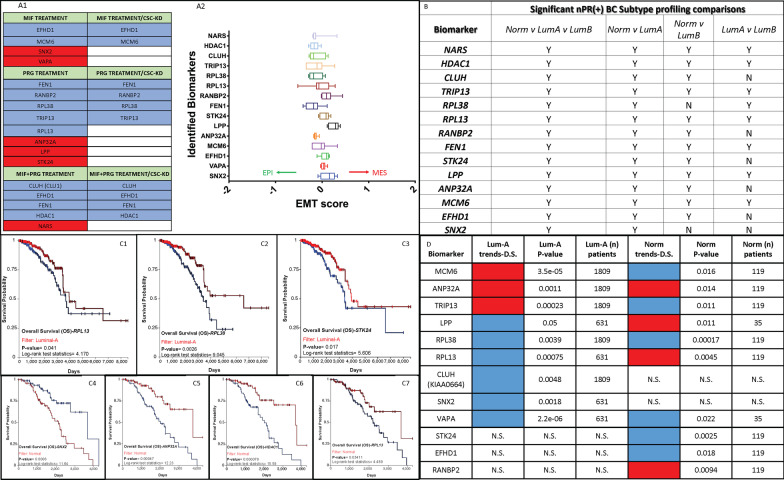
Prognostic effects for our identified candidate biomarkers utilizing multiple luminal-like breast cancer clinical databases. Using systems biology, we analyzed metastasis transformation and survival/expression data for our identified candidate biomarkers using clinical tumor expression data. **A** *Candidate biomarkers associated with a disrupted CmPn signaling network in Luminal-A T47D cells during tumorigenesis.* The table illustrated in the left panel details the shared DEGs/DEPs under hormone treatments (panel **A1**, left column) and hormone treatments combined with CSC-KD (panel **A1**, right column) in this study. Treatments are colored green in the table, genes/proteins are color-coded red (up-regulated) and blue (down-regulated), compared to vehicle controls. Additionally, we further examined the candidate biomarkers and generated comparable EMT score signatures for epithelial to mesenchymal transition (EMT) potential for our identified candidate biomarkers using 7 publicly available pan-Breast cancer cohorts (panel **A2**). **B** *Summarized RNAseq expression profiling of identified diagnostic candidate biomarkers for Luminal-like breast cancer tissues using TCGA.* Candidate biomarkers shared at both the RNA/Protein levels under mPR-specific PRG treatments (columns 1) were further analyzed utilizing the TCGA database to assess their potential as diagnostic biomarkers between luminal-like breast cancers. Abbreviations: BC, Breast Cancer; Norm, Normal breast cancer subtype; LumA, Luminal-A subtype; LumB, Luminal-B subtype; Y, yes to significant expression differences; N, no to significant expression differences. **C1–3** *Prognostic effects for identified candidate biomarkers was assessed utilizing microarray data from Luminal-A breast cancer patients from TCGA.* Publicly available microarray data from 1,236 breast cancer patients was analyzed to integrate gene expression and clinical data simultaneously to generate the displayed Kaplan–Meier (KM) survival curves. Breast cancer patients were filtered to only analyze patient samples classified as Luminal-A subtype (identical to T47D cells). **C4–7** *Prognostic effects for identified candidate biomarkers utilizing microarray data of normal-like breast cancer tumors from TCGA.* Breast cancer patients were filtered to only analyze patient samples classified as Normal Breast cancer subtype [ER(+)/nPR(+)/HER2(-)]. For all survival curves, logrank *P*-values are calculated and displayed as well as logrank test statistics which were automatically calculated by the software using default parameters. **D** *Summarized prognostic effects for our identified candidate biomarkers utilizing microarray data of breast cancer patients using KMplotter.* Publicly available microarray data (22,277 probes) from 1,809 breast cancer patients was analyzed using kmplotter to integrate gene expression and clinical data simultaneously to generate the displayed KM survival curves. Breast cancer patients were filtered to only analyze patient samples classified as either ER(+)/nPR(+)/HER2(-)/Luminal-A subtype (T47D cells) or ER(+)/nPR(+)/HER2(−)/Normal subtype (identical receptors’ status as Luminal-A). Trends associated with decreased survival for Luminal-A breast cancer tissues are shown in column 2, followed by the corresponding *P*-values (automatically calculated by the software using default parameters) and number of patients(n) in columns 3 and 4, respectively. Trends associated with decreased survival for normal breast cancer tissues are shown in column 5, followed by the corresponding *P*-values and number of patients(n) in columns 6 and 7, respectively. For Panels **A1**, **C1**–**7**, and **D**, red color indicates up-regulation of gene expression, while blue color indicates down-regulation of gene expression

Using our established systems biology pipeline, we evaluated our omics data to identify shared DEGs/DEPs under various hormone treatments (Fig. 8, panel A1, left column) and hormone treatments combined with CSC-KD (Fig. 8, panel A1, right column) to identify any novel candidate biomarkers for Luminal-A breast cancer cells with a disrupted CmPn signaling network. This analysis resulted in the identification of 15 candidate biomarkers with altered gene expression detailed for all treatment conditions. Generally, there were more down-regulated DEGs/DEPs seen under both treatment conditions, and only down-regulated DEGs/DEPs were shared between the various treatment conditions (Fig. 8, panel A1). Up-regulated DEGs/DEPs in Luminal-A breast cancer cells were only identified in hormone treatment groups, suggesting that a disrupted CSC suppresses the expression of up-regulated CmPn players observed under steroid treatments only (Fig. 8, panel A1, right column).

Using our identified candidate biomarkers, we further explored epithelial to mesenchymal transition (EMT) potential of these markers in human breast cancer tissue samples. To do this, we averaged EMT scores from 7 publicly available pan-breast cancer cohorts [20–26] for the identified candidate biomarkers. Upon evaluation, we generated comparable EMT score signatures, demonstrating that these candidate biomarkers are most likely not major drivers for EMT transition during breast cancer tumorigenesis (Fig. 8, panel A2).

#### *Differential expression patterns of our candidate biomarkers in nPR(* ±*) breast cancer tissues*

To explore expression patterns of our identified candidate biomarkers, we utilized publicly available breast cancer tumor tissue gene expression data (microarray) [27] that was divided into two groups based on nPR(±) status. Eight of our candidate biomarkers were found to be significantly down-regulated in nPR(+) tumor tissues, compared to nPR(-) tumor tissues (Additional file 1: Fig. S9A). We observed the same down-regulated trends for 6/8 markers with a disrupted CmPn signaling network in all of our treatment conditions in T47D cells (Fig. 8, panel A1), allowing us to preliminarily classify these genes as Luminal-A intrinsic biomarkers. Interestingly, *ANP32A* and *STK24* were observed to be significantly up-regulated with PRG treatment in nPR(+) T47D cells (Fig. 8, panel A1), preliminarily classifying these genes as PRG-inducible biomarkers in Luminal-A breast cancer cells. Furthermore, two of our identified candidate biomarkers, *HDAC1* and *NARS*, were found to have similar expression patterns in nPR(±) tumor tissues (data not shown). However, after disruption of the CmPn signaling network under mPR-specific PRG actions, expression of *HDAC1* was observed to be down-regulated (Fig. 8, panel A1), while *NARS* was significantly up-regulated in nPR(+) T47D cells (Fig. 8, panel A1), preliminarily classifying these genes as mPR-responsive biomarkers in Luminal-A breast cancer cells.

Additionally, 5 of our candidate biomarkers were found to be significantly higher in nPR(+) breast tumor tissues, compared to nPR(-) breast tumor tissues (Additional file 1: Fig. S9B). We observed the same up-regulated trends for 3/5 candidate biomarkers (*LPP, VAPA, SNX2*) with a disrupted CmPn signaling network in T47D cells with all our treatment conditions (Fig. 8, panel A1), adding these genes to our preliminary list of Luminal-A intrinsic biomarkers. Interestingly, however, *EFHD1* and *RANBP2* were observed to be significantly down-regulated in MIF/MIF + PRG and PRG treatment conditions, respectively, in nPR(+) T47D cells (Fig. 8, panel A1) preliminarily classifying RANBP2 as a PRG-repressive, and EFHD1 as an mPR-specific PRG-repressive biomarker. Overall, we preliminarily identified a total of 9 Luminal-A intrinsic, 2 PRG-inducible, 1 PRG-repressive, 1 mPR-specific PRG-repressive, and 2 mPR-responsive candidate biomarkers with altered gene expression, in Luminal-A breast cancer tissues.

#### Differential expression patterns of our candidate biomarkers in Luminal breast cancer tissues

Like our preliminary analysis of expression levels of key members of the CmP network (Fig. 1), we repeated our analysis to investigate the expression levels of our candidate biomarkers among Luminal-A/B breast cancer tissues. For our preliminary analysis, we included Luminal-A, normal-like, and Luminal-B tissues. Our analysis identified significant differential expression patterns for almost all our identified candidate biomarkers (except for *VAPA*), assessed using One-way ANOVA, among all 3 luminal-like breast cancers (Additional file 1: Fig. S10A). Next, we wanted to assess our biomarkers comparing Luminal-like breast cancers in a pairwise fashion. Similar to Additional file 1: Fig. S10A, our analysis confirmed significant differential expression patterns for almost all our identified candidate biomarkers (again except for *VAPA*), between normal and luminal-A breast cancers (Additional file 1: Fig. S10B). Next, we assessed expression levels between normal and Luminal-B breast cancers, and observed significant differential expression patterns for 12/15 biomarkers, between normal and luminal-B breast cancers (Additional file 1: Fig. S10C). Finally, when assessing our biomarkers only between Luminal-A and Luminal-B subtypes, we obtained significant differential expression patterns for 8/15 biomarkers between luminal-A and luminal-B breast cancers (Additional file 1: Fig. S10D). Together, these results provided further validation for 14/15 (no confirmation for *VAPA*) of our potential diagnostic biomarkers in distinguishing between Luminal-like breast cancers utilizing clinical tissue data and are summarized in Fig. 8B.

#### Dual validation of our candidate biomarkers between luminal breast cancer tissues for their prognostic effects

We next sought to evaluate the prognostic effects of our candidate biomarkers, using two independent databases (TCGA and KMplotter), by integrating gene expression and clinical data simultaneously to generate Kaplan–Meier survival curves [27, 28]. First, using TCGA, Breast cancer patients were filtered to only analyze patient samples classified as Luminal-A subtype. For 3/14 of our preliminarily identified biomarkers, a worst prognostic effect was observed with lower expression of *RPL13, RPL38*, and *STK24* (Fig. 8C, panels C1–C3). Next, we repeated our analysis, but breast cancer patients were filtered to only analyze patient samples classified as normal breast cancer subtypes. Our analysis confirmed the down-regulation of *RPL13*, and newly identified down-regulation of *HDAC1, ANP32A,* and the up-regulation of *SNX2* as having the worst prognostic effects in normal-subtype breast cancer tumors (Fig. 8C, panels C4–C7). We repeated our analysis, but this time using KMplotter in which again breast cancer patients were filtered to only analyze patient samples classified as Luminal-A subtypes. We obtained significant KM survival curves for 9/14 biomarkers (Fig. 8D and Additional file 1: Fig. S11, panels 1–9). Interestingly, identical to our results with TCGA, this independent cohort confirmed a worst prognostic effect with lower expression of *RPL38* (Fig. 8D and Additional file 1: Fig. S11, panel 5) and *RPL13* (Fig. 8D and Additional file 1: Fig. S11, panel 6). Surprisingly, in our omics data, we also observed down-regulation for *RPL13* expression (PRG treatment only) and *RPL38* expression (PRG treatment and *CSC*-KD) in nPR(+) T47D cells (Fig. 8, panel A1), validating their role as intrinsic biomarkers in Luminal-A breast cancers. We repeated our analysis using KMplotter but this time filtered breast cancer patient samples to only analyze normal breast cancer subtypes as we did with our TCGA data. Similar to our Luminal-A results, we obtained significant KM survival curves for 10/14 biomarkers (Fig. 8D and Additional file 1: Fig. S12, panels 1–10). Interestingly, contrary to our results with TCGA, this independent cohort demonstrated a worst prognostic effect with increased expression of ANP32A (Fig. 8D and Additional file 1: Fig. S12, panel 9) and *RPL13* (Fig. 8D and Additional file 1: Fig. S12, panel 1), demonstrating the heterogeneity of luminal-like breast cancers and the importance of a dual validation through independent cohorts when assessing survival curves. Together, these results indicate the complexity of the CmPn signaling network with dual roles during breast cancer tumorigenesis. Finally, our results elucidate the great potential of our candidate intrinsic biomarkers (dual validated) for prognostic applications among heterogeneous Luminal-A breast cancers.

## Discussion

In this study, we identified that the CSC has a major impact on PRG signaling through modulating crosstalk between classic nPRs and non-classic mPRs in nPR(+) Luminal-A breast cancer cells, providing novel insights into cellular coupling between CSC-mediated signaling and PRG modulated pathways via its receptors involved during breast cancer tumorigenesis. These findings could revolutionize the current understandings of molecular mechanisms of breast cancer tumorigenesis, leading to new therapeutic strategies. In sum, we are the first group to identify the existence of this CmPn signaling network in nPR(+) breast cancers, which stems from the core CmP network identified in our TNBC works.

There has been supporting evidence that PRG promotes cell proliferation [52] and inhibits cell death in T47D cells, suggesting it may act pro-oncogenic in Luminal-A breast cancers [53]. It has been speculated that PRG and its cellular metabolites promote cell proliferation through induced activation of MAPK signaling in both nPR( ±) breast cancer cell lines, which was confirmed in our omics data. PRG-mediated cell proliferation in both nPR( ±) breast cancer cells are independent of classic PRG/EST receptors [54], suggesting that mPRs may play a role in this signaling pathway.

As an antagonist of PRG, MIF certainly earned its candidacy early on as an initial therapeutic strategy for the treatment of breast, prostate, ovarian, endometrial cancers, and endometriosis, and is currently in many active clinical trials [40]. It was demonstrated that elevated levels of MIF can enhance the growth inhibition and induction of apoptosis triggered by high doses of PRG in nPR( ±) breast cancer cells [55, 56]. It has also been reported that a clinically relevant dose of MIF significantly improves the treatment efficacy of cisplatin-paclitaxel chemotherapy regimens for human ovarian carcinoma cells [57]. However, many contradictory results have been reported regarding whether MIF has growth inhibition or stimulation for hormone-dependent breast cancer cells, as an anti-progestin [58]. By screening 10 cancer cell lines with various genetic backgrounds, regardless of tissue of origin and hormone responsiveness, it was found that the anti-proliferative activity of MIF in cancer cells is independent of nPRs [59]. Similarly, the cellular effects of MIF on proliferation are also widely observed [60, 61]. It seems that the pro- or anti-proliferative activity of MIF is determined by specific cell types [40], the concentration of MIF, as well as the ratio of nPR isoforms [35]. It is essential to uncover the key mediators of MIF’s anti-tumor activity and its relationship to mPRs [59]. Our findings of altered expression patterns of all three CCM proteins in nPR( +) breast cancer cells, under mPR-specific PRG actions (MIF alone or PGR + MIF), strongly suggests the involvement of the CSC during Luminal-A breast cancer tumorigenesis and identified that MIF’s anti-tumor activity is likely realized through mPRs. Furthermore, utilizing high throughput omic approaches, we have identified significant alterations to key players in P53 signaling, P13K-AKT signaling, cell cycle, apoptosis, WNT, MAPK, and various pathways in cancer as well as steroid hormone biosynthesis signaling pathways under mPR-specific PRG actions or disrupted CSC conditions. These results solidify our discovery of the novel CmPn signaling network which is dynamically modulated and fine-tuned with a series of feedback regulations in nPR( +) breast cancer cells.

Using systems biology approaches, we preliminarily discovered a total of 9 Luminal-A intrinsic, 2 PRG-inducible, 1 PRG-repressive, 1 mPR-specific PRG-repressive, and 2 mPR-responsive candidate biomarkers with altered gene expression, under a disrupted CmPn signaling network in Luminal-A breast cancers. Interestingly, down-regulated gene expression was observed among all treatment conditions, but up-regulated genes were only observed in hormone treatments, suggesting that a disrupted CSC suppresses the expression of up-regulated CmPn players observed under mPR-specific PRG actions. After performing EMT score signature analysis on our identified biomarkers, we observed that these candidate biomarkers are most likely not major drivers for EMT transition during tumorigenesis. Furthermore, by integrating gene expression and clinical data simultaneously, we were able to obtain significant KM survival curves for 9 of our biomarkers using Luminal-A breast cancer tissue data, and 10 biomarkers using normal breast cancer tissue data. Together, combining our systems biology analysis with a dual validation approach, we have confirmed/validated only two intrinsic biomarkers, *RPL13* and *RPL38*, for Luminal-A breast cancers. Finally, this work reaffirms our previous notions of the heterogeneity among Luminal-A breast cancers in response to sterol actions and illustrates the essential role of the CmPn signaling network during breast cancer tumorigenesis. Furthermore, our works here demonstrate that disruption of the CmPn network in Luminal-A breast cancers could lead to undesired clinical outcomes.

## Conclusion

Despite its significance, the relationship between classic and non-classic PRG receptors has been drastically unexplored. It has been reported that activation of mPRs leads to activation of nPRs, leading to an integrated model where steroid hormone-dependent mPRs contribute to later nuclear nPR actions [62]. In this study, we provide strong evidence that the CSC plays an essential role to bridge crosstalk among nPRs, mPRs, and their shared ligands (PRG and MIF) to form the CmPn signaling network to modulate this cascade among nPR( +) Luminal-A breast cancer cells. The possible convergence of classic, non-classic PRG actions and CSC signaling on their common cellular targets, in nPR( +) breast cancer cells is an attractive model by which PRG or MIF can fine-tune this intricate balance. This also raises the possibility that PRG or MIF may intervene in different signaling pathways depending on the cellular context. Disruption of this intricate balance, including patients under HRT or females taking hormonal contraceptives during their reproductive ages, could result in perturbation of the CmPn signaling network with potential consequences of increased risks of breast cancer or compromised tumor therapy.

## Supplementary Information

**Additional file 1:** Supplemental materials.

## Data Availability

The datasets used and/or analyzed during the current study are available from the corresponding author on reasonable request. Additionally, all necessary data generated or analyzed during this study are included in this published article [and its Additional file 1].

## Abbreviations

Breast cancer: Breast Cancer
CCM: Cerebral cavernous malformation
CSC: CCM signaling complex
PRG: Progesterone
MIF: Mifepristone
mPRs: Membrane progesterone receptors
nPRs: Nuclear Progesterone Receptors
PAQRs: Progestin and AdipoQ Receptors
PAQR7: MPRα
PAQR8: MPRβ
PAQR5: MPRγ
PAQR9: MPRε
PGRMC1: Progesterone receptor membrane component 1
CmPn: CSC-mPRs-PRG-nPRs
DEGs: Differentially expressed genes
DEPs: Differentially expressed proteins
