# Supplementary material for: CCM signaling complex (CSC) couples both classic and non-classic Progesterone receptor signaling
Source: Cell Commun Signal. 2022 Aug 15;20:120. doi: 10.1186/s12964-022-00926-z (PMC9377144; doi:10.1186/s12964-022-00926-z)
Supplement: Supplementary file 2 — Additional file 1: Supplemental materials. [file 12964_2022_926_MOESM2_ESM.pdf]

Supplementary Materials for

CCM signaling complex (CSC) couples both classic and non-classic progesterone receptor signaling

Johnathan Abou-Fadel[1, †], Xiaoting Jiang[1, †], Brian Grajeda[2], Akhil Padarti[1],Cameron C. Ellis[2], Esmeralda Flores[1], Alyssa-Marie D. Cailing-De La O[1], Jun Zhang[1*]

[1]Department of Molecular and Translational Medicine (MTM)
Texas Tech University Health Science Center El Paso, El Paso, TX 79905 USA
[2]Department of Biological Sciences
University of Texas at El Paso, El Paso, TX 79902 USA

Correspondence to: jun.zhang2000@gmail.com

This PDF file includes:

Supplementary Text
Supplementary Methods
Supplementary Figures 1-12 legends
Supplementary Tables 1-3 legends

Other Supplementary Materials for this manuscript include the following:

N/A

Supplementary Text

We have done our best to number Supplemental Figures/tables to follow along with the numbering of figures in the main text to aid in readability.

Supplementary Methodology

Cell migration assay: We calculated the rate of cell migration (R_M) with the following equation: RM=(Wi-Wf)/t; W_i is the average of the initial wound width (µm), W_f is the average of the final wound width (µm) and t is the time span (hrs) of the assay.

Wound healing assay: The wound areas were visualized using a Nikon Biostation and recorded with a high-resolution digital camera. Wound closure was quantified temporally by measuring the average width of the wounds using a Nikon Biostation. The wound area was measured temporally from five different fields under 40× magnifications for each condition and cell type. Each experiment was repeated three times. The wound area, wound coverage of total area, as well as average and standard deviation of the scratch width was performed with the aid of an imageJ plugin [1]. We calculated the percentage of wound closure using the following equation: Wound Closure % = ($A_{t=0}$-$A_{t=\Delta t}$/At=0)*100; $A_{t=0}$ is the initial wound area, $A_{t=\Delta t}$ is the wound area after n hours of the initial scratch, both in μm^2.

Cell Invasion assay. The migration of T47D and MCF7 cells were performed using a 24-well transwell chamber (8 µm pore size; Millipore, Billerica, MA, USA). The cells were plated in triplicates into the upper chamber (1×10^5 cells/well) with 1640 (no FBS) in the presence of combined steroids (MIF+PRG, 20 µM each) or vehicle alone for 48 hrs at 37°C and 5% CO_2 conditions. The bottom chambers were supplemented with 1640

containing 10% FBS. Non-invading cells remaining on the upper surface of the membrane were removed using a cotton swab, and the invading cells on the bottom surface were stained with Diff-Quick stain kit (Fisher). Invading cells were visualized using 20X (T47D cells) or 40X (MCF7 cells) magnification on a Nikon microscope and photographed with a high-resolution digital camera. The invasion value was quantified from the mean number of five different fields per condition. Each experiment was repeated three times.

Immunohistochemistry (IHC) and immunofluorescence (IF)

Deparaffinization of paraffin-embedded tissue sections for IHC: Breast cancer tissue slides were purchased from various suppliers (BioChain and US Biomax) and were baked at 60°C for 2 hrs. Once cooled, the sections were washed 3X in xylene for 5 min each, followed by 3-min sequential washes in 100, 95, 90, 80 and 70% ethanol and then soaked in water before an antigen retrieval step.

Growth of T47D cells using chamber slides for IF applications: IF staining methods were performed as previously described [2]. Briefly, cells were grown to confluency and then treated with MIF+PRG (20 µM each) over time in Glass chamber Slides (Nunc-Lab-Tek II) and fixed using 4% (*w/v*) Paraformaldehyde (PFA). Slides were washed 3X with PBT (0.2% Triton X-100) before proceeding with antigen retrieval step.

Antigen Retrieval for IHC/IF: Slides were submerged in 10mM sodium citrate buffer ($Na_3C_6H_5O_7$, pH 6.0) containing 0.01% Triton X-100 at 95-98°C. Slides were kept in buffer at 95-98°C for 30 mins and then allowed to cool down to room temperature (RT).

Blocking and Antibody incubation for horseradish peroxidase (HRP)/3,3'-diaminobenzidine (DAB) detection system (IHC): Slides were processed according to

manufacturer's protocol supplied with the AB-64261 HRP/DAB detection kit (Abcam). Primary antibodies used are detailed in suppl. table 2.

Counterstaining with hematoxylin and mounting/sealing for IHC: Cell nuclei were counterstained using Harris Hematoxylin solution (Sigma-Aldrich; Merck KGaA) following manufacturers protocol. The tissue was then mounted using xylene-based mounting media and allowed to sit for 1 hr, before sealing with nail polish to cure O/N.

Blocking and Antibody incubation for IF: After antigen retrieval, slides were briefly incubated in PBS containing 0.2% Triton X-100 for 10 mins at RT to permeabilize cells. Following permeabilization, cells were blocked with Pierce fast blocking buffer (Fisher) for 2hrs at RT, followed by antibody incubation (Suppl. table 2).

Nuclear staining and Mounting/Sealing for IF: Immunofluorescence slides are DAPI stained during the mounting/sealing process (Suppl. Table 2). To allow efficient staining of DAPI, slides should be allowed to rest O/N at $4^{o}C$ before sealing to cure O/N.

Imaging and Quantification for IF: Imaging was performed using a Nikon Eclipse Ti confocal microscope using a 60X objective lens as previously described [3]. Quantification was done automatically using Elements Analysis software provided with the Nikon microscope. Thresholding (used for quantification) was defined and maintained throughout all images for each application to ensure no bias was applied to data and to exclude low and high outliers. Using Nikon elements analysis tools, localization of CCM1/CCM3/PAQR8 was performed using binary operations to identify the relative ratio of expressed proteins that localized inside the nucleus (overlaps of DAPI/GFP or

mCherry) compared to expressed proteins that localized in the cytosol (unique GFP or mCherry without DAPI signal). Fluorescent images are quantified for CCM1 using 488nm wavelength while CCM3 and PAQR8 were quantified using 555nm wavelength channel.

Imaging and Quantification for IHC: Imaging was carried out using a Nikon EclipseTi microscope with a color camera and a 10X objective lens. Quantification was conducted automatically using Elements Analysis software. Threshold was defined and maintained throughout all images for each application to ensure no bias was applied to the data. Thresholds were applied to exclude low and high outliers. The red/brown color from the HRP/DAB reactivity with CCM2/PAQR7 antibodies was quantified and averaged between the red and green channel quantification.

Supplementary References

1. Suarez-Arnedo A, Torres Figueroa F, Clavijo C, Arbelaez P, Cruz JC, Munoz-Camargo C: **An image J plugin for the high throughput image analysis of in vitro scratch wound healing assays**. *PLoS One* 2020, **15**(7):e0232565.
2. Abou-Fadel J, Qu Y, Gonzalez E, Smith M, Zhang J: **Emerging roles of CCM genes during tumorigenesis with potential application as novel biomarkers across major types of cancers**. *ONCOLOGY REPORTS* 2020.
3. Abou-Fadel J, Jiang X, Grajeda B, Padarti A, Ellis CC, Zhang J: **CCM signaling complex (CSC) coupling both classic and non-classic progesterone receptor signaling** *bioRxiv* 2020.

Supplementary Legends

Suppl. Table 1. Small double-stranded interfering RNAs (siRNA) used for *gene silencing* in RNA interference (RNAi) experiments. Gene-specific *knockdown* was achieved by introducing small double-stranded siRNA oligos. Detailed information about application, siRNA oligo duplexes, manufacturers, and category numbers are listed.

Suppl. Table 2. Antibodies used in this study. Detailed information about application, antigen, clone code, secondary antibody, manufacturer, and category number are listed. * IHC, Immunohistochemistry; IF, immunofluorescence; DAB, HRP/DAB staining, WB, Western Blots.

Suppl. Table 3. Primers for quantitative PCR (qPCR) for the detection of differentially expressed targeted genes. The sequence, qPCR product size and optimized annealing temperature of each primer pairs are listed.

Suppl. Fig. 1. Up-regulation of CCM and mPR proteins in breast cancers. Relative expression of total CCM proteins in paired-tissue breast cancer samples **A**). Immunohistochemistry (IHC) approaches utilizing Horse Radish Peroxidase (HRP)/ 3,3′-Diaminobenzidine (DAB) staining revealed an increase in the relative intensity of CCM2 staining between representative breast tumor tissues compared to normal breast tissues (left panel); The corresponding, representative quantification is presented (middle panel) with statistical significance from the entire collection of paired samples (n=11, right panel) **B**). Significant increased protein expressions of CCM1 and CCM3 were visualized (left panel) in breast tumor (T), compared to normal tissue (N) samples using immunofluorescence (IF) imaging (n=3). **C)**. Significant increased expression of PAQR7 was observed with HRP/DAB staining in breast tumors (Breast Carcinoma) from a selected set of breast tissue-pairs (left panel) with representative quantification displayed (middle panel). Statistically significant increased expression of PAQR7 (mPRα) was found in tumor tissues from the entire collection of paired samples (right panel, n=10). For HRP/DAB staining (panels A and C), the Red/Brown color from HRP/DAB reactivity is quantified and averaged between the red and green channel quantification. In fluorescent staining experiments (panel B), CCM1 and CCM3 were quantified through ROI intensities using wavelength channels 488 and 647nm, respectively. Data for both microscopy approaches were normalized against its respective internal controls using the blue channel for cell nuclei (HRP/DAB) or 408nm wavelength for DAPI (fluorescent) and background staining. For each section pair, Region of Interest (*ROI*) i*ntensities* were automatically quantified (over 1000 times/per section). All data from entire collections (n>10) were normalized by normal tissue among each tissue pair. All imaging data were

acquired using a Nikon EclipseTi confocal microscope and quantified with Elements Analysis software (Nikon). In all bar plots, red line is the control baseline for fold change measurements (-/+) and *** above any bar graphs indicate P ≤ 0.001 for un-paired *t*-test. In HRP/DAB staining, the Red/Brown color from HRP/DAB reactivity with a specific antibody is quantified and averaged between the red and green channel quantification and cell nuclei are quantified with the blue channel. Data were normalized against its respective control using the blue channel for cell nuclei and background staining as described earlier and quantified with Nikon Elements Analysis software.

Suppl. Fig. 2. Protein expression levels of the CCM Signaling Complex (CSC) in various breast cancer cell lines. *Subtypes* of breast cancer cell lines (MDA-MB-231, MCF7, MDA-MB-468, BT474, T47D, MDA-MB-453) and prostate cancer cell lines (PC3, and C4-2) were utilized to screen the expression patterns of CCM2 protein isoforms (A-I). T47D cells, which displays expression of all CCM2 isoforms, had the highest expression levels of the three most abundant isoforms, T1, T2 and T3, along with PC3 prostate cancer cell line. Protein expression and composition of CCM2 isoforms do not correlate with either cellular immune-profile or molecular subtypes (bottom panel).

Suppl. Fig. 3. Expression levels of membrane progesterone receptors (mPRs/PAQRs) are modulated by progesterone (PRG) and mifepristone (MIF) and the CSC in Luminal-A T47D cells. A. *Protein expression levels of major mPRs (PAQRs, PGRMC1) are not influenced by silencing either androgen receptor (AR), glucocorticoid receptor (GR) or classic progesterone receptor (nPRs) genes.* After silencing AR, GR, and nPR genes for 48 hrs, no significant changes in protein expression levels of mPRs (PAQRs, PGRMC1) in T47D cells were observed. **B.** *RNA expression levels of major*

mPRs (PAQR7/8/9) are not influenced by silencing nPR genes. After silencing *nPRs* genes for 48 hrs, no significant change in RNA expression levels of three major mPRs (PAQR7/8/9) were observed. Relative RNA expression changes of mPRs (*PAQR7/8/9*) in T47D cells were measured by qPCR in (Fold changes) (triplicates per experiment, n=3).

Suppl. Fig. 4. CCM2 is a cornerstone for the stability of the CSC. *Knockout of CCM2 decreases the expression levels of CCM1/3 proteins in zebrafish embryos.* **A)**. Significant decreased expression of both CCM1/3 proteins were observed in *CCM2*-knockout (KO) zebrafish embryos (*vtn*) (Left upper and lower panels). The relative expression levels of CCMs (1, 2, 3) proteins were measured through quantification of band intensities and normalized against α-actinin (ACTN1) followed by WT strains (red lines, right panel, n=3). **B)**. Significant increased levels of *CCM2* isoforms in *CCM1*-KO zebrafish (*san*) were observed. The relative RNA expression changes of *CCM1* and 4 isoforms of *CCM2* in *CCM1*-KO and *CCM2*-KO zebrafish (*san, vtn,* respectively) were measured by RT-qPCR (Fold, n=3). Primer information for zebrafish RT-qPCR can be provided upon request. **C.** In Figure 4, Relative RNA expression changes were measured by qPCR (Fold changes) and normalized to scramble control (red line, triplicates per experiment, n=3). Relative expression levels of proteins were measured through quantification of band intensities and normalized against either α-actinin (ACTN1) or β-actin (ACTB) followed by SC controls (red line). In all bar plots, red line is the control baseline for fold change measurements (-/+). **, *** above bar indicates P ≤ 0.01 or 0.001 for paired t- test, respectively.

Suppl. Fig. 5: Temporal RNA expression patterns of androgen receptor (AR) and glucocorticoid receptor (GR) under mPR-specific PRG actions in Luminal-A T47D and MCF7 cells: Inducible RNA expression of *GR* under mPR-specific PRG actions in both nPR(+) breast cancer cells. T47D and MCF7 cells were treated with mPR-specific PRG treatment (PRG+MIF, 20 µM each) for 72 hrs. We observed that while no induced RNA expression of *AR* was observed, significant inducible RNA expression of *GR* in MCF7 was observed, under mPR-specific PRG actions (bottom panel). Surprisingly, RNA expression of *GR* was similarly induced in nPR(+) T47D cells (top panel), which physiologically has almost un-detectable *GR* levels. Relative RNA expression levels of *AR* and *GR* in T47D and MCF7 cells were measured by RT-qPCR (Fold) and normalized against vehicle controls (red line, n=4).

Suppl. Fig. 6. Modulation of key factors of the CmPn network in Luminal-A breast cancer cells under mPR-specific PRG actions. T47D cells were treated with mPR-specific PRG treatment (MIF+PRG, 20µM each) for 72 hrs. **A-1**. *Relative expression of* CCM1 *protein.* IF approaches revealed decreased expression in the relative intensity of CCM1 staining in T47D cells treated with MIF+PRG for 72 hrs. Additionally, the majority of CCM1 resides in the cytoplasm with no drastic changes in localization of CCM1 proteins in and out of the nucleus between 0 hrs and 72 hrs. **A-2**. *Relative expression of CCM3 protein.* IF approaches revealed decreased expression in the relative intensity of CCM3 staining in T47D cells treated with mPR-specific PRG actions for 72 hrs. Additionally, the majority of CCM3 resides in the cytoplasm with no drastic changes in localization of CCM3. **A-3**. *Relative expression of PAQR8 protein*. IF approaches revealed modulation in the localization of PAQR8 in which T47D cells demonstrated more

PAQR8 staining inside the nucleus at 72 hrs compared to 0 hrs. **B-1**. *Temporal modulation of CCM1 protein in T47D cells.* T47D cells were treated with mPR-specific PRG treatment for 0-72 hrs and IF approaches revealed significant decreased expression observed after 4 hours in the relative intensity of CCM1 staining in T47D cells treated with mPR-specific PRG actions. **B-2.** *Temporal modulation of CCM3 protein in T47D cells.* IF approaches revealed decreased expression observed after 24 hours in the relative intensity of CCM3 staining in T47D cells treated with mPR-specific PRG actions. **B-3.** *Temporal modulation of PAQR8 (P8) protein in T47D cells.* IF approaches revealed increased expression observed at 24hrs in the relative intensity of PAQR8 staining in T47D cells treated with mPR-specific PRG actions.

Suppl. Fig. 7. Differentially expressed gene (DEG) detection utilizing RNAseq. A-B. Cluster software and Euclidean distance matrixes were used for the hierarchical clustering analysis of the expressed genes (RNA) and sample program at the same time to generate the displayed Heatmap of hierarchical clustering for the intersection of DEGs *(A)* or union of DEGs *(B)* of expression clustering scheme; x axis represents each comparing sample and Y axis represents DEGs. Coloring indicates the log2 transformed fold change (high: red, low: blue). **C-D.** Volcano plot of DEGs for MIF treated *(C)* or mPR-specific PRG treated cells *(D)*; X axis represents log2 transformed fold change. Y axis represents -log10 transformed False Discovery Rate (FDR). . Red points represent up-regulated DEGs. Blue points represent down-regulated DEGs. Gray points represent non-DEGs. **E-F***.* Pathway functional enrichment result for up/down regulation of genes with MIF treatment *(E)* or mPR-specific PRG treatment *(F).* X axis represents the terms of

Pathway. Y axis represents the number of up/down regulated genes. Raw RNAseq data can be provided upon request.

Suppl. Fig. 8. Differentially expressed Proteins (DEPs) detection utilizing Liquid Chromatography-Tandem Mass Spectrometry (LC-MS/MS). A-C. Cluster software and Euclidean distance matrixes were used for the hierarchical clustering analysis of the expressed proteins and sample program at the same time to generate the displayed Heatmap of hierarchical clustering for DEPs for T47D cells under MIF-only treatment *(A)* mPR-specific PRG treatment *(B)* and our disrupted CSC model (C); x axis represents each comparing sample and Y axis represents DEPs. Coloring indicates the log2 transformed fold change (high: red, low: blue). **D-F**. Enhanced volcano plot of DEPs for T47D cells under MIF-only treatment *(D)* mPR-specific PRG treatment *(E)* and our disrupted CSC model (F); X axis represents log2 transformed fold change. Y axis represents -log10 transformed significance. **G-I**. Pathway functional enrichment result for up/down regulation of genes for T47D cells under MIF-only treatment *(G)* mPR-specific PRG treatment *(H)* and our disrupted CSC model (I). X axis represents the terms of Pathway. Y axis represents the number of DEPs. Raw proteomics data can be provided upon request.

Suppl. Fig. 9: Differential expression of candidate biomarkers for breast cancer tissues. Microarray data containing breast cancer tumors, analyzed using kmplot software, were divided into two groups based on nuclear PRG receptor (nPR) status determined by Immunohistochemistry (IHC); after filtering, there were 925 nPR(-) and 926 nPR(+) breast cancer samples. **A.** Genes with significant decreased expression in nPR(+)

breast cancer tissue samples compared to nPR(-) tissues. **B**. Genes with significant increased expression in nPR(+) breast cancer tissue samples compared to nPR(-) tissues. Statistical significance was performed with students *t*-test where *, **, *** above bar indicates P ≤ 0.05, 0.01 or 0.001, respectively.

Suppl. Fig. 10: RNAseq expression profiling for candidate biomarkers for Luminal-like breast cancer tissues using TCGA data. We evaluated our candidate biomarkers expression data using the TCGA database for breast cancer tissues based on PAM50 classification. **A)** *Expression profiles among all 3 luminal-like breast cancers (normal-like, Luminal-A and Luminal-B).* Our analysis confirmed significant differential expression patterns for almost all biomarkers (with the exception of *VAPA*), assessed using One-way ANOVA, among all 3 luminal-like breast cancers. **B)** *Expression profiles among normal and Luminal-A breast cancers.* Similar to panel A, our analysis confirmed significant differential expression patterns for almost all biomarkers (again with the exception of *VAPA*), between normal and luminal-A breast cancers. **C)** *Expression profiles among normal and Luminal-B breast cancers.* Our analysis confirmed significant differential expression patterns for 12/15 biomarkers (no significance for *RPL38, SNX2*, and *VAPA*), between normal and luminal-B breast cancers. **D)** *Expression profiles among Luminal-A/B breast cancers.* Our analysis confirmed significant differential expression patterns for 8/15 biomarkers (no significance for CLUH, RANBP2, STK24, ANP32A, *EFHD1, SNX2*, and *VAPA*), between luminal-A and luminal-B breast cancers. For all graphs, X axis details genes profiled, while Y axis details Log2 batch-effect normalized RNAseq expression data. All graphs were produced using the Xena platform. **For legends in Figure 1 panels D-F: Panel D:** Legend: T1: tumor≤2cm across; T1b: tumor>0.5cm≤1cm;

T1c: tumor>1cm≤2cm; T2: tumor>2cm≤5cm; T3: tumor>5cm across; T4: tumor>5cm across and has additional infiltrating characteristics; T4b: tumor has T4 characteristics and additionally has grown into the skin. **Panel E:** Legend: N0: No regional lymph node metastasis identified or isolated tumor cells (ITCs) only; N0(I-): No regional lymph node metastases histologically, negative IHC; N0(I+): ITCs only (malignant cell clusters ≤0.2 mm) in regional lymph node; N1: Micrometastases or metastases in 1–3 axillary lymph nodes and/or clinically negative internal mammary nodes with Micrometastases or macrometastases by sentinel lymph node biopsy; N1a: Metastases in 1–3 axillary lymph nodes, at least one metastasis >2.0 mm; N1b: Metastases in ipsilateral internal mammary sentinel nodes, excluding ITCs; N1m1: Micrometastases (~200 cells, >0.2 mm, but ≤2.0 mm); N2: Metastases in 4–9 axillary lymph nodes or positive ipsilateral internal mammary lymph nodes by imaging in the absence of axillary lymph node metastases; N2a: Metastases in 4–9 axillary lymph nodes (at least 1 tumor deposit >2.0 mm); N3: Metastases in ≥10 axillary lymph nodes; N3a: Metastases in ≥10 axillary lymph nodes (at least 1 tumor deposit >2.0 mm). **Panel F:** Legend: M0: there is no sign that the cancer has spread; cM0(I+): There is no sign of the cancer on physical examination, scans or x-rays, but cancer cells are present in blood, bone marrow, or lymph nodes far away from the breast cancer; M1: Cancer measuring more than 0.2 mm across has spread to another part of the body and confirmed by examining tissue from a biopsy, or surgery and scans.

Suppl. Fig. 11: Prognostic effects for identified candidate biomarkers utilizing microarray data of 631/1809 Luminal-A breast cancer patients. Publicly available

microarray data (22,277 probes) from 631/1,809 breast cancer patients (depending on probe) was analyzed using Kaplan-Meier plotter to integrate gene expression and clinical data simultaneously to generate the displayed Kaplan-Meier (KM) survival curves. Breast cancer patients were filtered to only analyze patient samples classified as ER(+)/PR(+)/HER2(-)/Luminal-A breast cancer subtype (identical to T47D cells). Results demonstrated significantly worst prognosis with increased expression of **(1)** *MCM6*, **(2)** *ANP32A*, **(3)** *TRIP13*, and decreased expression of **(4)** *LPP*, **(5)** *RPL38*, **(6)** *RPL13*, **(7)** *KIAA0664* (*CLUH*), **(8)** *SNX2* and **(9)** *VAPA*. Logrank P-values are calculated and displayed as well as hazard ratio (and 95% confidence intervals). Red line demonstrates high gene expression, while black line demonstrates low gene expression.

Suppl. Fig. 12: Prognostic effects for identified candidate biomarkers utilizing microarray data of 35/119 Normal breast cancer patients. Publicly available microarray data (22,277 probes) from 35/119 breast cancer patients (depending on probe) was analyzed using Kaplan-Meier plotter to integrate gene expression and clinical data simultaneously to generate the displayed KM survival curves. Breast cancer patients were filtered to only analyze patient samples classified as ER(+)/PR(+)/HER2(-)/Normal breast cancer subtype (identical receptors' expression as Luminal-A). Results demonstrated significantly worst prognosis with decreased expression of **(1)** *RPL13*, **(2)** *VAPA*, **(3)** *STK24*, **(4)** *RPL38*, **(5)** *TRIP13*, **(6)** *EFHD1*, and increased expression of **(7)** *MCM6*, **(8)** *RANBP2*, **(9)** *ANP32A*, and **(10)** *LPP*. Logrank P-values are calculated and displayed as well as hazard ratio (and 95% confidence intervals). Red line demonstrates high gene expression, while black line demonstrates low gene expression.

Suppl. Fig. 13: Original digital images at a lower exposure of Western blots in Figures 3 (A, B, D, G), 4 (B, C, G), and 5 (A). Protein lysates were separated by SDS-PAGE using Criterion Precast gels (Bio-Rad), with TGX gels, 4-15% Tris-HCL gels and 16.5% Tris-Tricine gels, respectively.

Suppl. Fig. 14: Original digital images at a lower exposure of Western blots in Suppl. Figures 2, 3 (A), and 4 (A, B). Protein lysates were separated by SDS-PAGE using Criterion Precast gels (Bio-Rad), with TGX gels, 4-15% Tris-HCL gels and 16.5% Tris-Tricine gels, respectively.

Suppl. Table 1

Application	siRNAs	Target	Vendor	Cat #
RNAi	ON-TARGETplus Human KRIT1 siRNA - SMARTpool	Krit1	Dharmacon	L-003825-00-0010
RNAi	ON-TARGETplus Human CCM2 siRNA - SMARTpool	MGC4607	Dharmacon	L-014728-01-0010
RNAi	ON-TARGETplus Human CCM2l siRNA - SMARTpool	MGC4607l	Dharmacon	L-018652-02-0010
RNAi	ON-TARGETplus Human PDCD10 siRNA - SMARTpool	PDCD10	Dharmacon	L-004436-00-0010
RNAi	KRIT1 (Human) - 3 unique 27mer siRNA duplexes	Krit1	OriGene	SR313185
RNAi	CCM2 (Human) - 3 unique 27mer siRNA duplexes	MGC4607	OriGene	SAB2500214
RNAi	PDCD10 (Human) - 3 unique 27mer siRNA duplexes	PDCD10	OriGene	sc-365586
RNAi	Ccm2 Rat siRNA Oligo Duplex (Locus ID 305505)	MGC4607	OriGene	SR509691
RNAi	ON-TARGETplus Human PAQR7 (164091) siRNA - SMARTpool	PAQR7	Dharmacon	L-008033-00- 0005
RNAi	ON-TARGETplus Human PAQR8 (85315) siRNA - SMARTpool	PAQR8	Dharmacon	L-007820-00- 0005
RNAi	ON-TARGETplus Human PAQR5 (54852) siRNA - SMARTpool	PAQR5	Dharmacon	L-008034-00- 0005
RNAi	ON-TARGETplus Human PAQR6 (79957) siRNA - SMARTpool	PAQR6	Dharmacon	L-008054-020005
RNAi	ON-TARGETplus Human PGRMC1 (10857) siRNA - SMARTpool	PAQR9	Dharmacon	L-010642-000005
RNAi	ON-TARGETplus Human PGR (5241) siRNA - SMARTpool	PR1/2	Dharmacon	L-003433-000005
RNAi	ON-TARGETplus Human AR (367) siRNA - SMARTpool	AR	Dharmacon	L-003400-000005
RNAi	ON-TARGETplus Human NR3C1 (2908) siRNA - SMARTpool	GR	Dharmacon	L-003424-000005

Suppl. Table 2

Application	Antigen	Gene	Clone	Secondary	Vendor	Cat #
IF/WB	CCM1	Krit1	E-8	(anti-mouse)	Santa Cruz	sc-514371
IF/WB	CCM1-AF488	Krit1	E-8	(anti-mouse)	Santa Cruz	sc-514371
IF/WB	CCM1	Krit1	8-RY2	(anti-mouse)	Santa Cruz	sc-134376
IF/WB	CCM1	Krit1		(anti-rabbit)	OriGene	AP26021PU-L
IHC/DAB/WB	CCM2	MGC4607		(anti-rabbit)	Novus	NBP1-86730
IHC/DAB/WB	CCM2	MGC4607		(anti-rabbit)	Novus	NBP215761
IHC/DAB/WB	CCM2	MGC4607		(anti-goat)	Sigma	SAB2500214
IF/WB	CCM3	PDCD10	C-8	(anti-mouse)	Santa Cruz	sc-365586
IF/WB	CCM3-AF647	PDCD10	C-8	(anti-mouse)	Santa Cruz	sc-365586
IF/WB	CCM3	PDCD10	F-12	(anti-mouse)	Santa Cruz	sc-365587
WB	PAQR5	mPRγ		(Anti-Rabbit)	Aviva	OASG04642
WB	PAQR6	mPRδ		(Anti-Rabbit)	Aviva	ARP49900
WB	PAQR6	mPRδ		(Anti-Rabbit)	Aviva	ARP49901
WB	PAQR5/6	mPRδ/γ	B-8	(anti-mouse)	Santa Cruz	sc-514273
WB	PAQR7	mPRα		(Anti-Rabbit)	Aviva	OASG04641
WB	PAQR7	mPRα		(Anti-Rabbit)	Aviva	ARP67727
WB	PAQR8	mPRβ		(Anti-Rabbit)	Aviva	ARP66903
WB	PAQR9	mPRε		(Anti-Rabbit)	Aviva	ARP62890
WB	PGRMC1		C-4	(anti-mouse)	Santa Cruz	sc-393015
WB	PR		AB52	(anti-mouse)	Santa Cruz	sc-810
WB	PR		F-4	(anti-mouse)	Santa Cruz	sc-166169
WB	AR		441	(anti-mouse)	Santa Cruz	sc-7305
WB	GR		FiGR	(anti-mouse)	Santa Cruz	sc-12763
WB	GR		3D5	(anti-mouse)	Santa Cruz	sc-56851
WB	β-actin		C-2	(anti-mouse)	Santa Cruz	sc-8432
WB	α-actinin		H-2	(anti-mouse)	Santa Cruz	sc-17829
WB	α-actinin-AF488		H-2		Santa Cruz	sc-17829
WB	Rabbit-HRP				Santa Cruz	sc-2357
WB	Mouse-HRP		m-IgGκ BP		Santa Cruz	sc-516102
WB	m-IgGκ BP IgG-CFL 488		m-IgGκ BP		Santa Cruz	sc-516176
WB	Rabbit IgG-CFL 488				Santa Cruz	sc-516248
IHC/IF	DAPI				Santa Cruz	sc-3598
IF	PAQR8	mPRβ		(Anti-Rabbit)	Aviva	OAAB11180
IF	mouse anti-rabbit IgG-CFL 488			secondary	Santa Cruz	sc-516248
IF	mouse anti-rabbit IgG-CFL 555			secondary	Santa Cruz	sc-516249

Suppl. Table 3

Target Gene	Primer	Sequence	Product Size (bp)	Anneling Temp (℃)
**PR1/2**	PR-A/B-F1	CGCGCTCTACCCTGCACTC	121	65
	PR-A/B-R1	TGAATCCGGCCTCAGGTAGTT		
**mPRα/PAQR7**	PAQR7-F1	CGCTCTTCTGGAAGCCGTACATCTATG	122	65
	PAQR7-R1	CAGCAGGTGGGTCCAGACATTCAC		
**mPRβ/PAQR8**	PAQR8-F1	AGCCTCCTACATAGATGCTGCCC	194	65
	PAQR8-R1	GGTGCCTGGTTCACATGTTCTTCA		
**mPRγ/PAQR5**	PAQR5-F1	CAGCTGTTTCACGTGTGTGTGATCCTG	144	65
	PAQR5-R1	GCACAGAAGTATGGCTCCAGCTATCTGAG		
**mPRδ/PAQR6**	PAQR6-F2	GTTGACCCACCAGCTTAGGA	176	65
	PAQR6-R2	ATGCCATCTTCCCAGAACAC		
**mPRε/PAQR9**	PAQR9-F1	TGCTACAAAGGGATCCCAAC	202	65
	PAQR9-R1	TGGCACAGATGATTGGAAAA		
**PGRMC1**	PGRMC1-F1	CTGCATGATTTCTGTTTTATCTACCTCTA	86	65
	PGRMC1-R1	TGTTACTGGACAGCGCTTAATCC		
**AR**	AR-F1	CCTGGCTTCCGCAACTTACAC	168	65
	AR-R1	GGACTTGTGCATGCGGTACTCA		
**GR/GCR**	GR-F1	TCAAAAGAGCAGTGGAAGG	260	65
	GR-R1	GGTAGGGGTGAGTTGTGGTAACG		

Suppl. Fig. 1A

Normal Breast Tissue

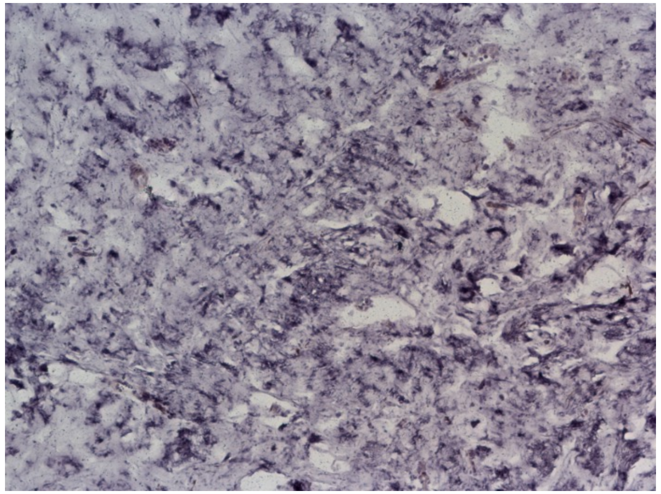

Breast Carcinoma

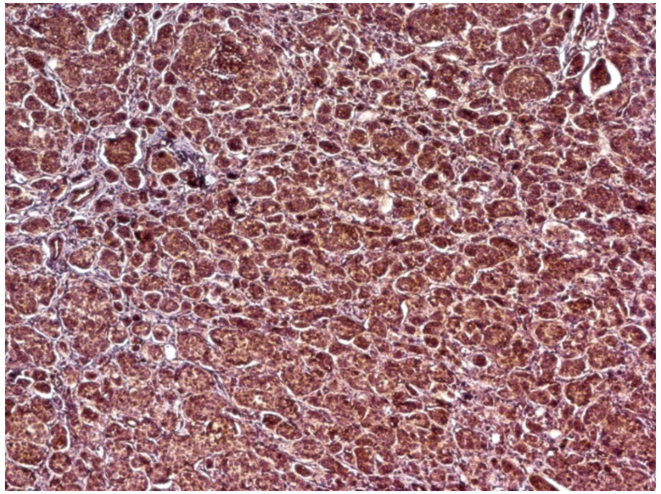

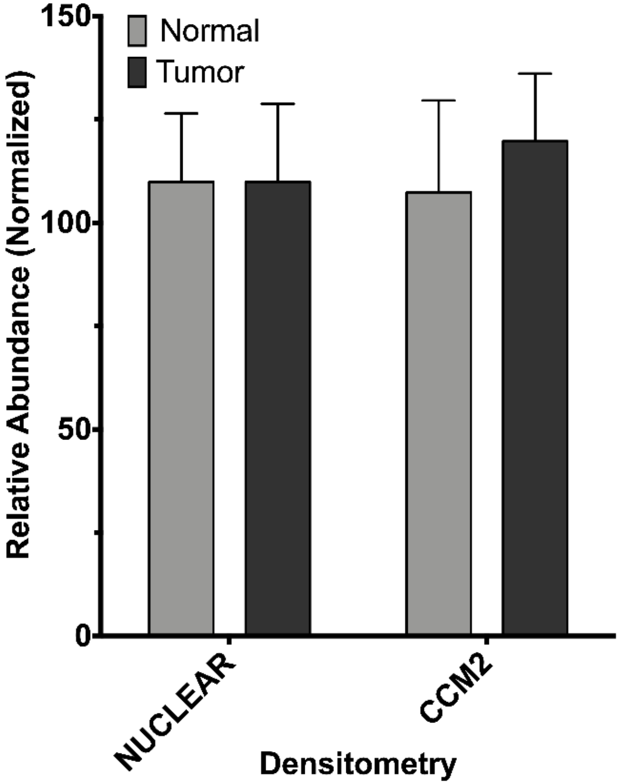

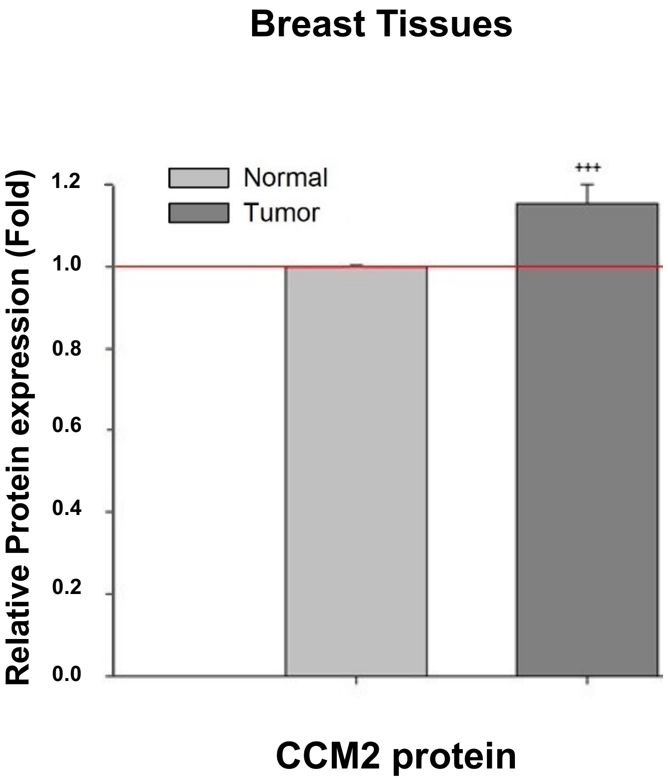

S1B

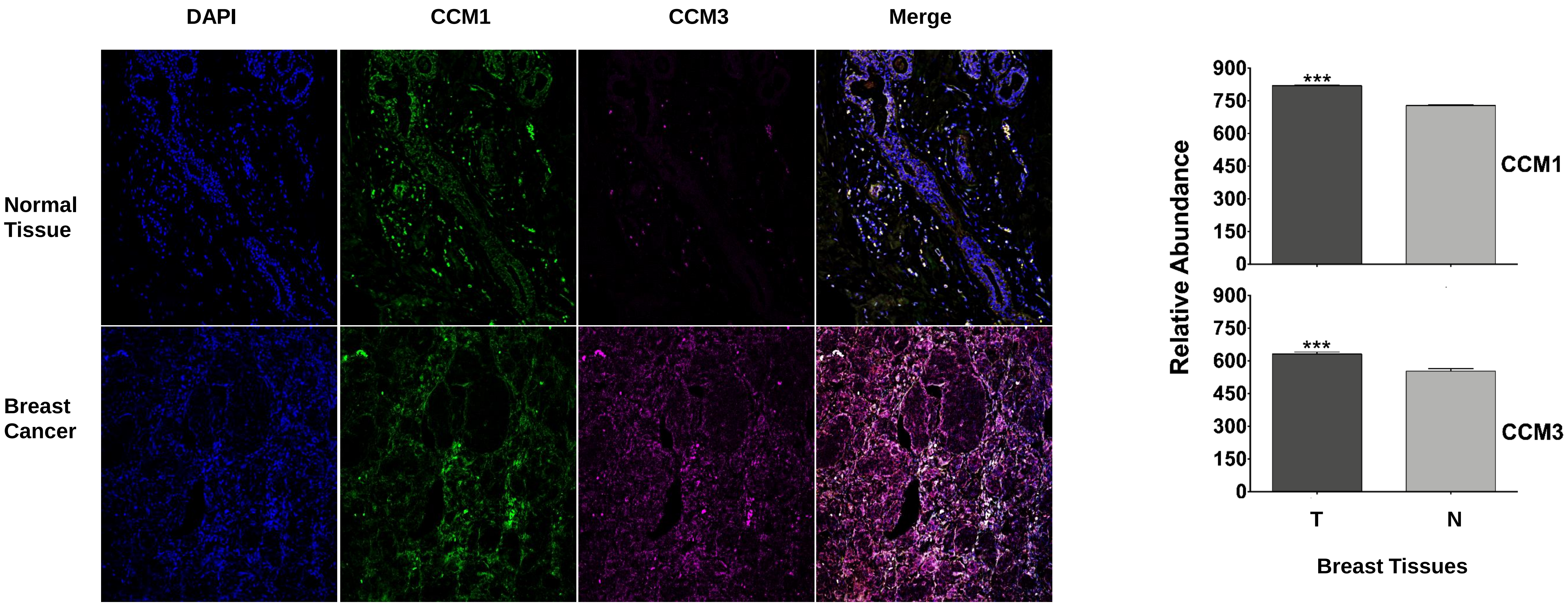
DAPI
CCM1
CCM3
Merge
Normal Tissue
Breast Cancer
Relative Abundance
900
750
600
450
300
150
0
***
CCM1
CCM3
T
N
Breast Tissues

S1C

Normal Breast Tissue

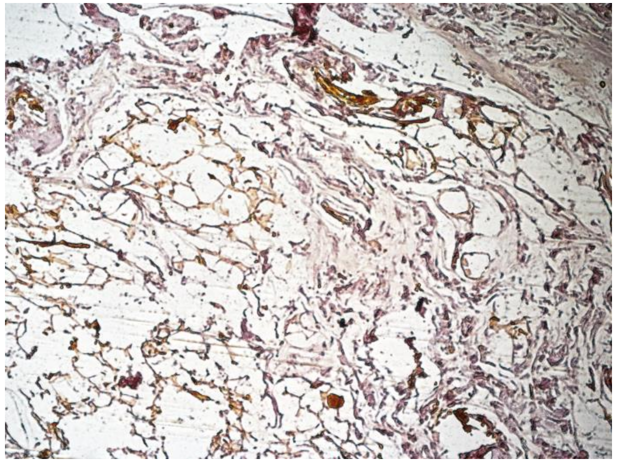

Breast Carcinoma

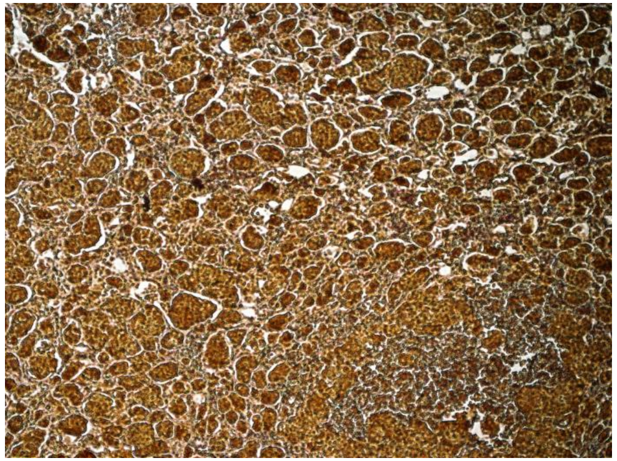

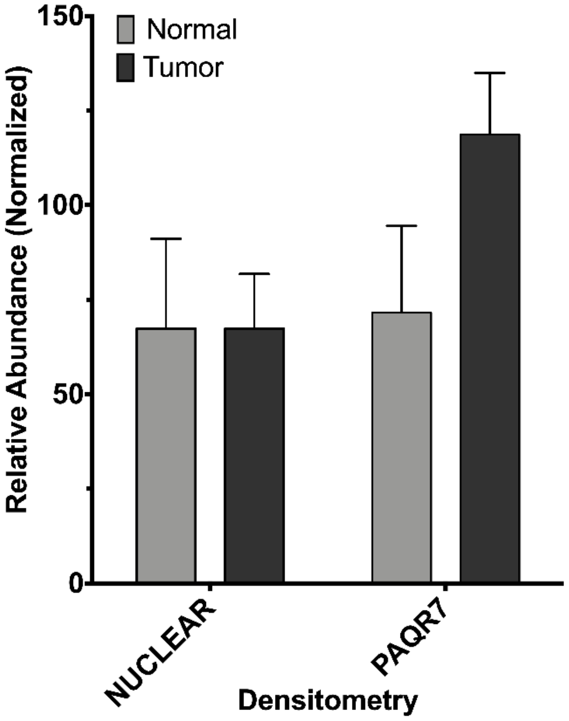

Breast Tissues

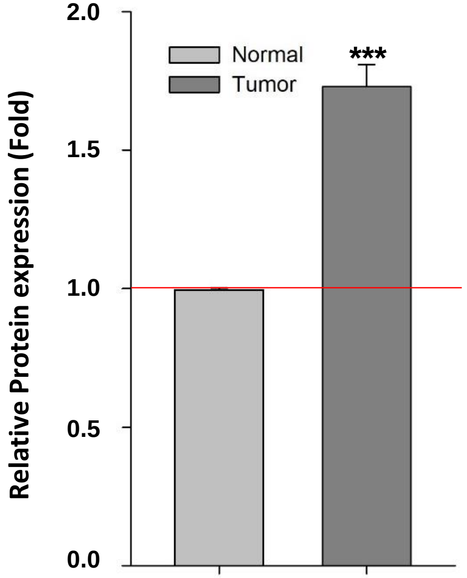

PAQR7 protein

Suppl. Fig. 2

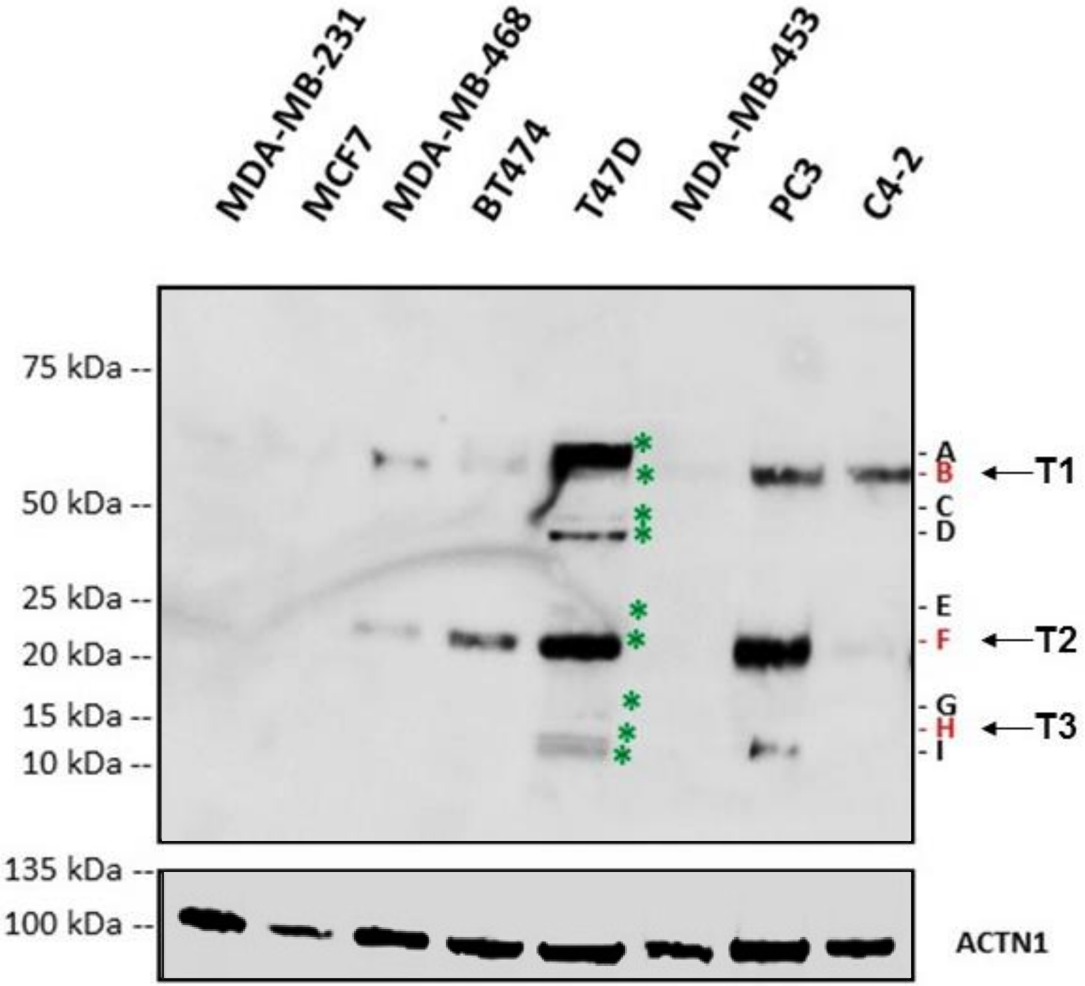

ER	–	†	–	†	†	–	†	†
PR	–	†	–	†	†	–	–	–
AR	†	†	†	†	†	†	–	†
GR	†	†	†	†	–	†	†	–
HER2	†	†	–	†	–	†	–	–

Suppl. Fig. 3A

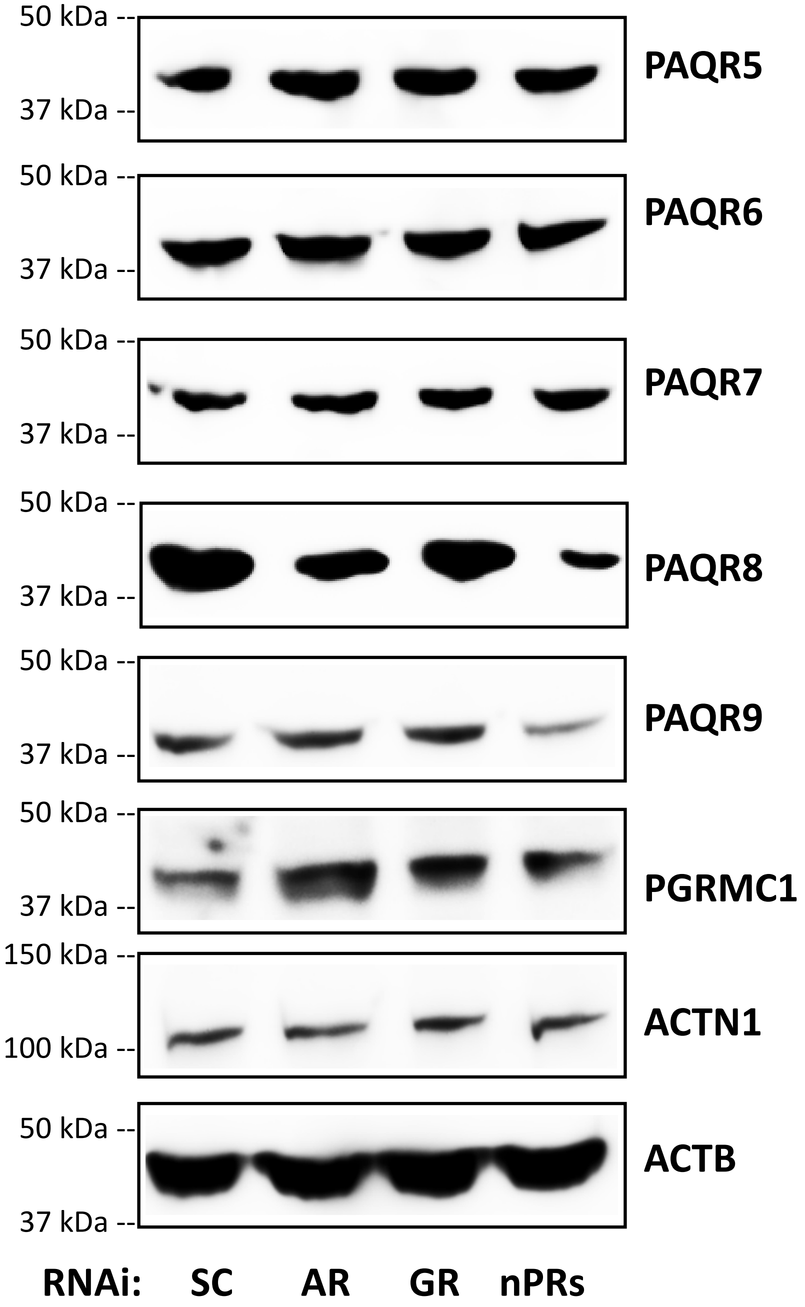

Suppl. Fig. 3B

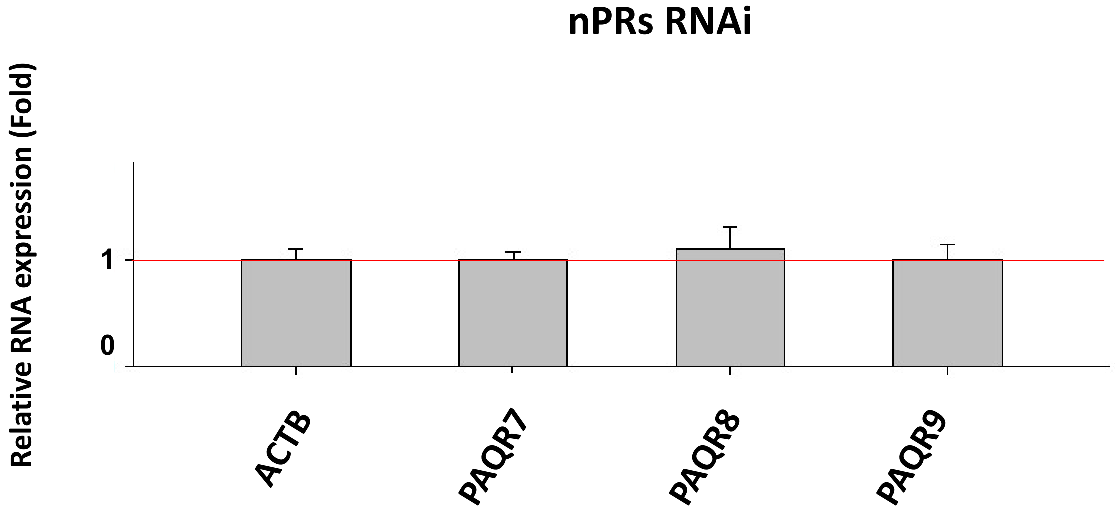

Suppl. Fig. 4A

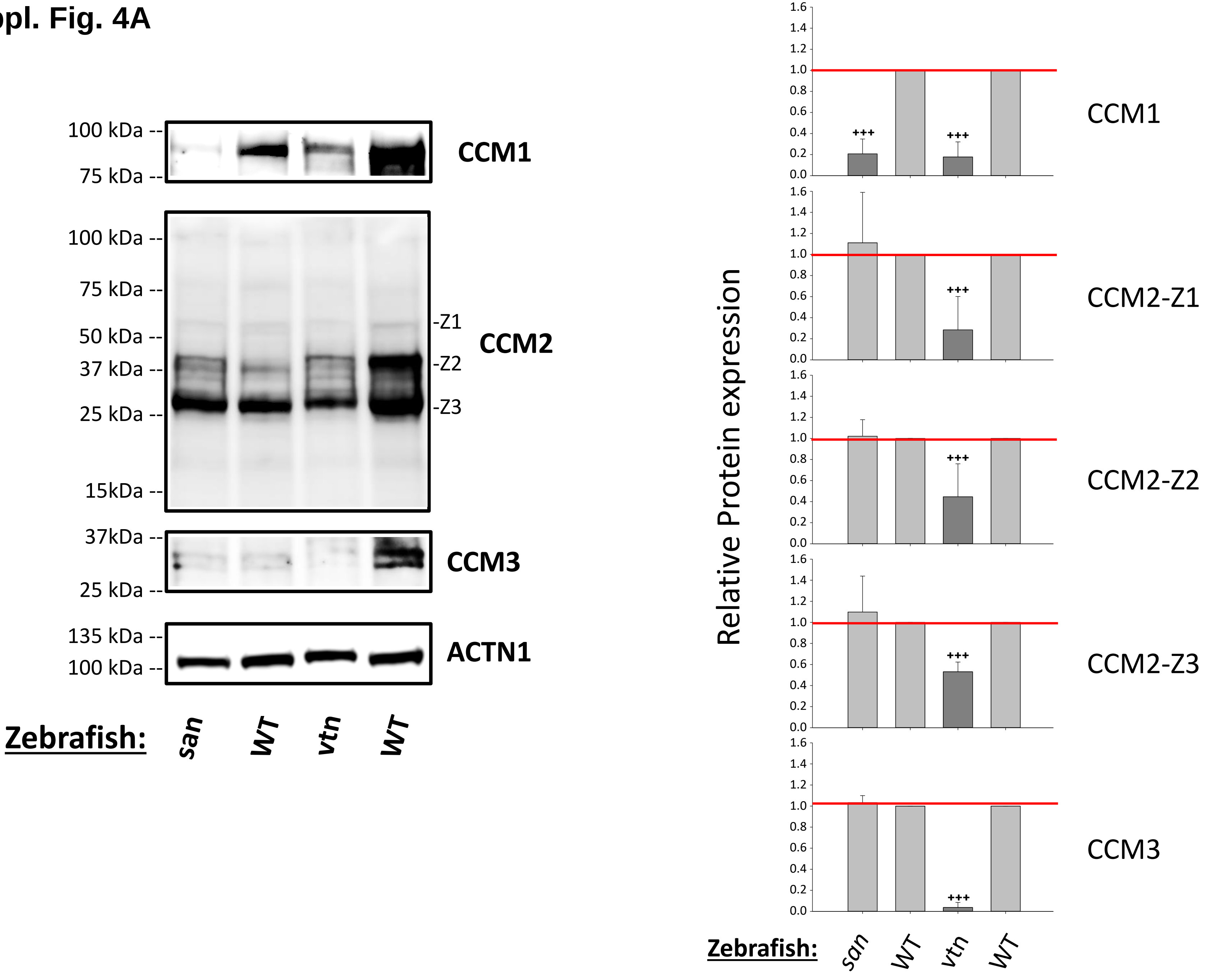
100 kDa
75 kDa
CCM1
100 kDa
75 kDa
50 kDa
37 kDa
25 kDa
15kDa
-Z1
-Z2
-Z3
CCM2
37kDa
25 kDa
CCM3
135 kDa
100 kDa
ACTN1
Zebrafish:
san
WT
vtn
WT
Relative Protein expression
+++
CCM1
CCM2-Z1
CCM2-Z2
CCM2-Z3
CCM3
0.0
0.2
0.4
0.6
0.8
1.0
1.2
1.4
1.6

Suppl. Fig. 4B

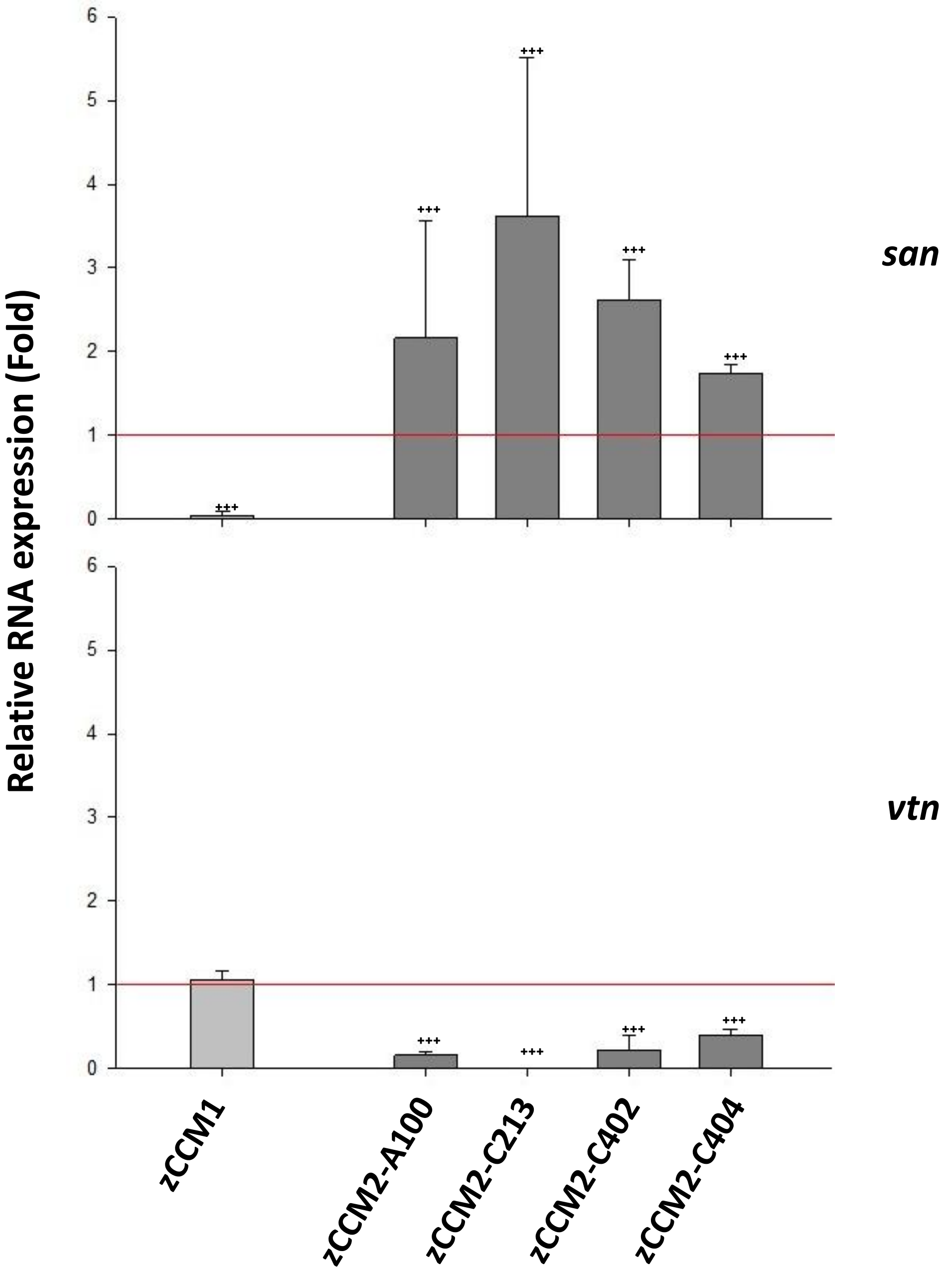

Relative RNA expression (Fold)
san
vtn
0
1
2
3
4
5
6
+++
zCCM1
zCCM2-A100
zCCM2-C213
zCCM2-C402
zCCM2-C404

Suppl. Fig 5

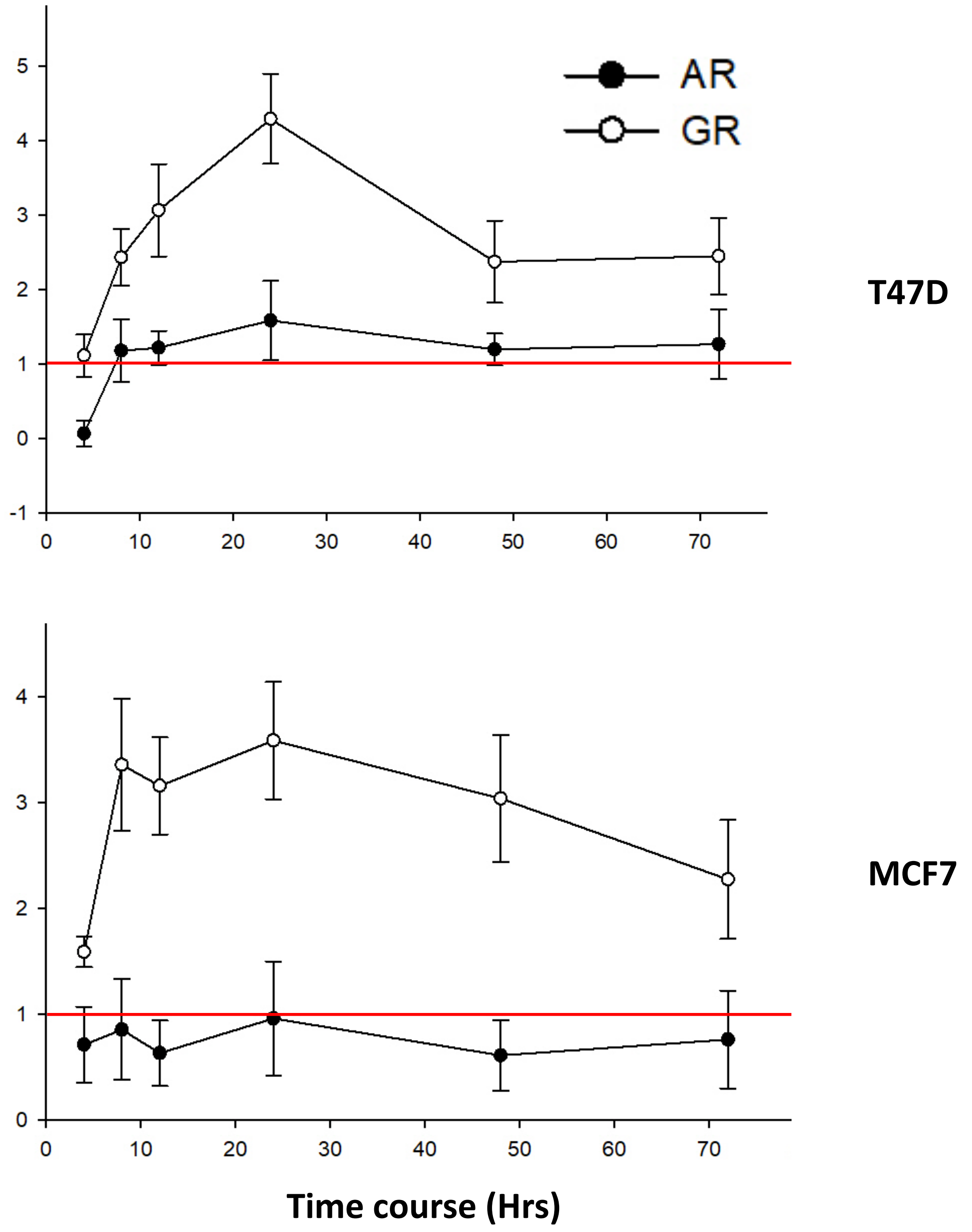

SUPPL. FIG. 6A-1

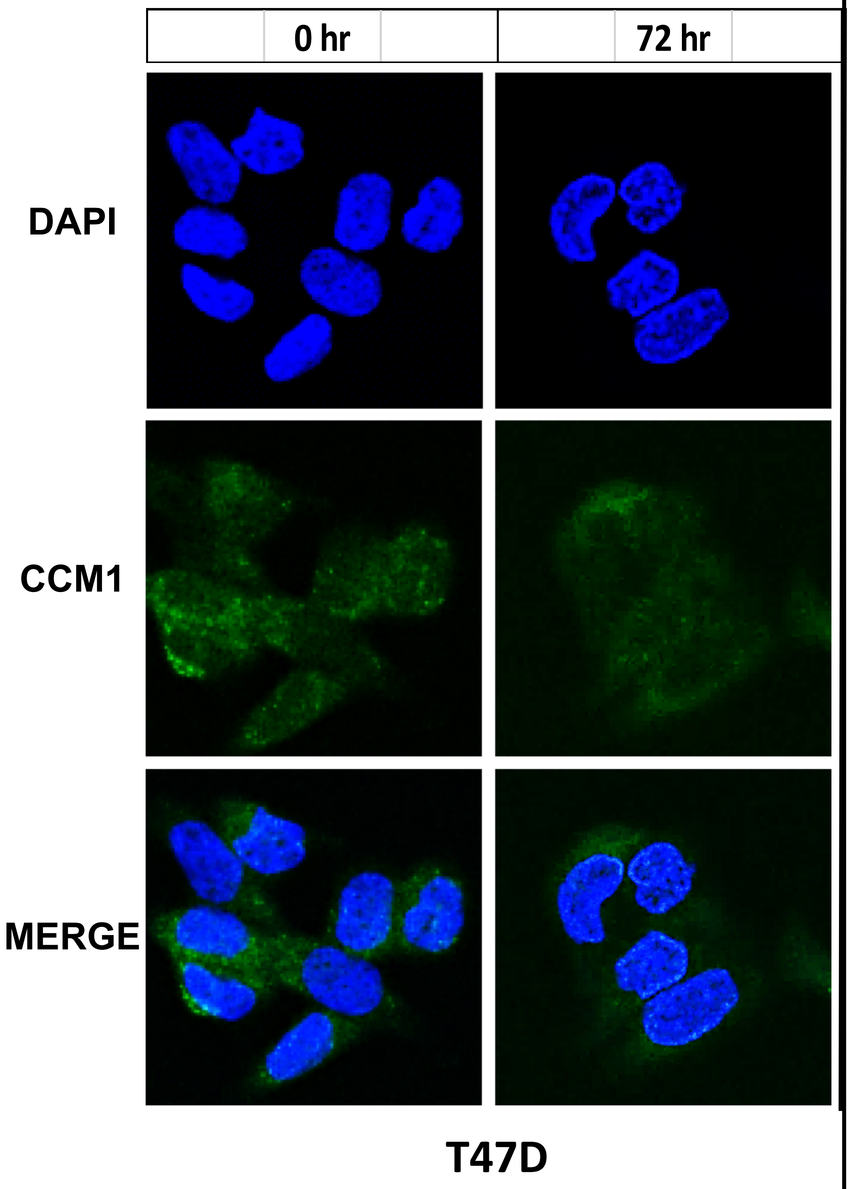

SUPPL. FIG. 6A-2

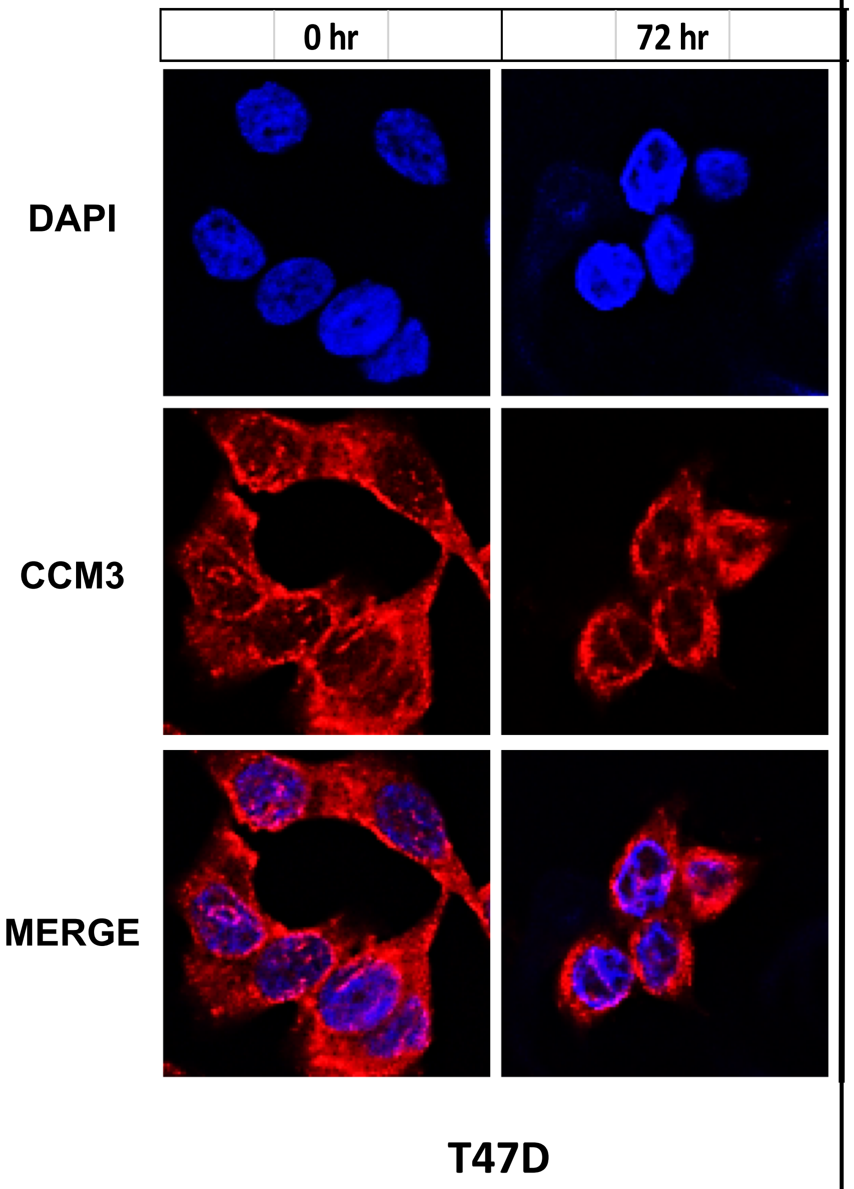

SUPPL. FIG. 6A-3

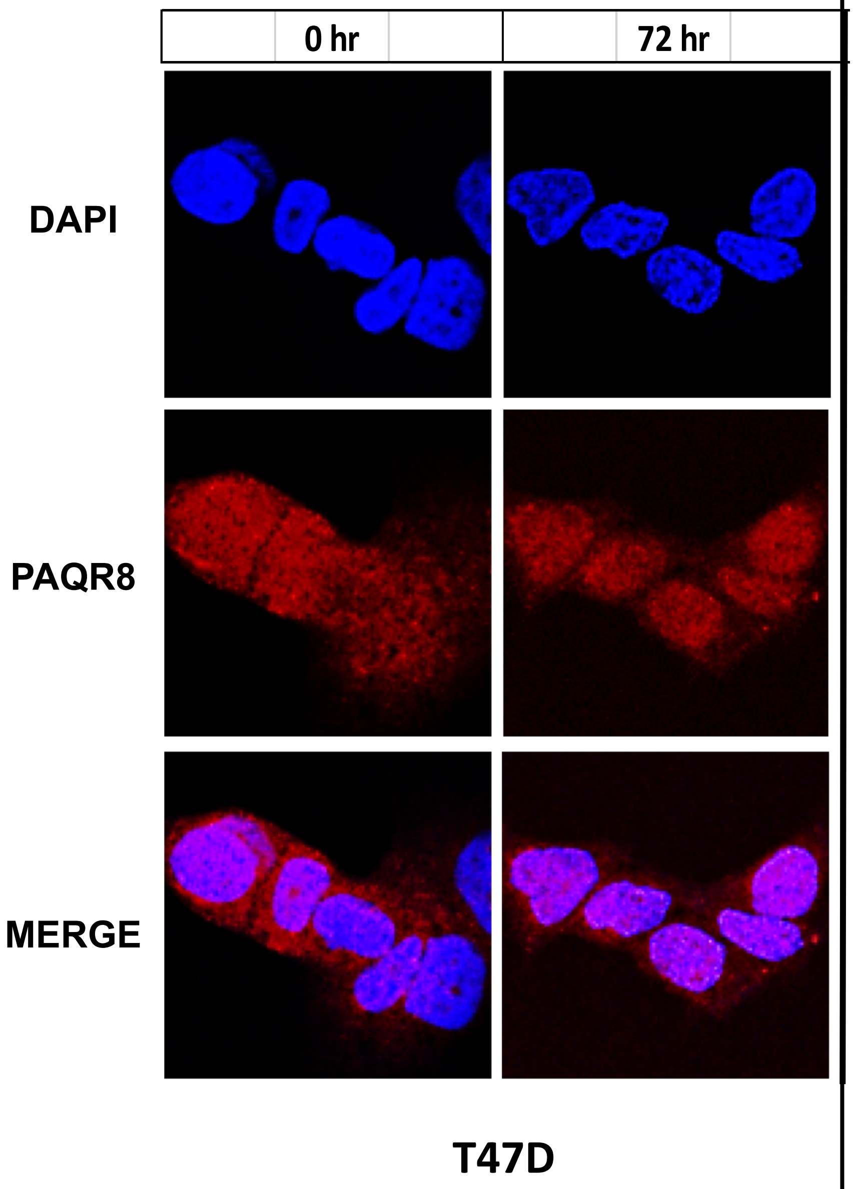

SUPPL. FIG. 6B-1

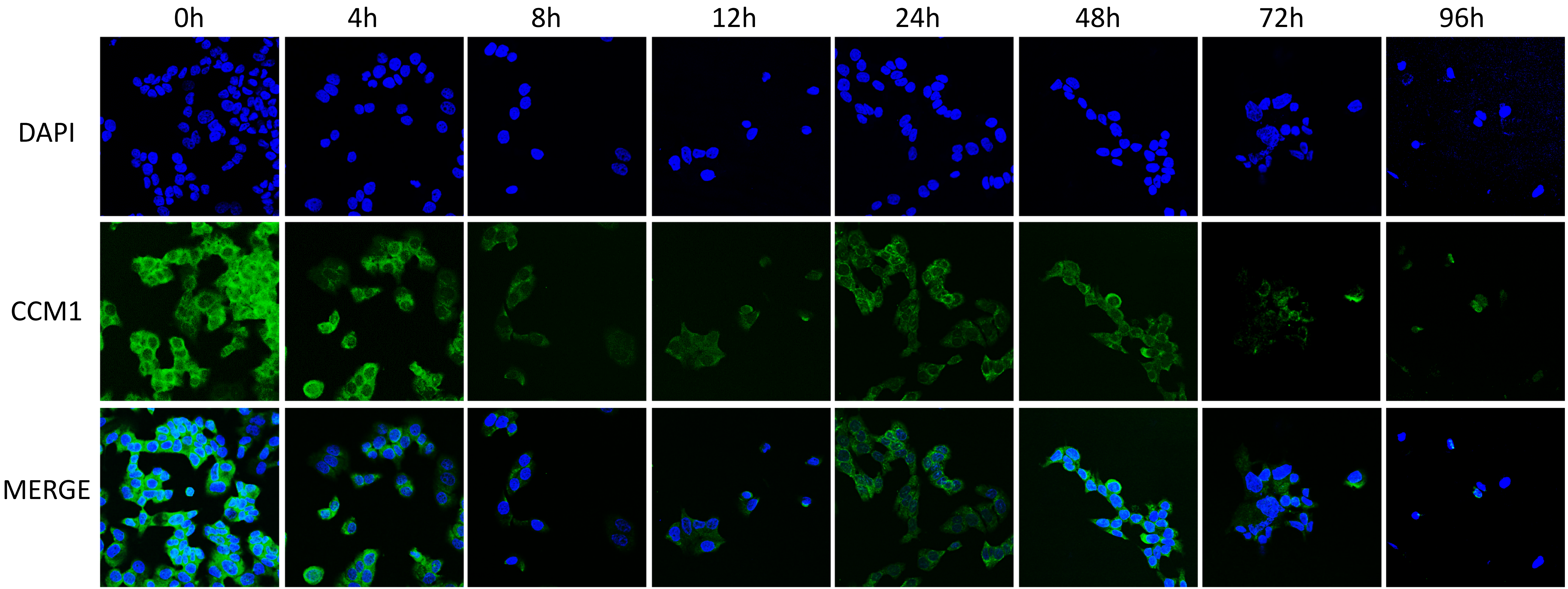

SUPPL. FIG. 6B-2

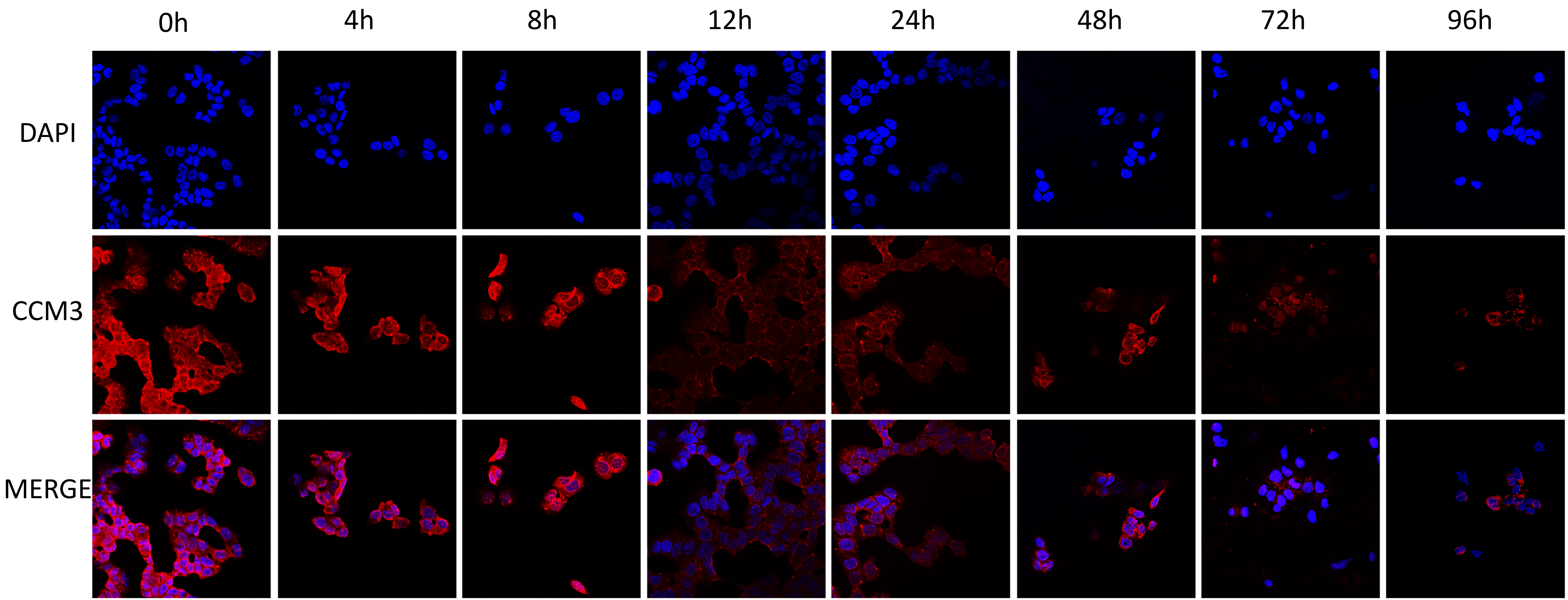

SUPPL. FIG. 6B-3

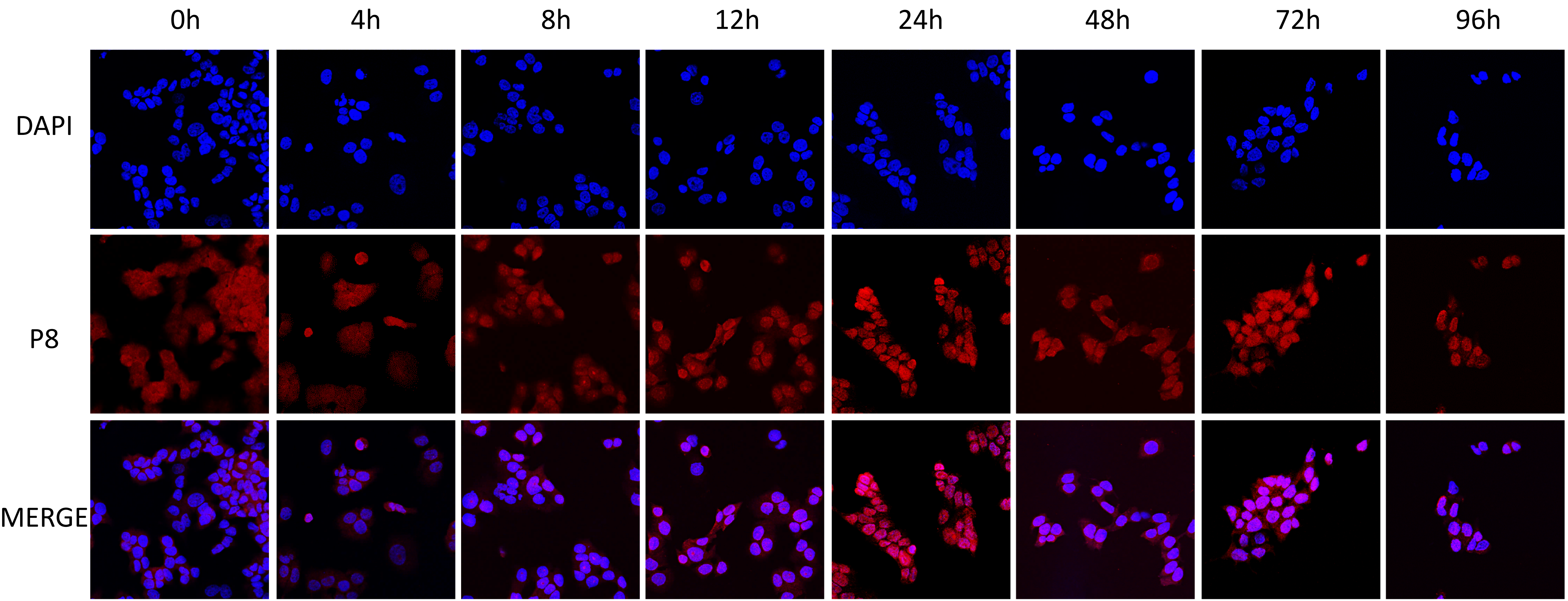

Suppl. Fig. 7

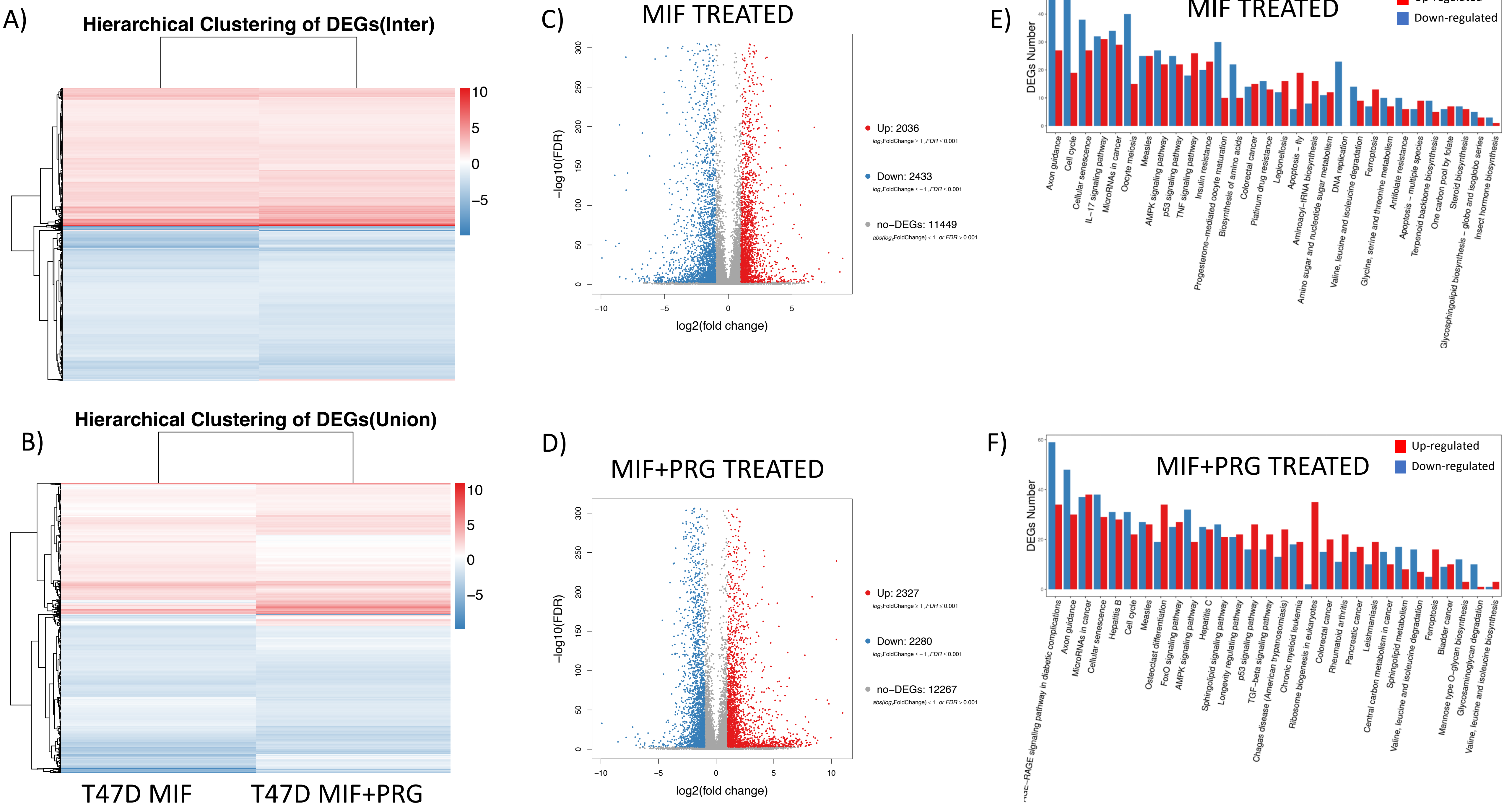
A)
Hierarchical Clustering of DEGs(Inter)
10
5
0
−5
B)
Hierarchical Clustering of DEGs(Union)
10
5
0
−5
T47D MIF
T47D MIF+PRG
C)
MIF TREATED
−log10(FDR)
log2(fold change)
Up: 2036
Down: 2433
no−DEGs: 11449
D)
MIF+PRG TREATED
−log10(FDR)
log2(fold change)
Up: 2327
Down: 2280
no−DEGs: 12267
E)
MIF TREATED
Up-regulated
Down-regulated
DEGs Number
Axon guidance
Cell cycle
Cellular senescence
IL−17 signaling pathway
MicroRNAs in cancer
Oocyte meiosis
Measles
AMPK signaling pathway
p53 signaling pathway
TNF signaling pathway
Insulin resistance
Progesterone−mediated oocyte maturation
Biosynthesis of amino acids
Colorectal cancer
Platinum drug resistance
Legionellosis
Apoptosis − fly
Aminoacyl−tRNA biosynthesis
Amino sugar and nucleotide sugar metabolism
DNA replication
Valine, leucine and isoleucine degradation
Ferroptosis
Glycine, serine and threonine metabolism
Antifolate resistance
Apoptosis − multiple species
Terpenoid backbone biosynthesis
One carbon pool by folate
Steroid biosynthesis
Glycosphingolipid biosynthesis − globo and isoglobo series
Insect hormone biosynthesis
F)
MIF+PRG TREATED
Up-regulated
Down-regulated
DEGs Number
Axon guidance
MicroRNAs in cancer
Cellular senescence
Hepatitis B
Cell cycle
Measles
Osteoclast differentiation
FoxO signaling pathway
AMPK signaling pathway
Hepatitis C
Sphingolipid signaling pathway
Longevity regulating pathway
p53 signaling pathway
TGF−beta signaling pathway
Chagas disease (American trypanosomiasis)
Chronic myeloid leukemia
Ribosome biogenesis in eukaryotes
Colorectal cancer
Rheumatoid arthritis
Pancreatic cancer
Leishmaniasis
Central carbon metabolism in cancer
Sphingolipid metabolism
Valine, leucine and isoleucine degradation
Ferroptosis
Bladder cancer
Mannose type O−glycan biosynthesis
Glycosaminoglycan degradation
Valine, leucine and isoleucine biosynthesis

Suppl. Fig. 8

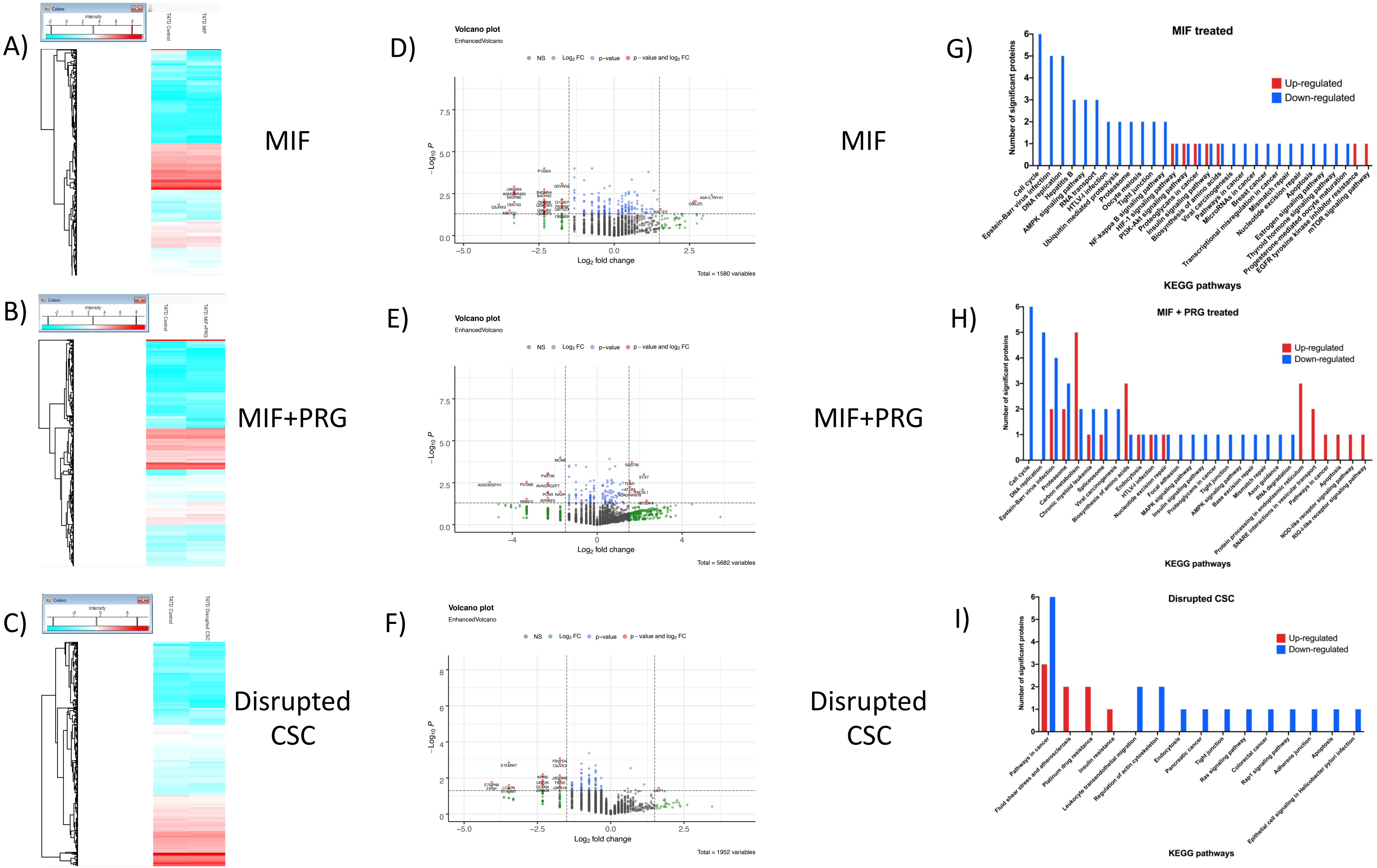
A)
B)
C)
D)
E)
F)
G)
H)
I)
MIF
MIF+PRG
Disrupted CSC
Volcano plot
EnhancedVolcano
NS
Log2 FC
p-value
p - value and log2 FC
Log2 fold change
Total = 1580 variables
Total = 5682 variables
Total = 1952 variables
MIF treated
MIF + PRG treated
Disrupted CSC
Up-regulated
Down-regulated
Number of significant proteins
KEGG pathways

Suppl. Fig. 9A

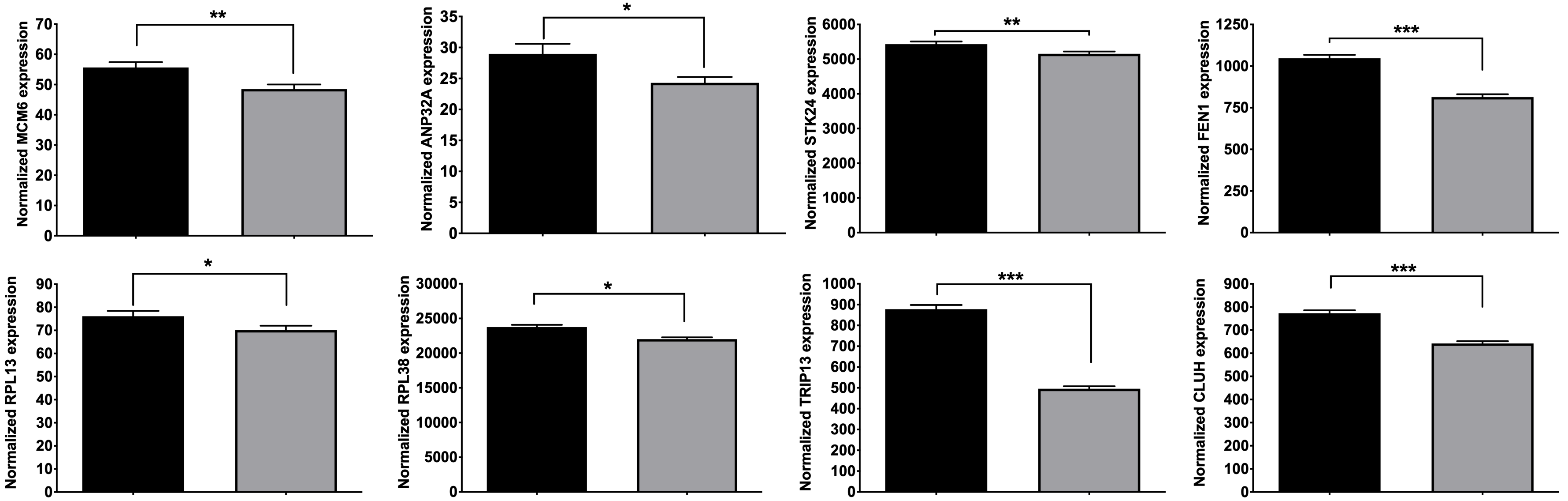

Low in nPR(+)

Suppl. Fig. 9B

High in nPR(+)

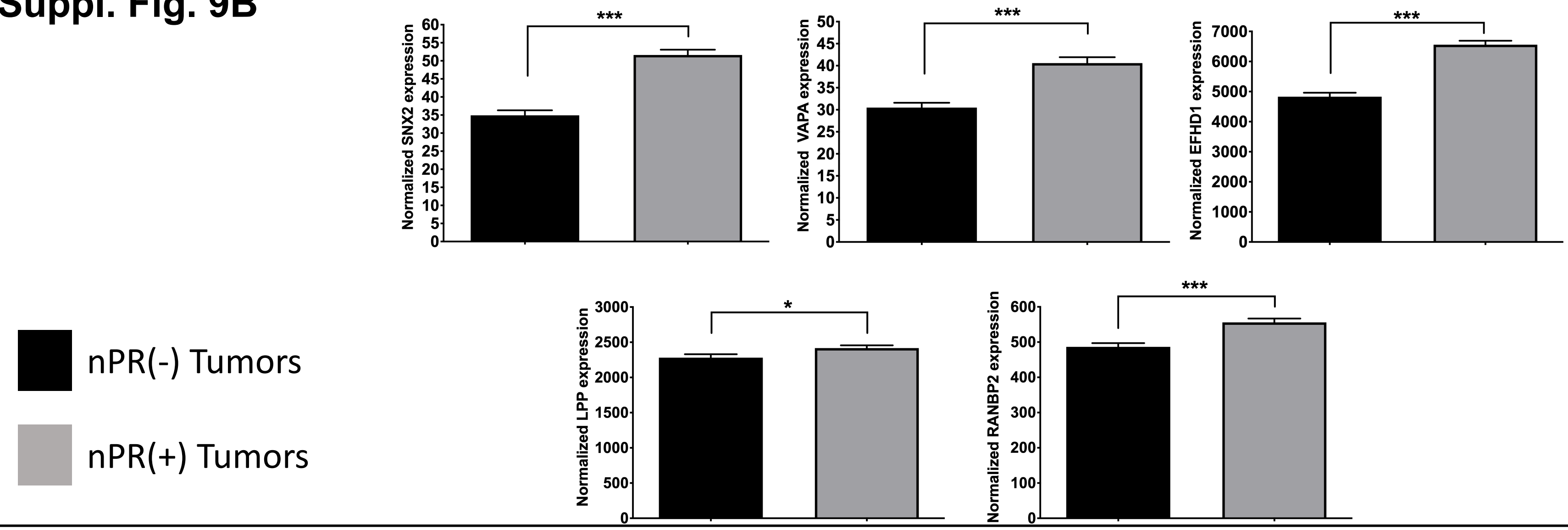

Suppl. Fig. 10A

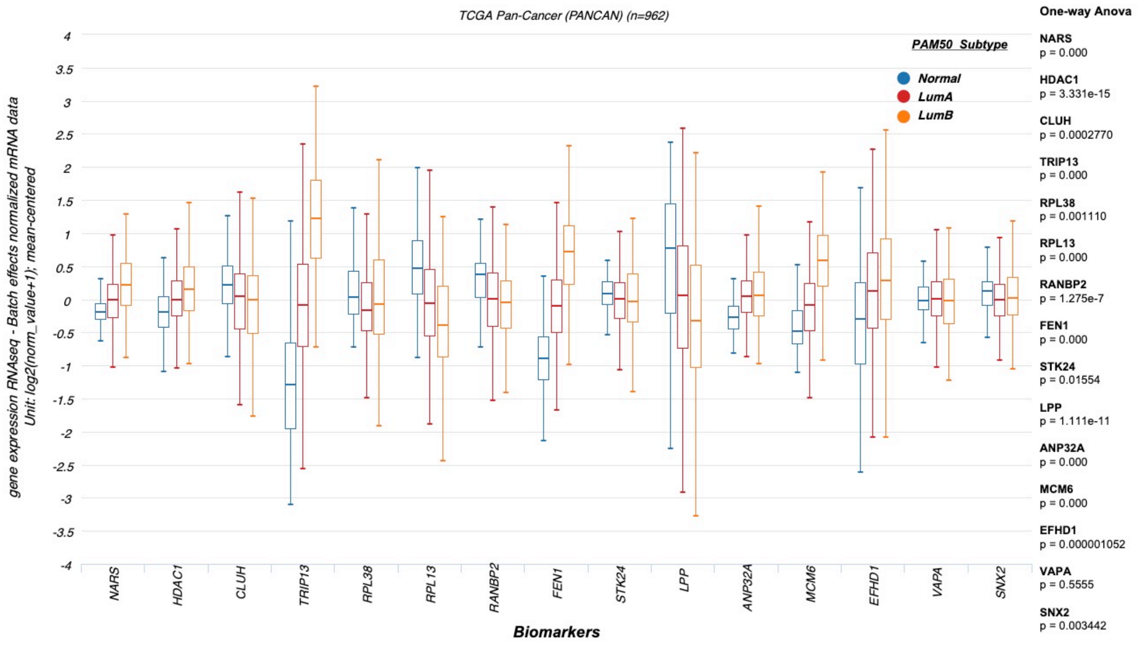

Suppl. Fig. 10B

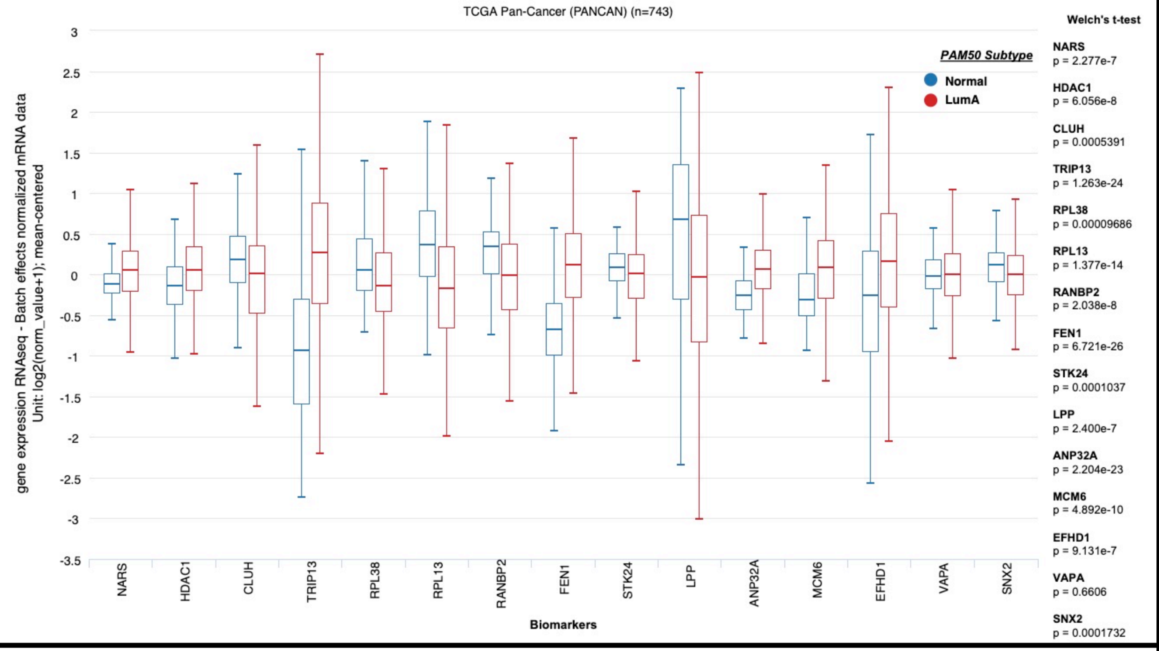

Suppl. Fig. 10C

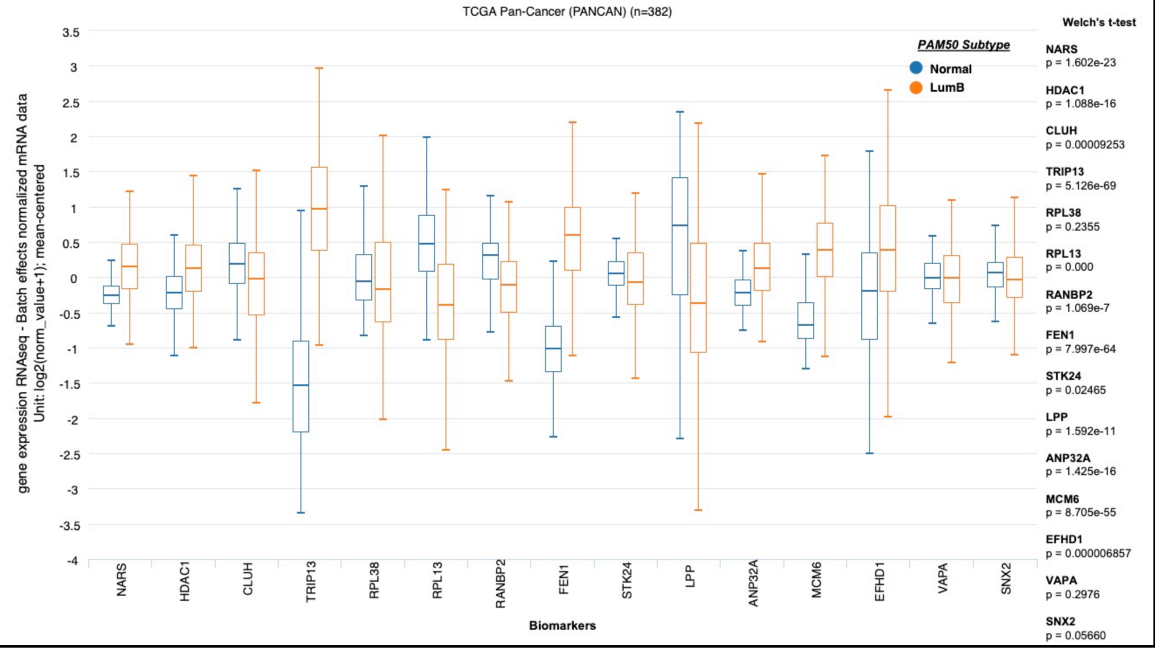

Suppl. Fig. 10D

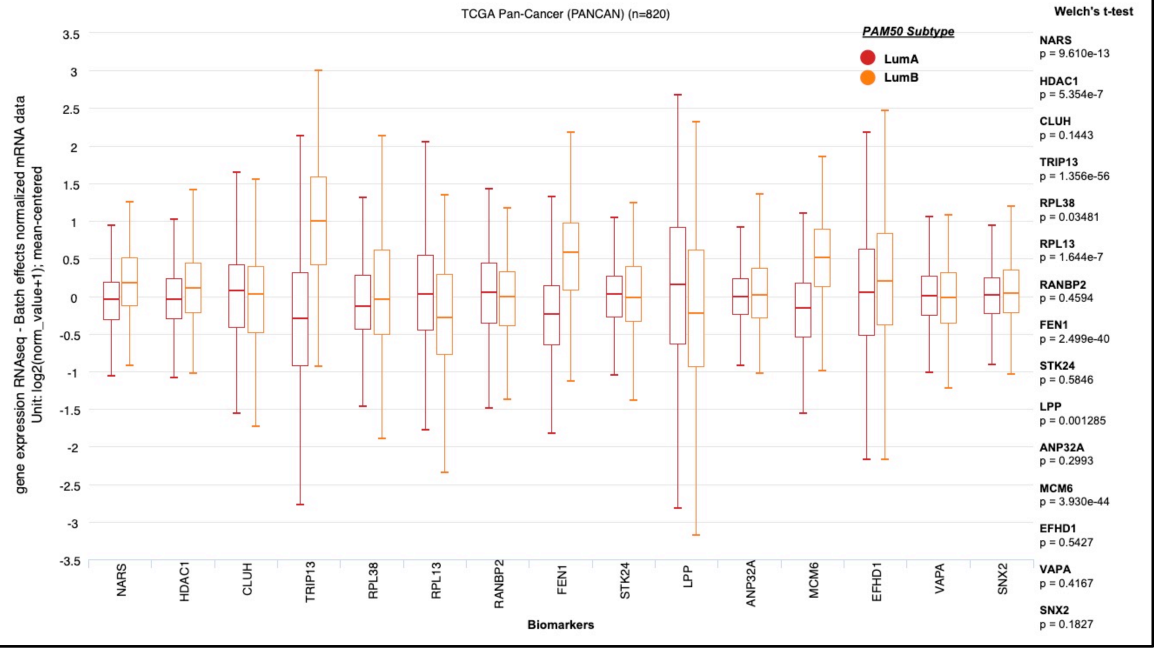

Suppl. Fig. 11

Filter: Luminal-A: 1809/631 patients(depending on probe)

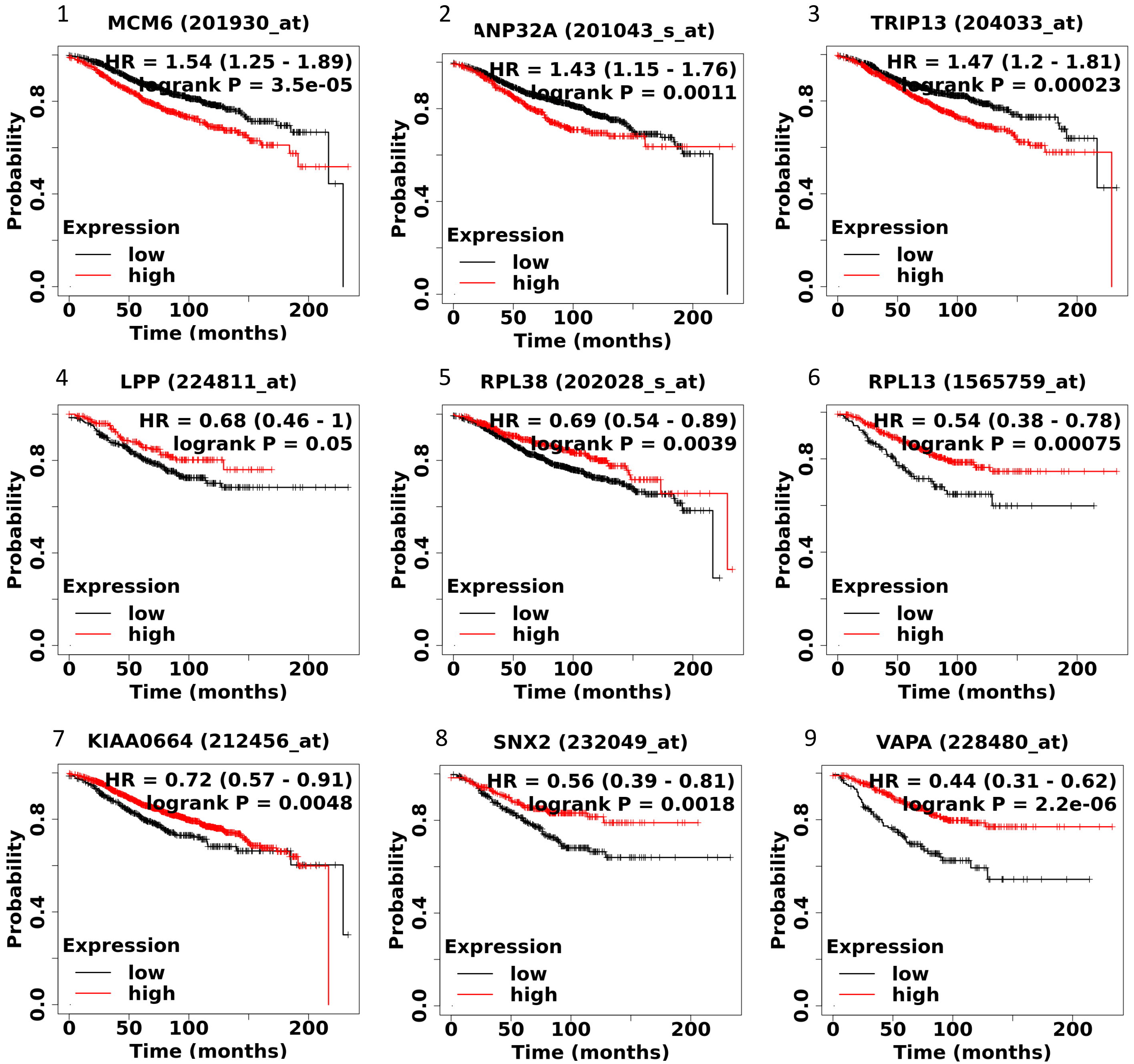

Suppl. Fig. 12

Filter: Normal: 119/35 patients (depending on probe)

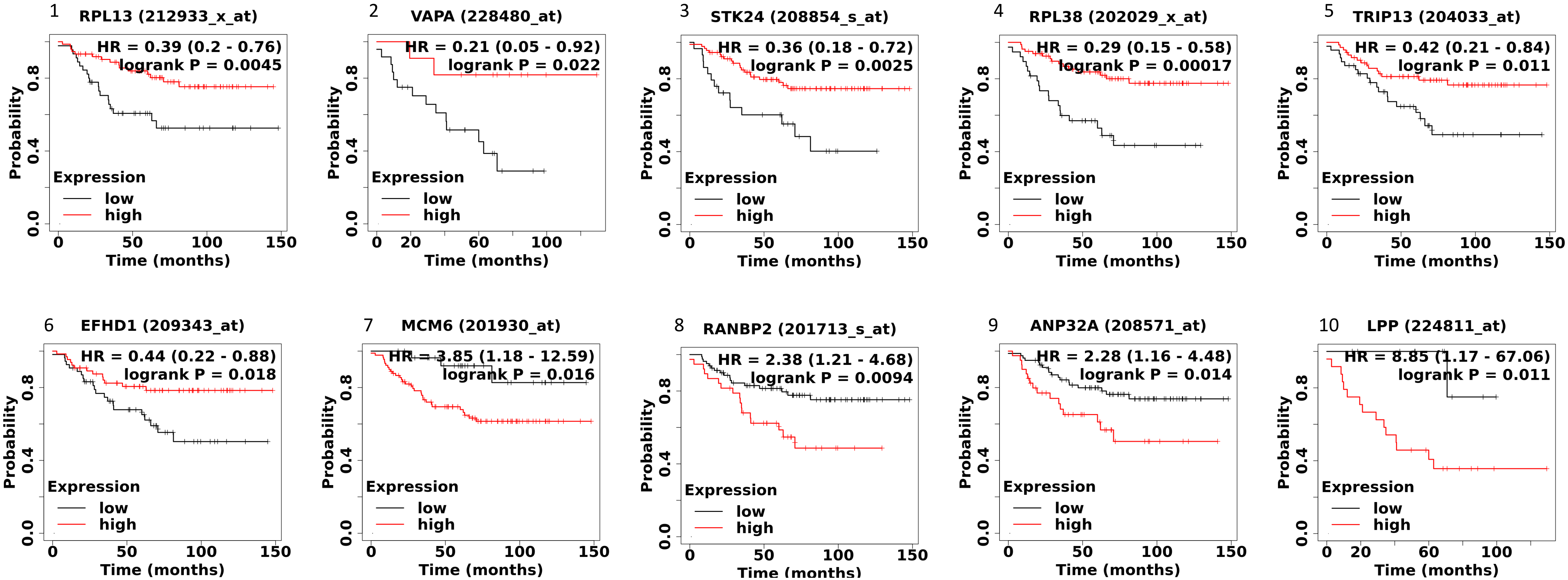

3A-1

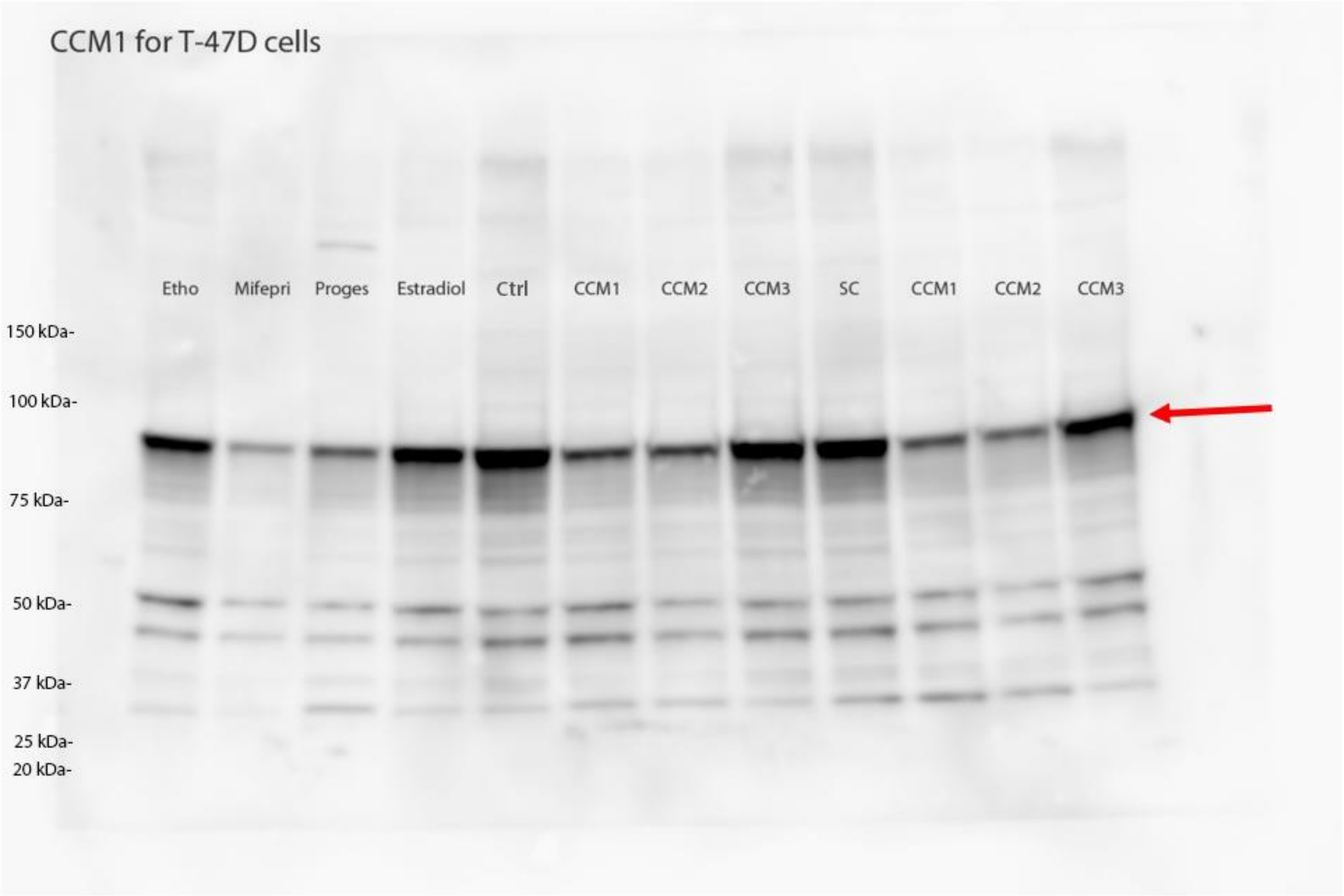
CCM1 for T-47D cells
Etho
Mifepri
Proges
Estradiol
Ctrl
CCM1
CCM2
CCM3
SC
CCM1
CCM2
CCM3
150 kDa-
100 kDa-
75 kDa-
50 kDa-
37 kDa-
25 kDa-
20 kDa-

3A-2

Wet transfer

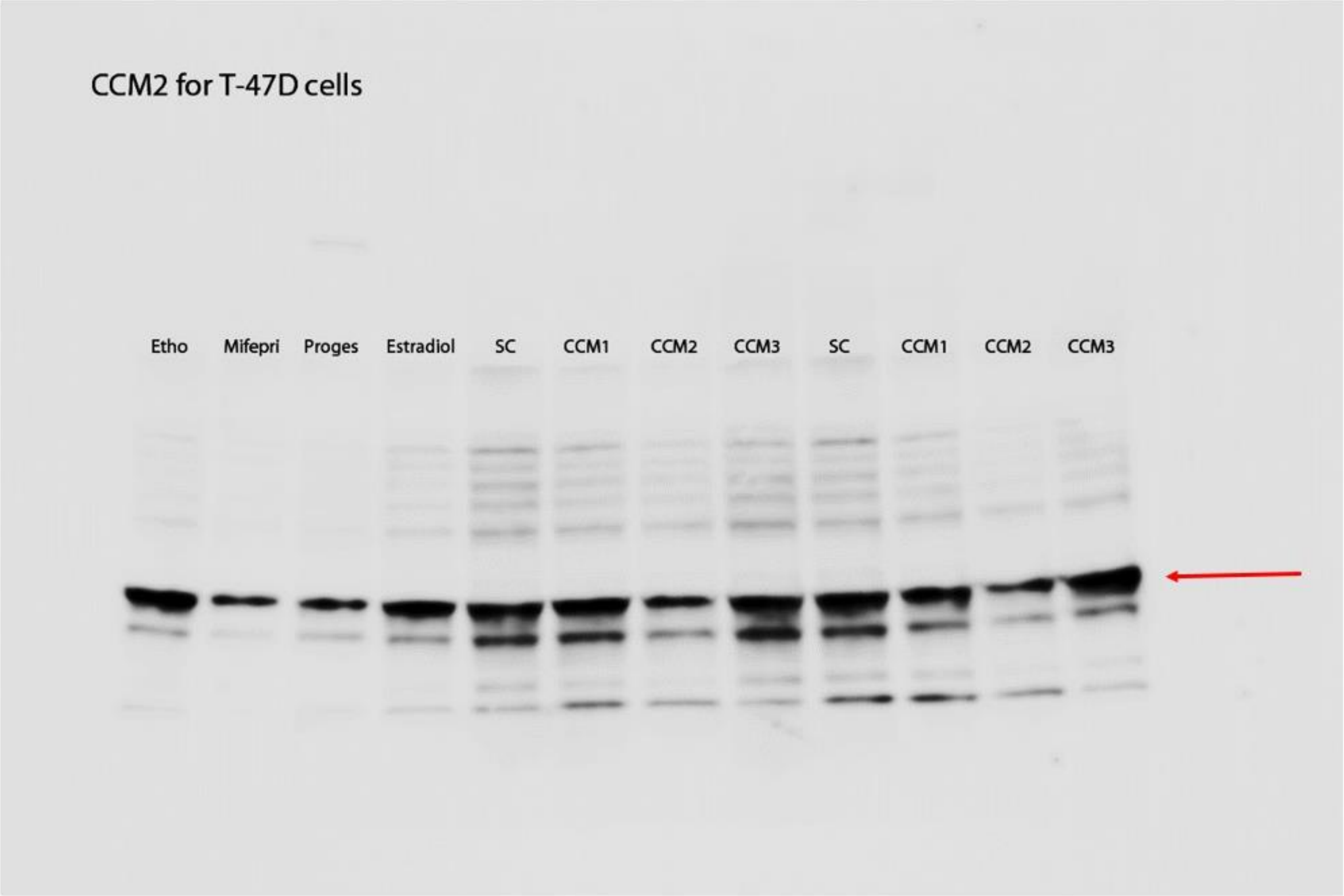

3A-3

Wet transfer

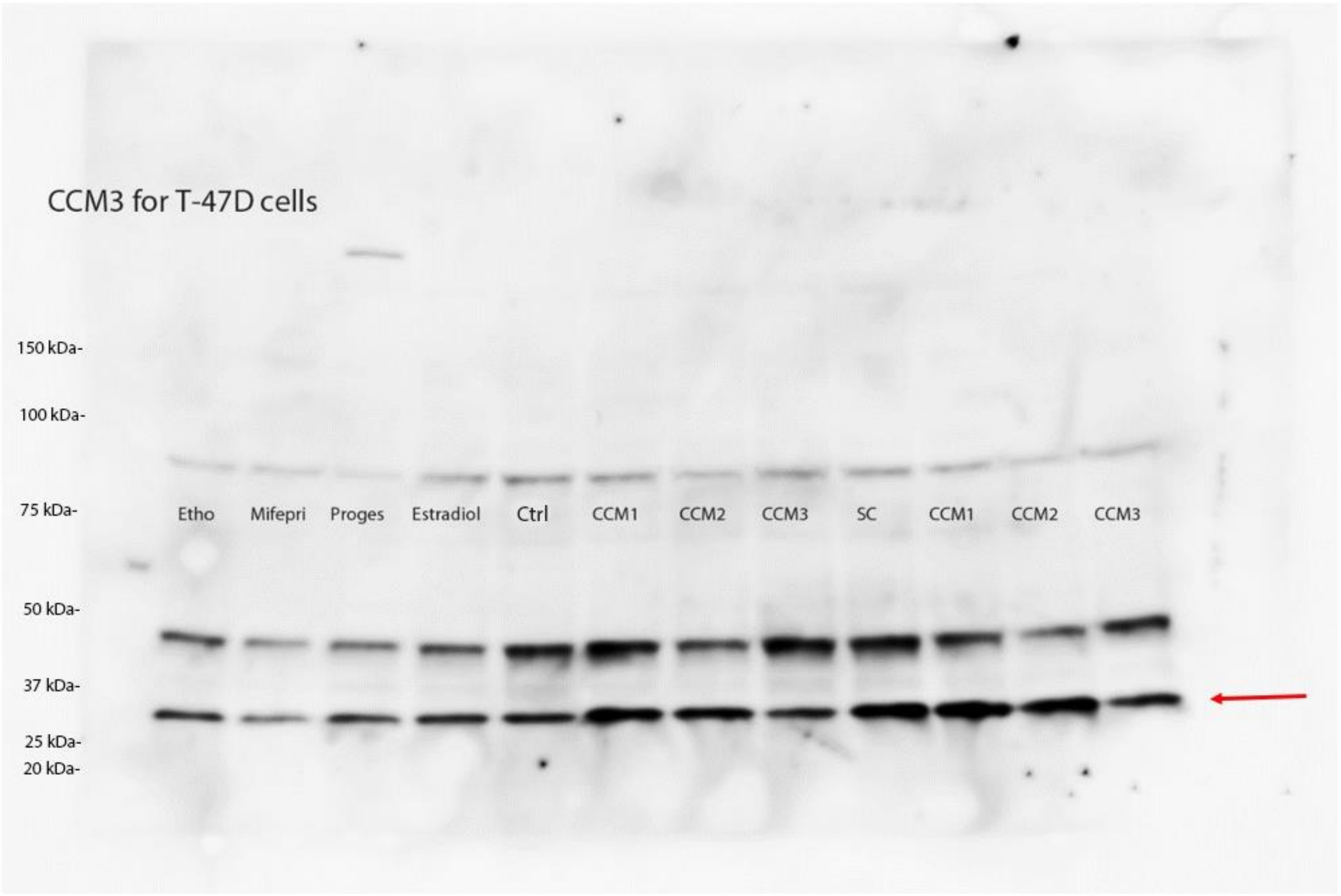

3A-4

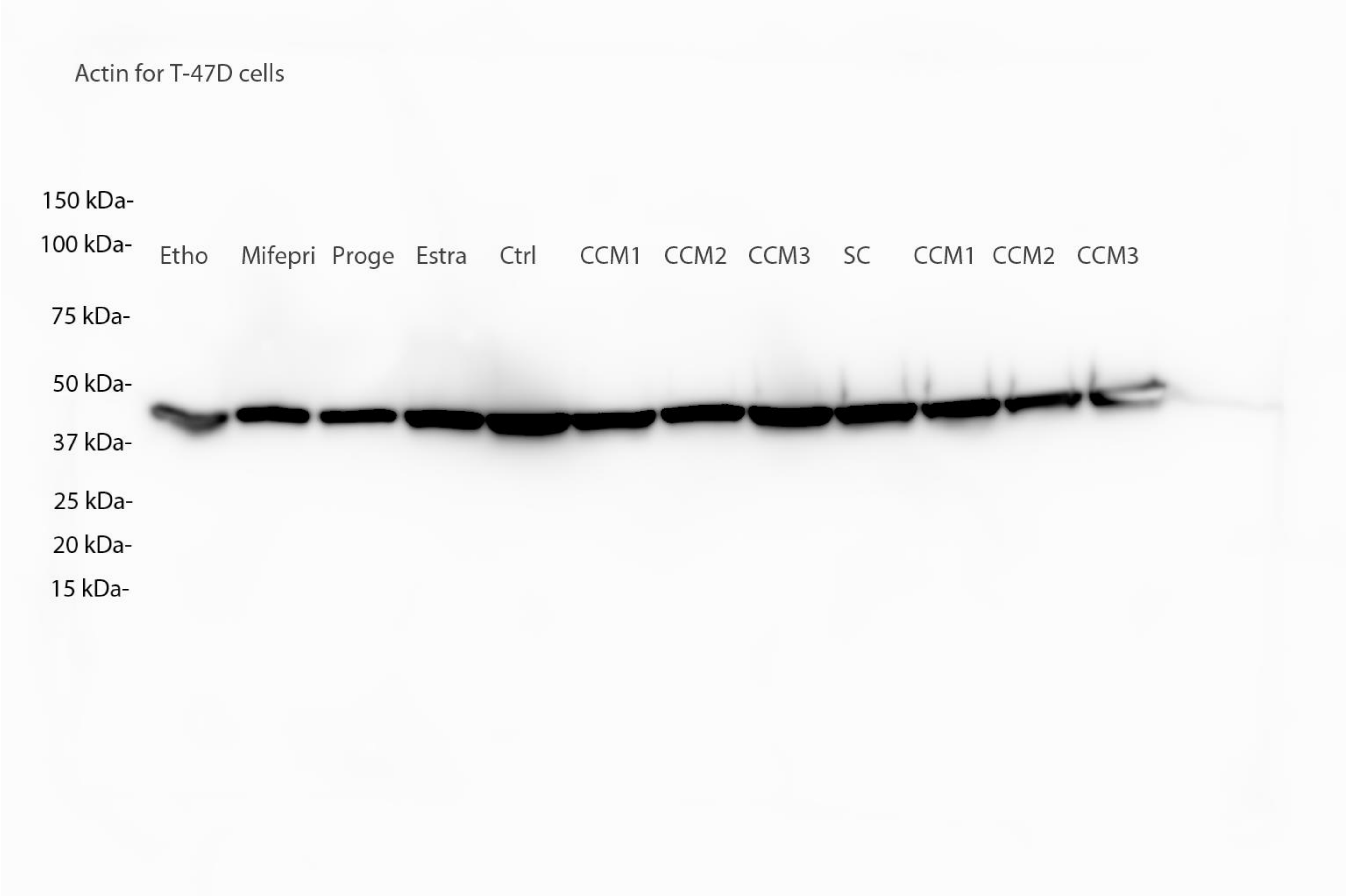
Actin for T-47D cells
150 kDa-
100 kDa-
75 kDa-
50 kDa-
37 kDa-
25 kDa-
20 kDa-
15 kDa-
Etho
Mifepri
Proge
Estra
Ctrl
CCM1
CCM2
CCM3
SC
CCM1
CCM2
CCM3

3B-1

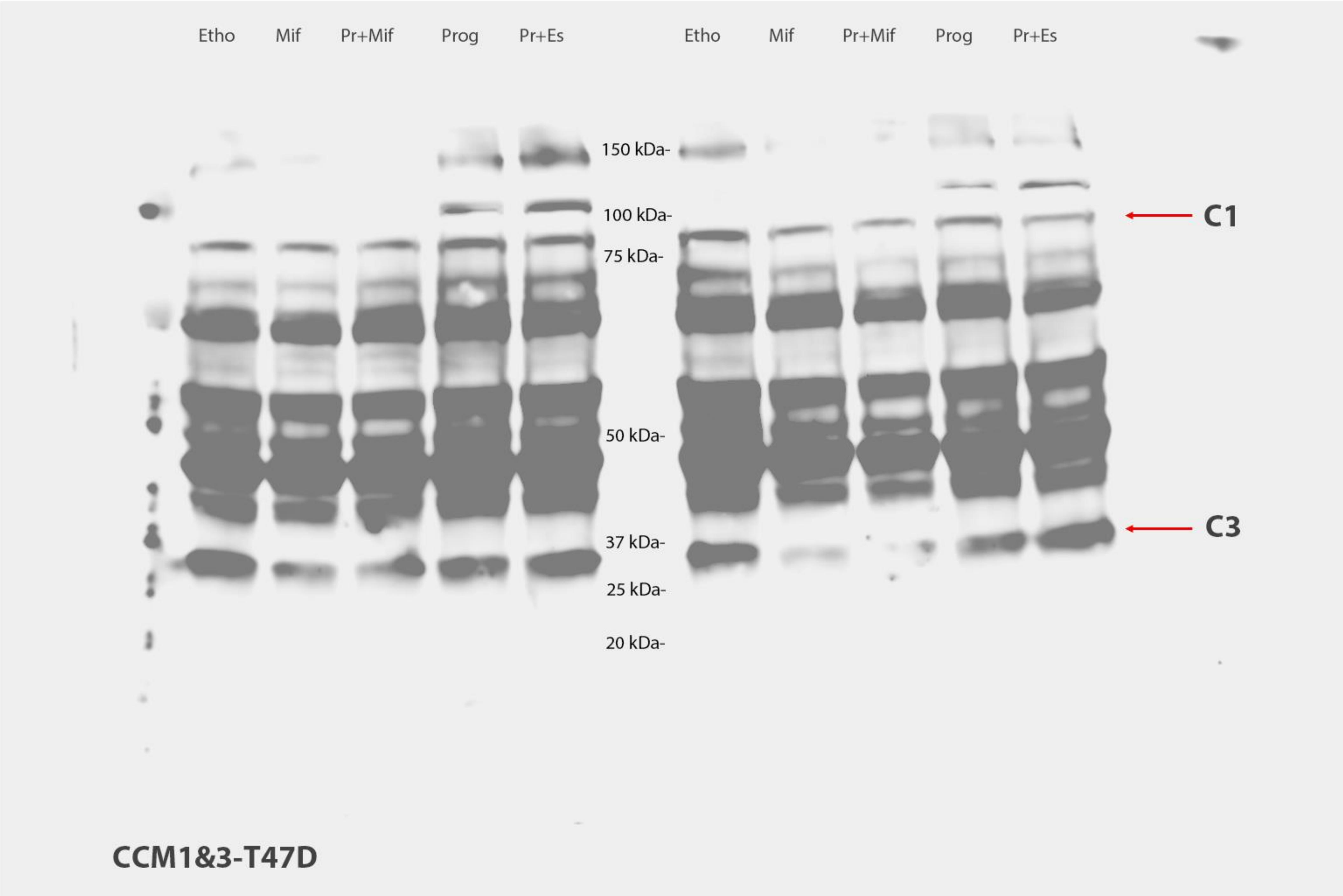
Etho
Mif
Pr+Mif
Prog
Pr+Es
Etho
Mif
Pr+Mif
Prog
Pr+Es
150 kDa-
100 kDa-
75 kDa-
50 kDa-
37 kDa-
25 kDa-
20 kDa-
C1
C3
CCM1&3-T47D

3B-2

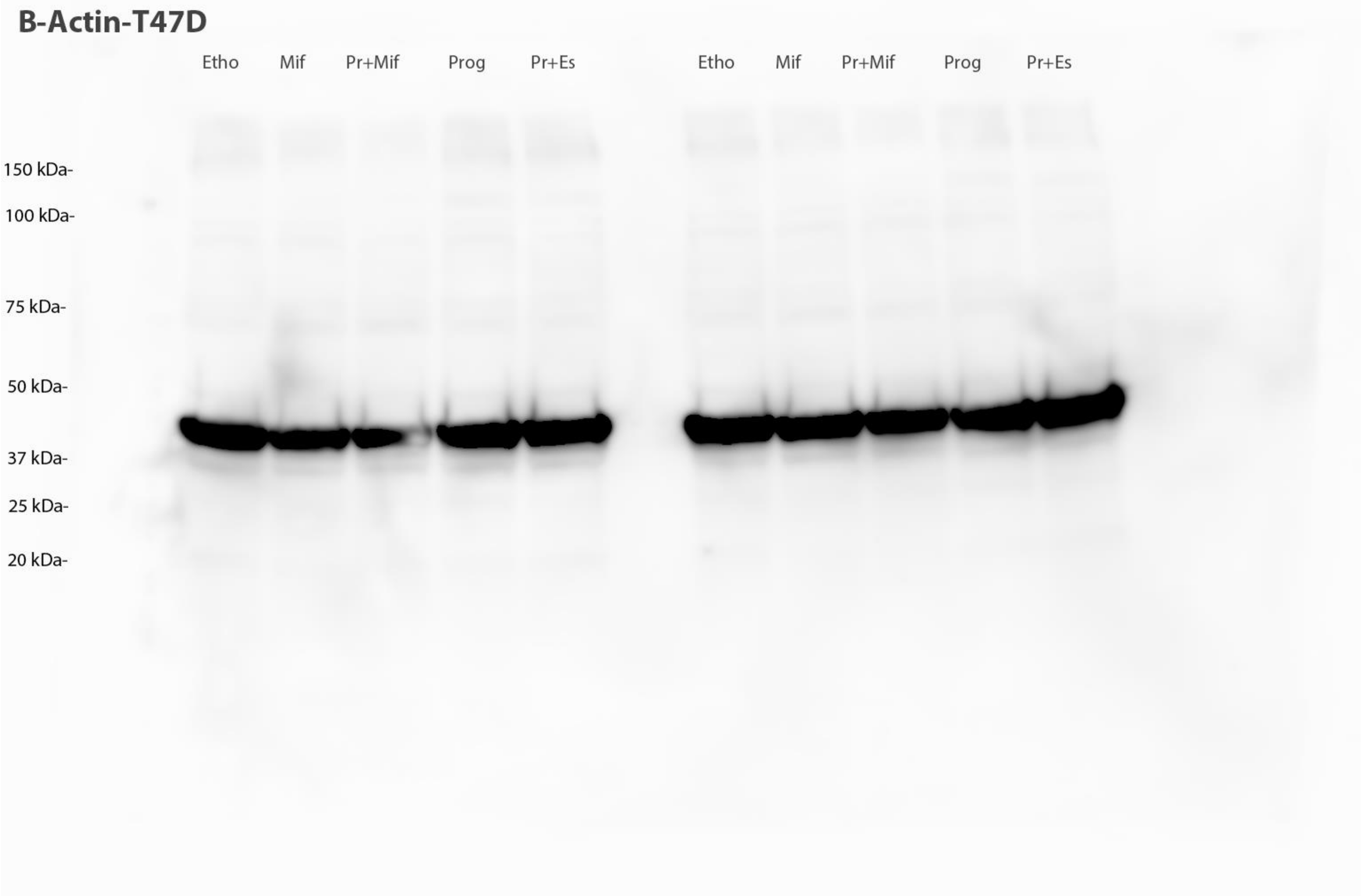
B-Actin-T47D
Etho
Mif
Pr+Mif
Prog
Pr+Es
Etho
Mif
Pr+Mif
Prog
Pr+Es
150 kDa-
100 kDa-
75 kDa-
50 kDa-
37 kDa-
25 kDa-
20 kDa-

3D11-1

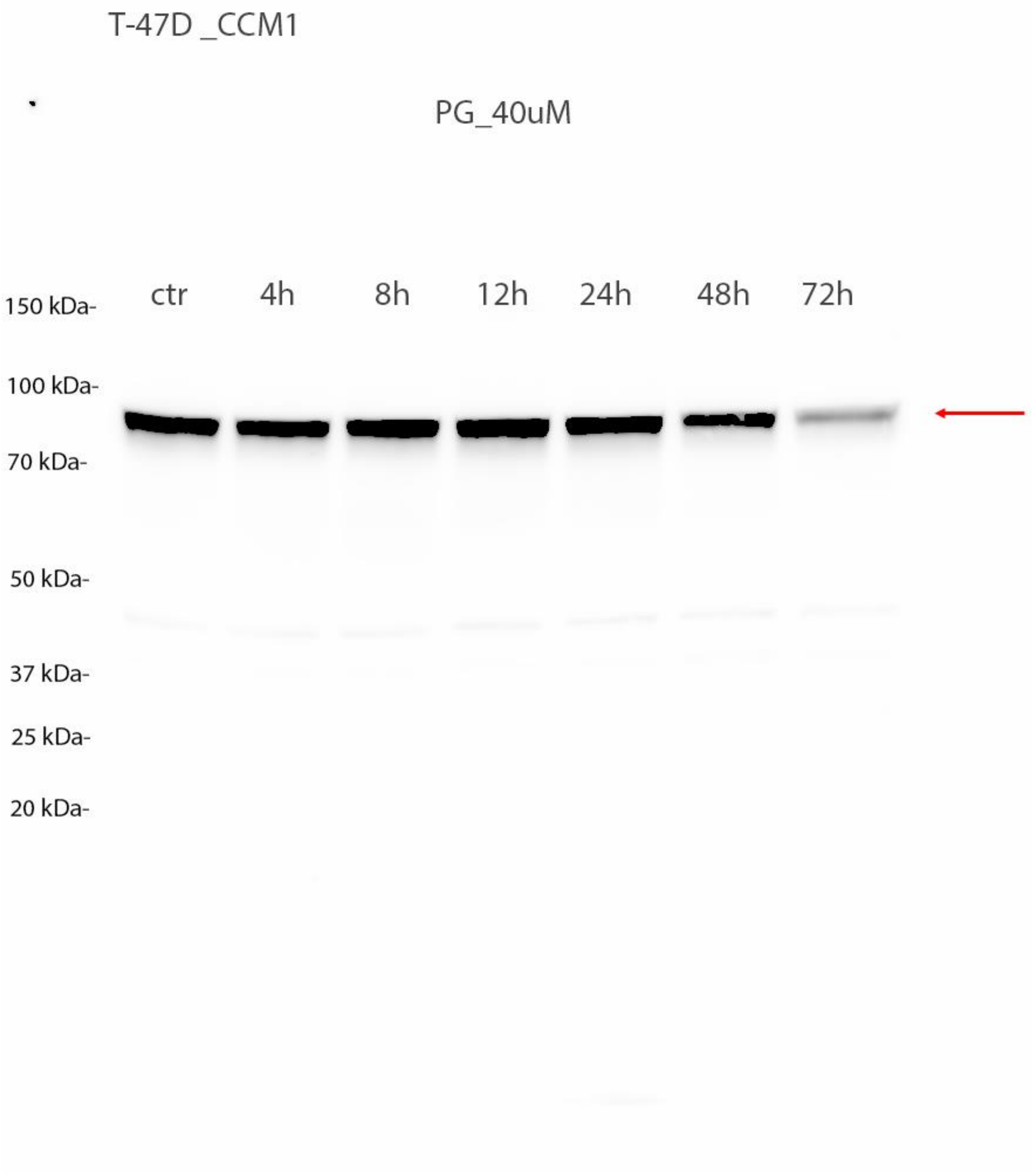
T-47D _CCM1
PG_40uM
150 kDa-
100 kDa-
70 kDa-
50 kDa-
37 kDa-
25 kDa-
20 kDa-
ctr
4h
8h
12h
24h
48h
72h

3D11-2 T-47D _CCM3

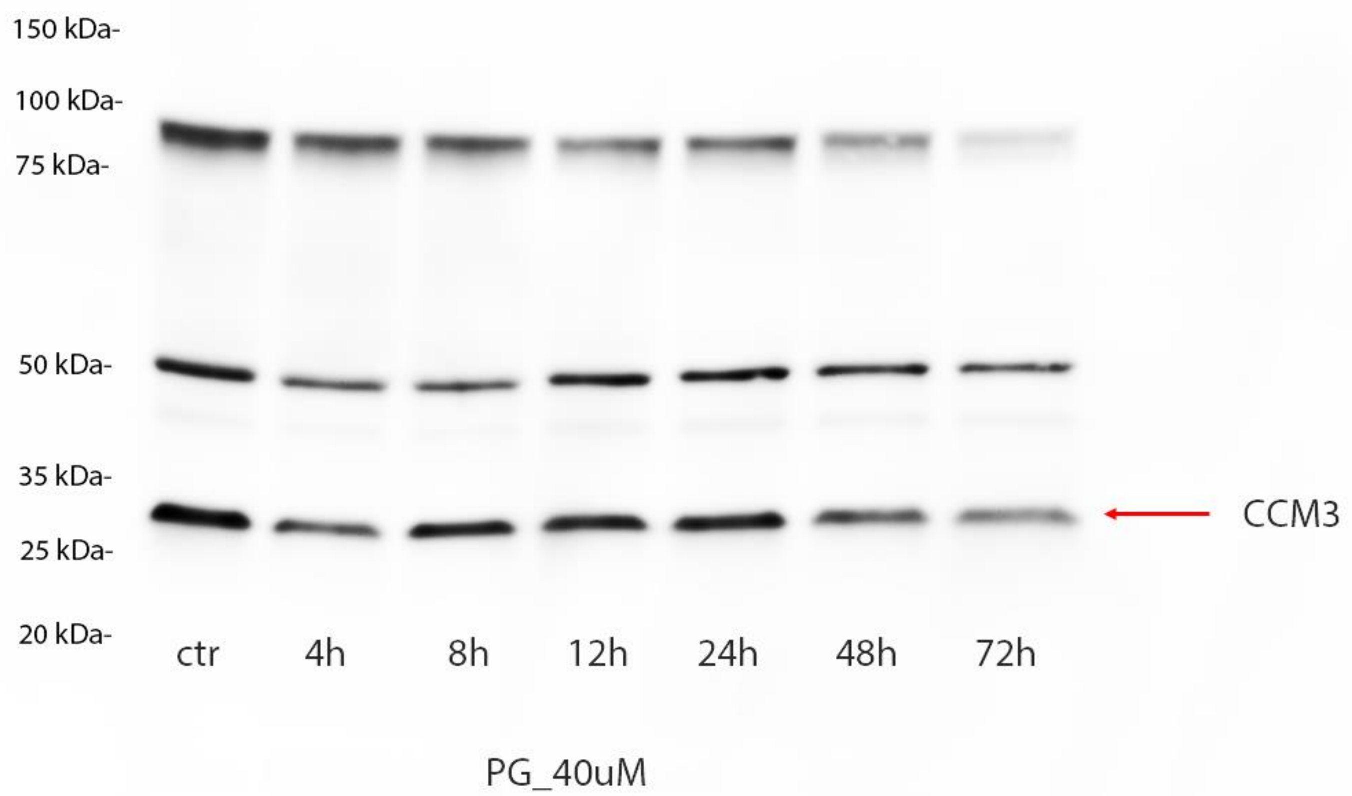

3D11-3

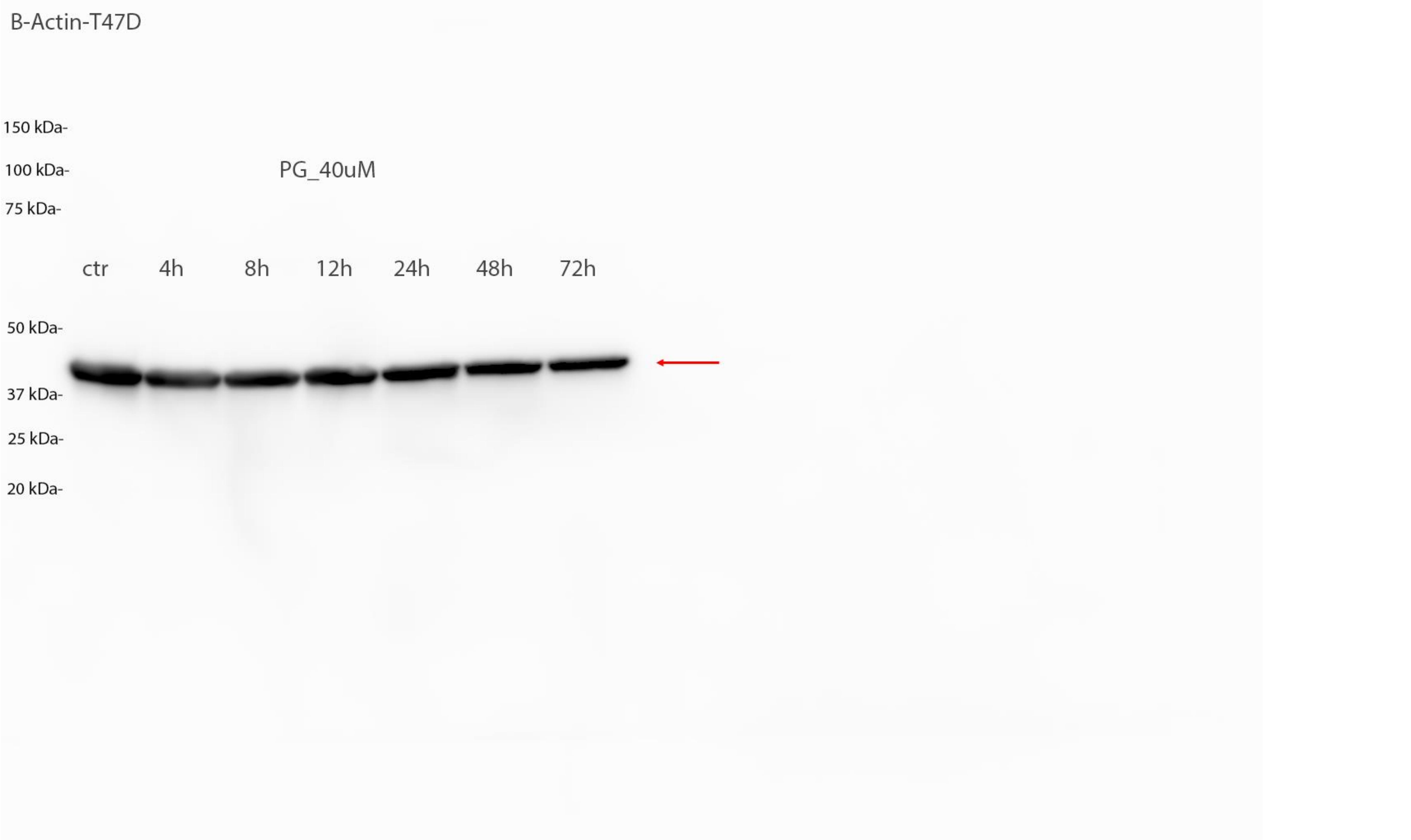
B-Actin-T47D
150 kDa-
100 kDa-
75 kDa-
PG_40uM
ctr
4h
8h
12h
24h
48h
72h
50 kDa-
37 kDa-
25 kDa-
20 kDa-

3D12-1

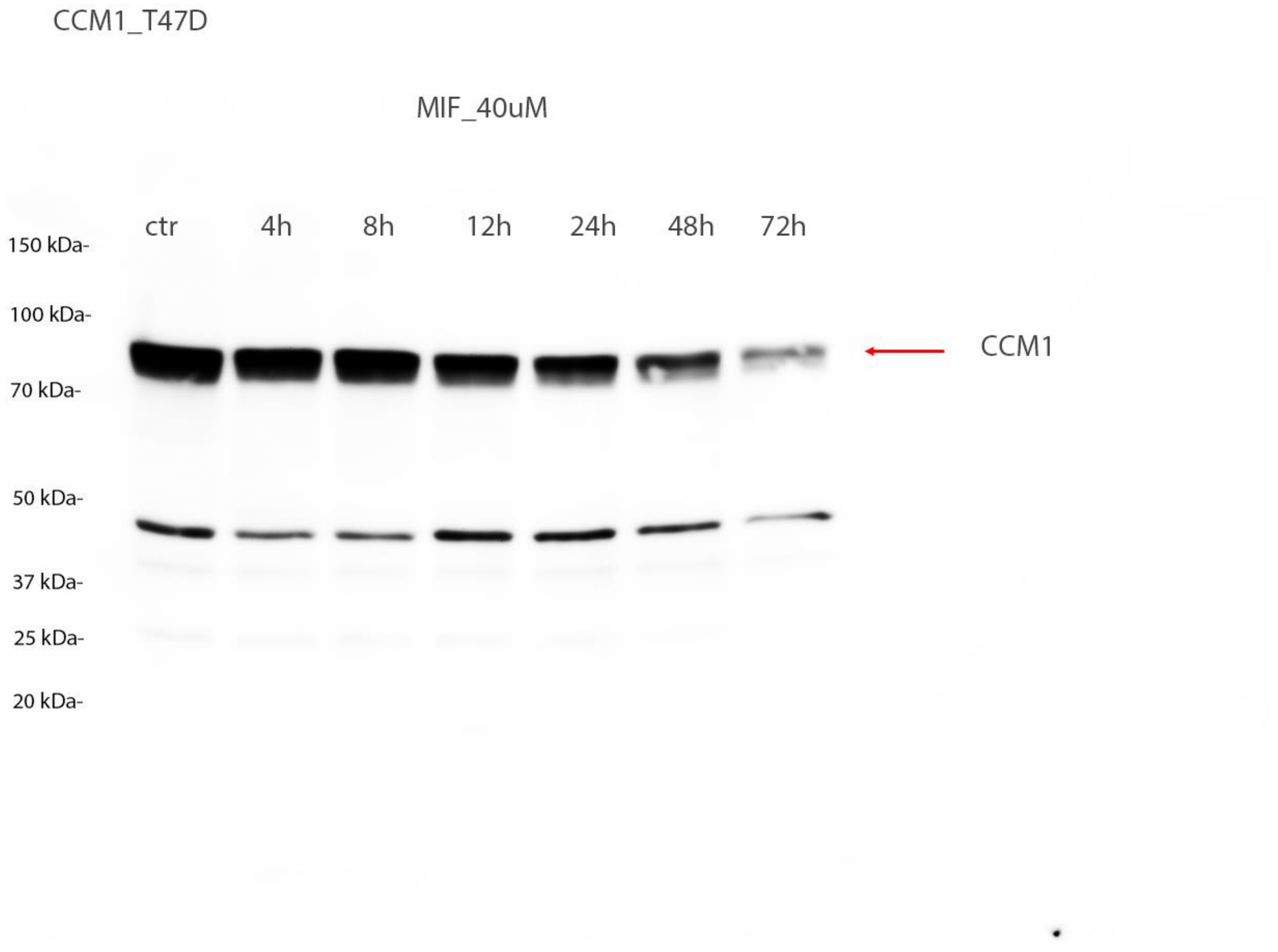
CCM1_T47D
MIF_40uM
ctr
4h
8h
12h
24h
48h
72h
150 kDa-
100 kDa-
70 kDa-
50 kDa-
37 kDa-
25 kDa-
20 kDa-
CCM1

3D12-2

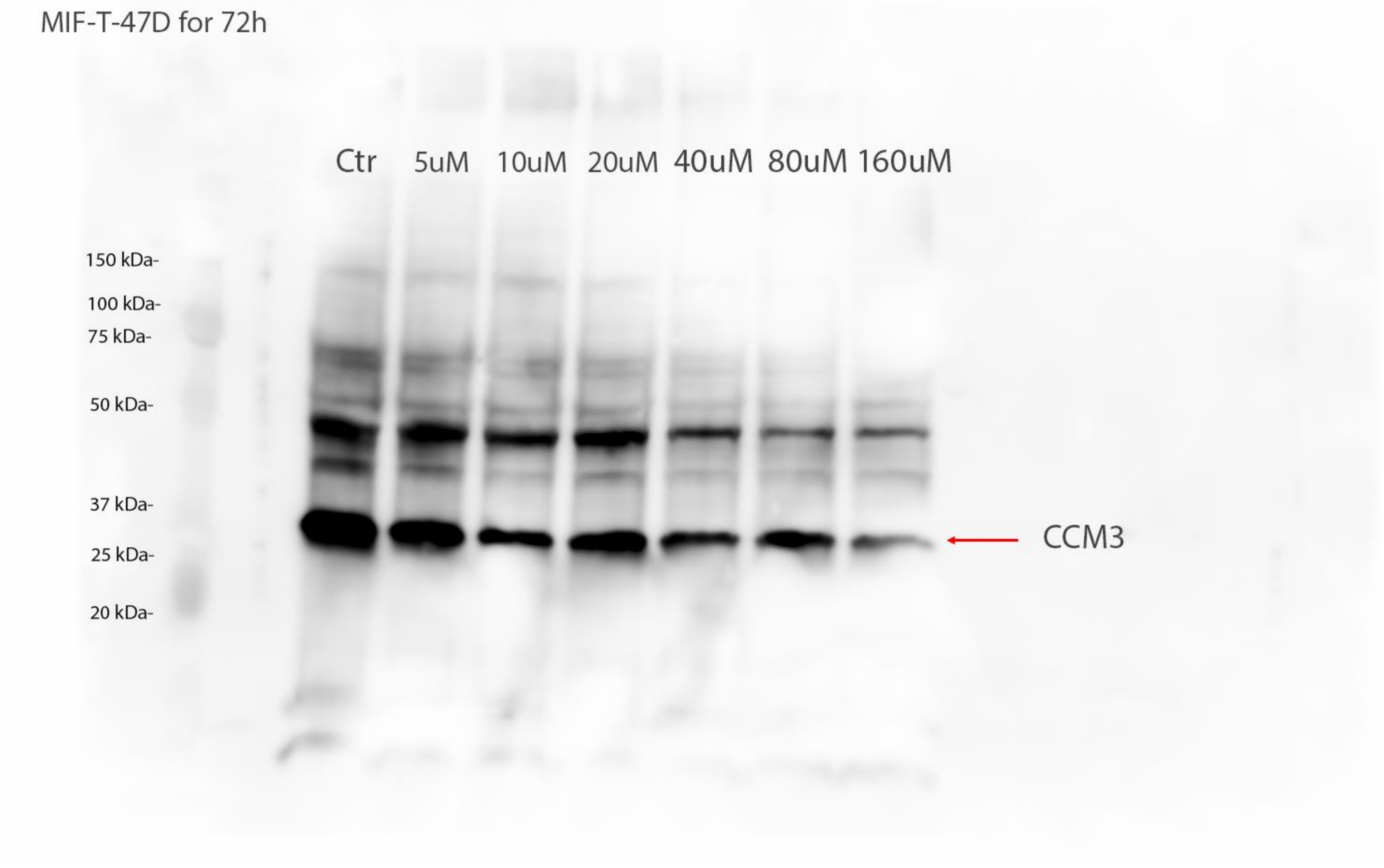
MIF-T-47D for 72h
Ctr
5uM
10uM
20uM
40uM
80uM
160uM
150 kDa-
100 kDa-
75 kDa-
50 kDa-
37 kDa-
25 kDa-
20 kDa-
CCM3

3D12-3

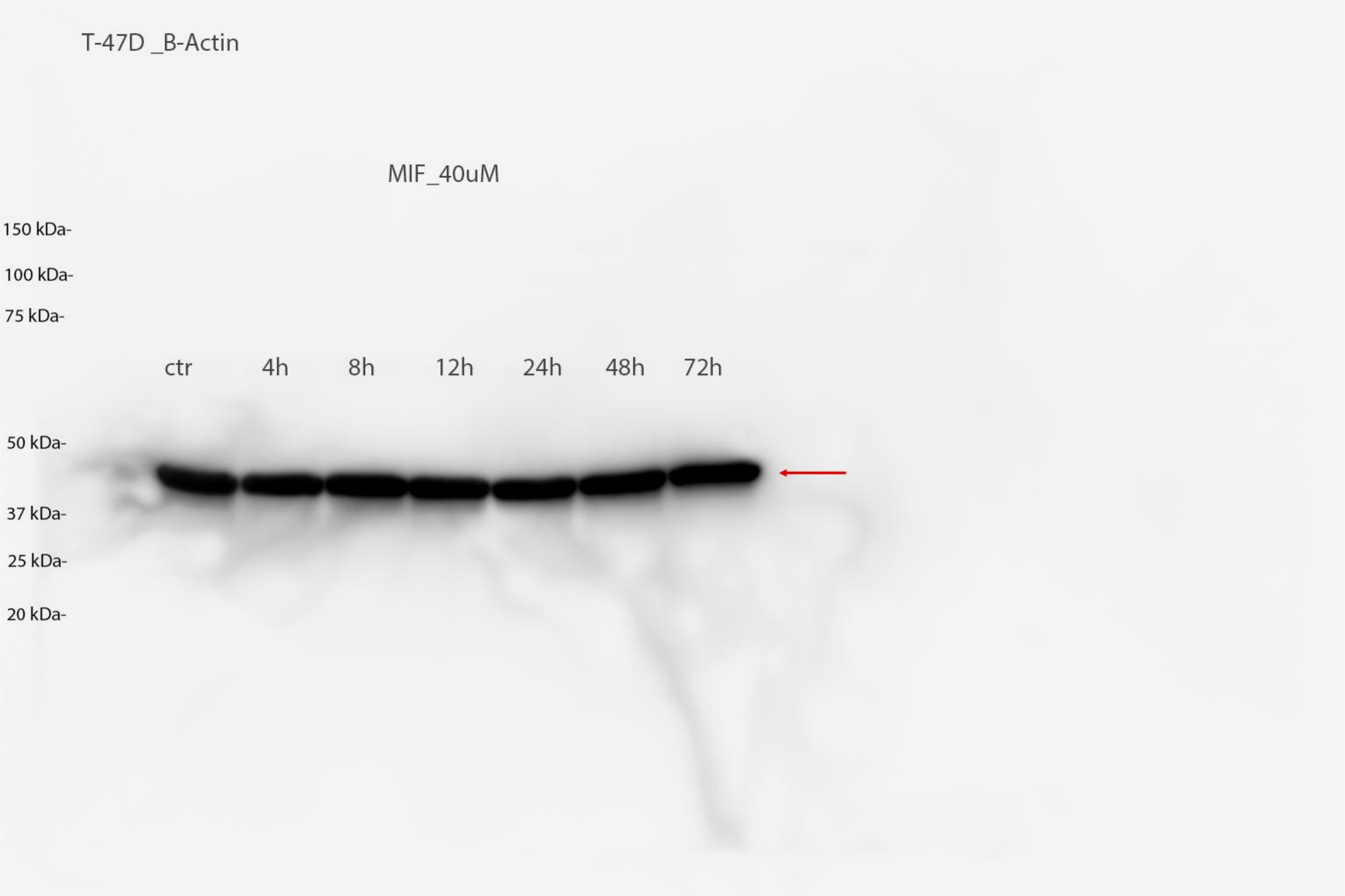
T-47D _B-Actin
MIF_40uM
150 kDa-
100 kDa-
75 kDa-
ctr
4h
8h
12h
24h
48h
72h
50 kDa-
37 kDa-
25 kDa-
20 kDa-

3D21-1

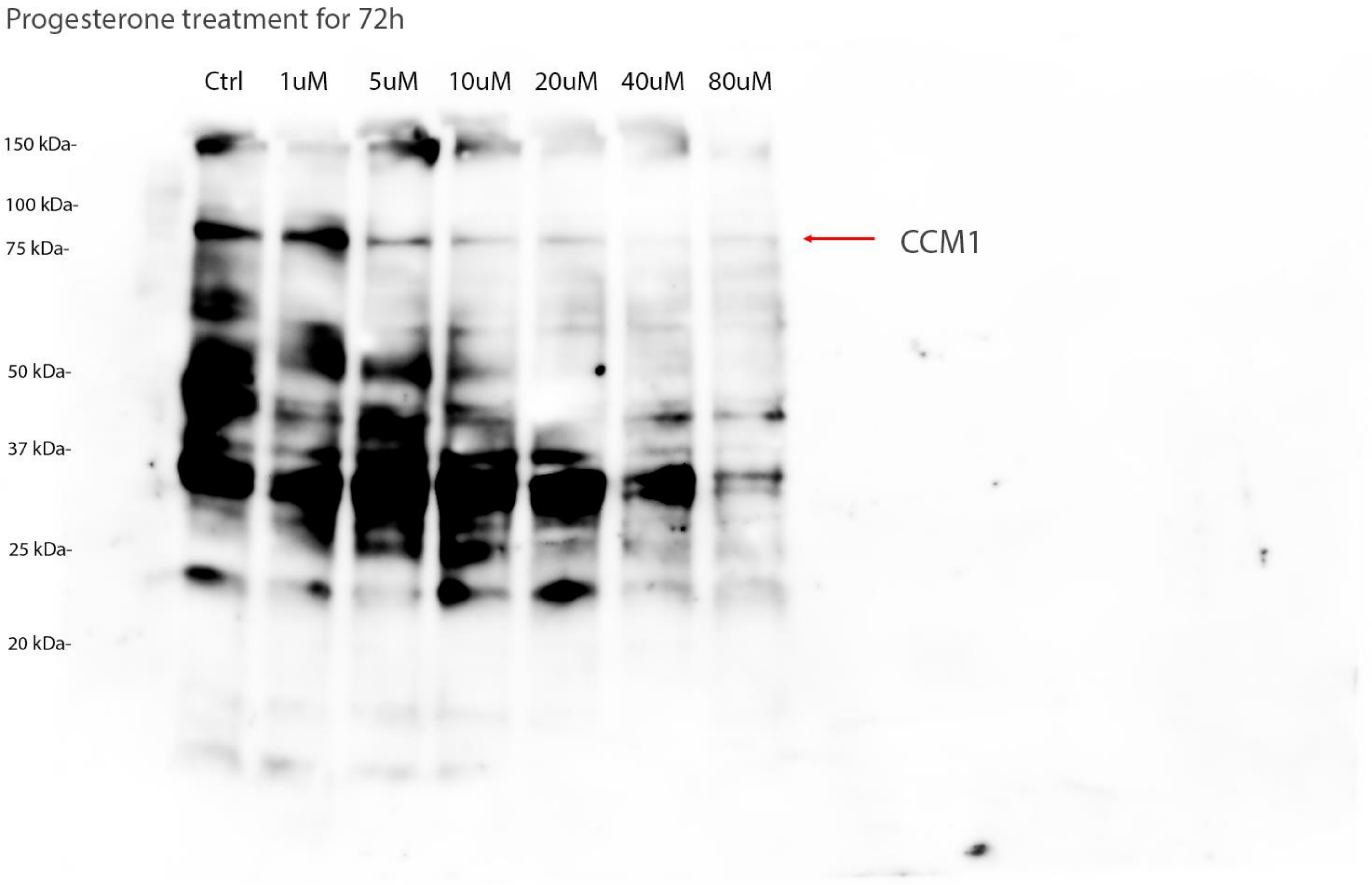
Progesterone treatment for 72h
Ctrl
1uM
5uM
10uM
20uM
40uM
80uM
150 kDa-
100 kDa-
75 kDa-
50 kDa-
37 kDa-
25 kDa-
20 kDa-
CCM1

3D21-2

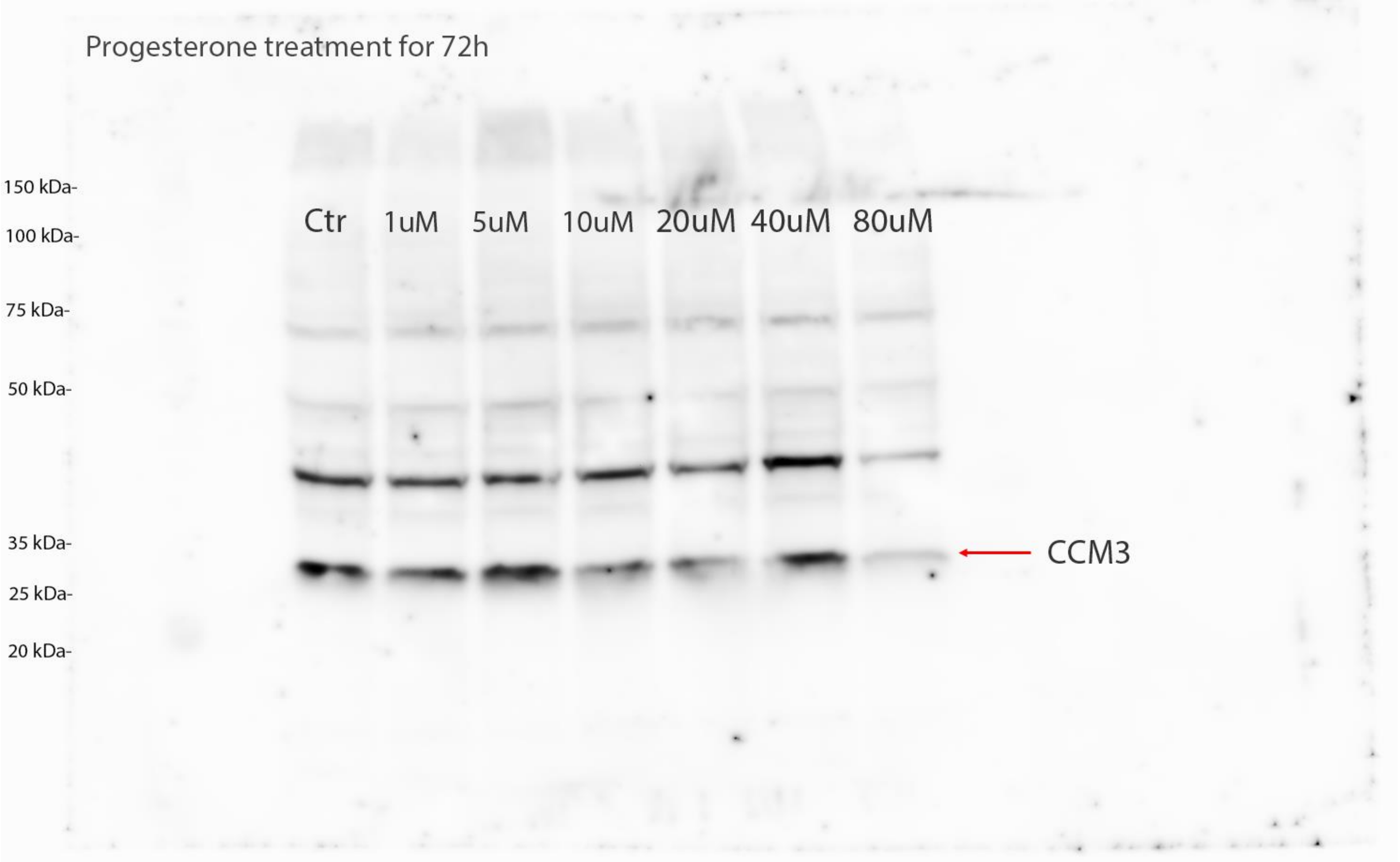

Progesterone treatment for 72h
150 kDa-
100 kDa-
75 kDa-
50 kDa-
35 kDa-
25 kDa-
20 kDa-
Ctr
1uM
5uM
10uM
20uM
40uM
80uM
CCM3

3D21-3

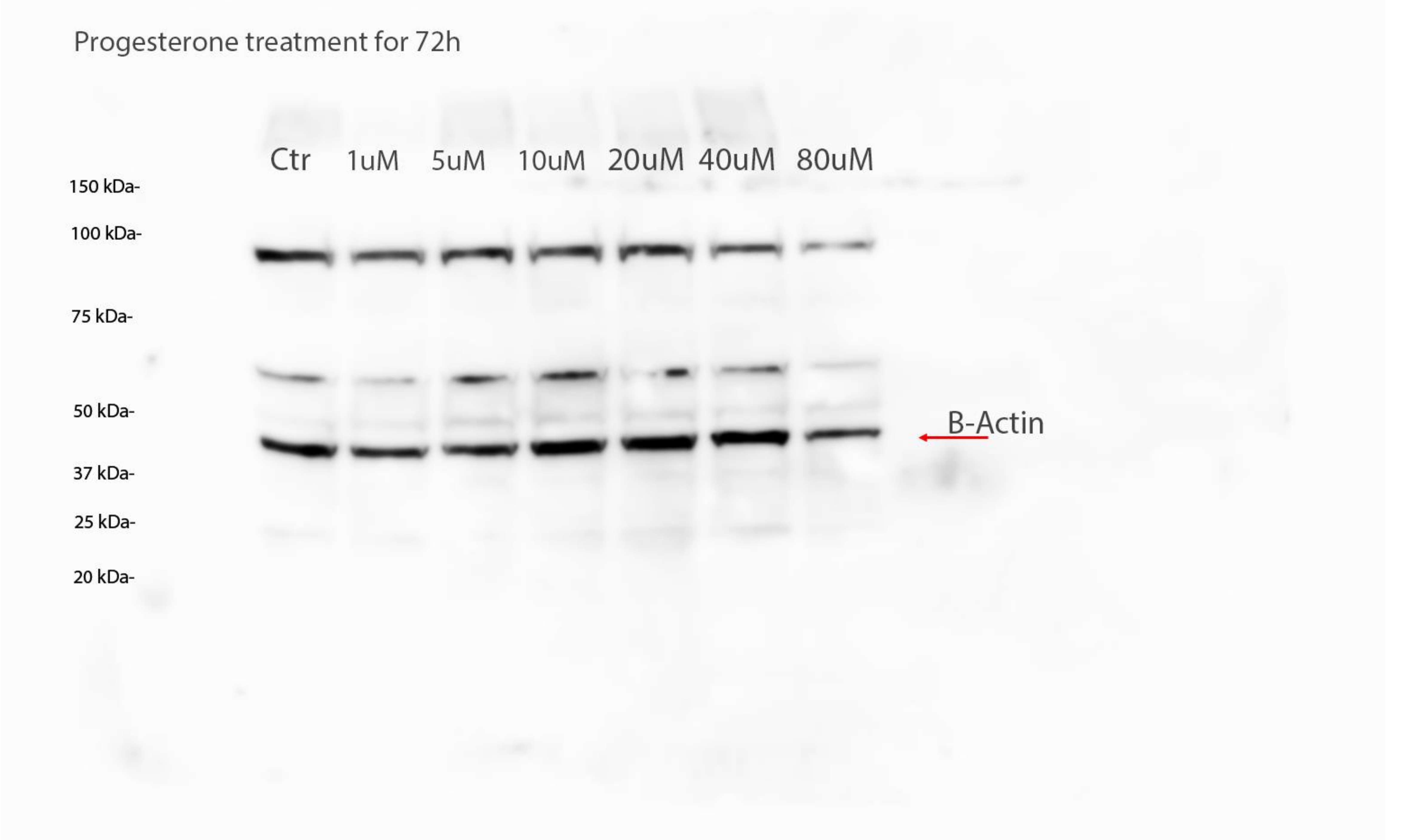

Progesterone treatment for 72h
Ctr
1uM
5uM
10uM
20uM
40uM
80uM
150 kDa-
100 kDa-
75 kDa-
50 kDa-
37 kDa-
25 kDa-
20 kDa-
B-Actin

3D22-1

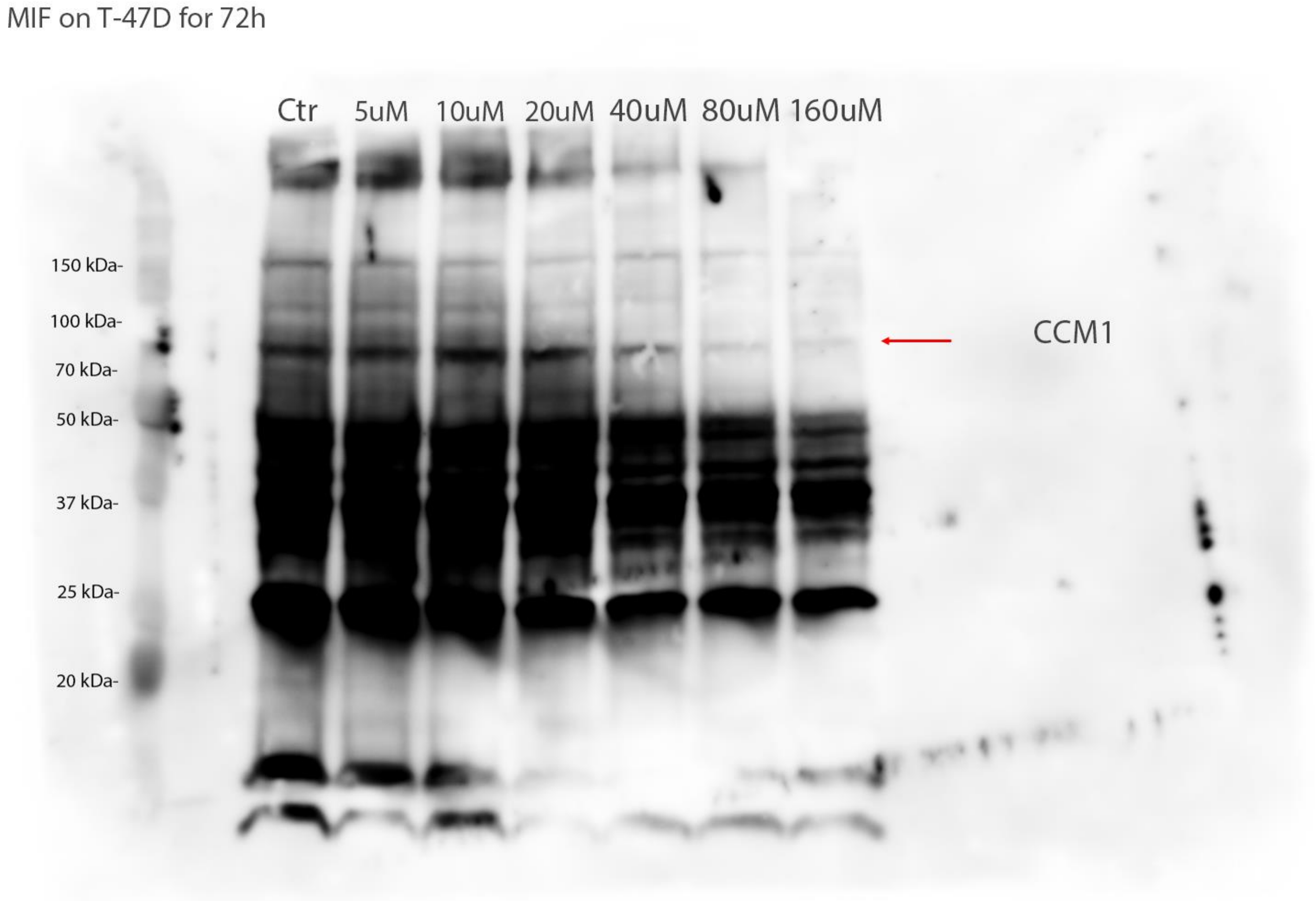
MIF on T-47D for 72h
Ctr
5uM
10uM
20uM
40uM
80uM
160uM
150 kDa-
100 kDa-
70 kDa-
50 kDa-
37 kDa-
25 kDa-
20 kDa-
CCM1

3D22-2

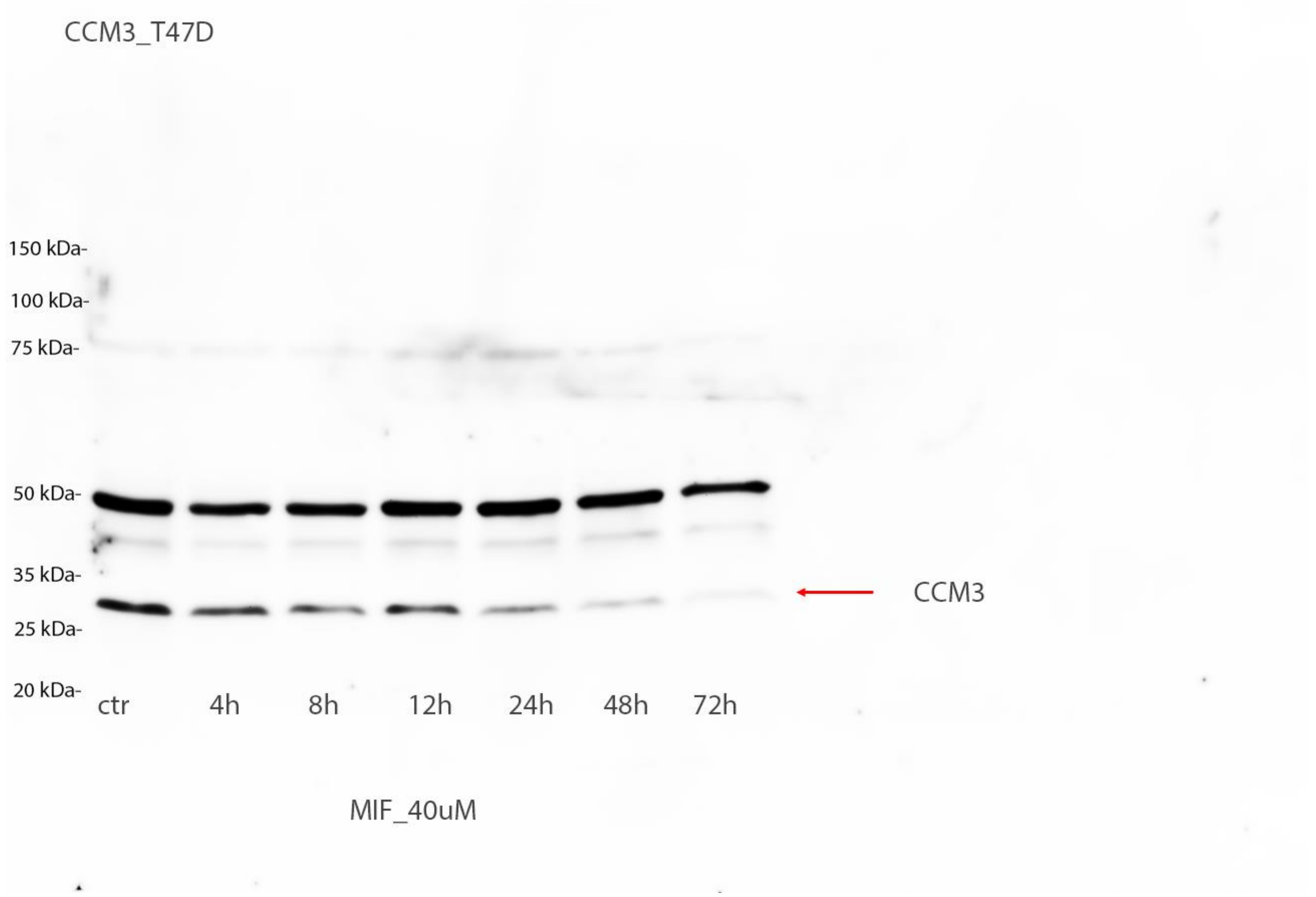
CCM3_T47D
150 kDa-
100 kDa-
75 kDa-
50 kDa-
35 kDa-
25 kDa-
20 kDa-
ctr
4h
8h
12h
24h
48h
72h
CCM3
MIF_40uM

3D22-3

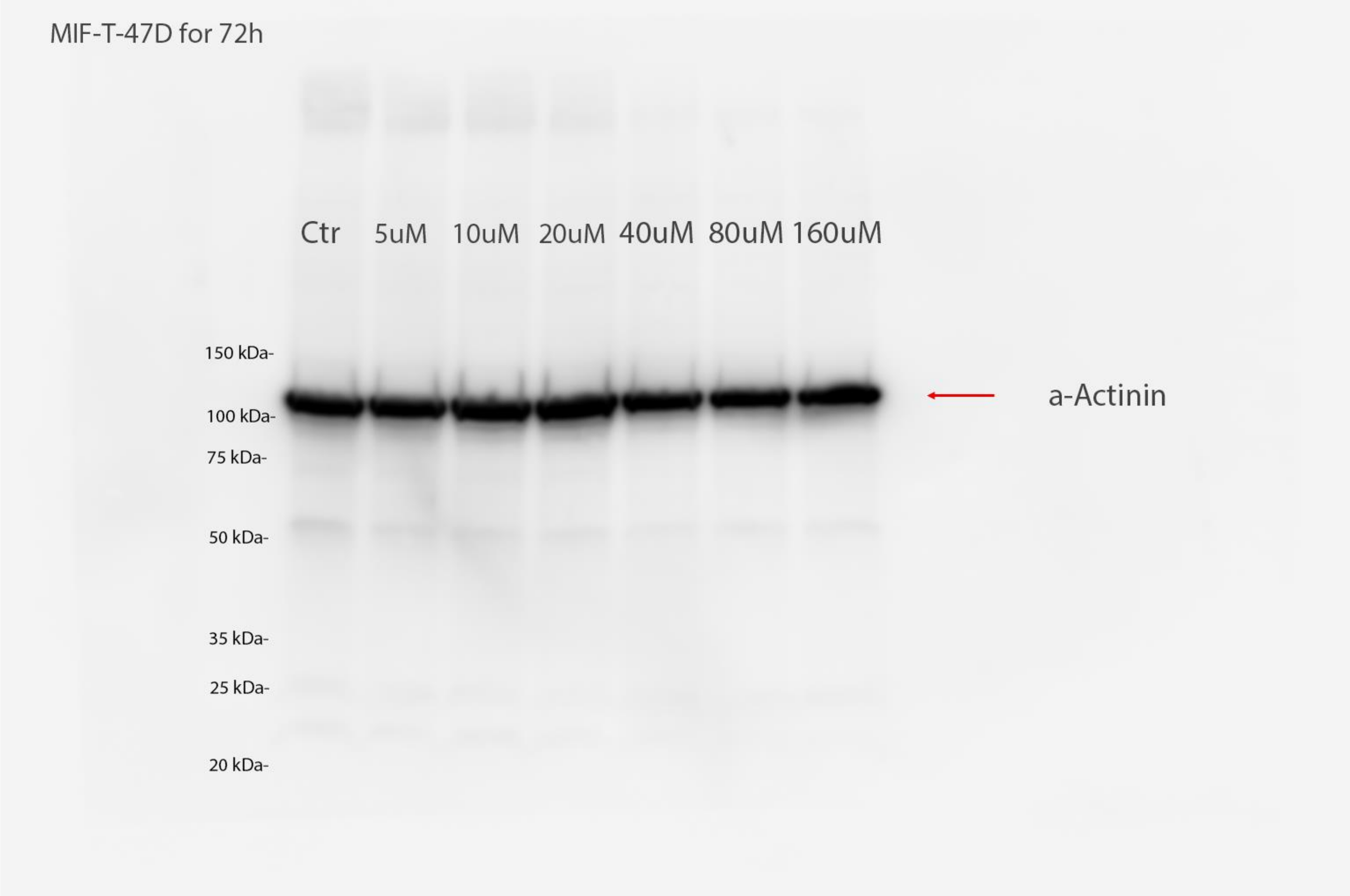
MIF-T-47D for 72h
Ctr
5uM
10uM
20uM
40uM
80uM
160uM
150 kDa-
100 kDa-
75 kDa-
50 kDa-
35 kDa-
25 kDa-
20 kDa-
a-Actinin

3G1-1

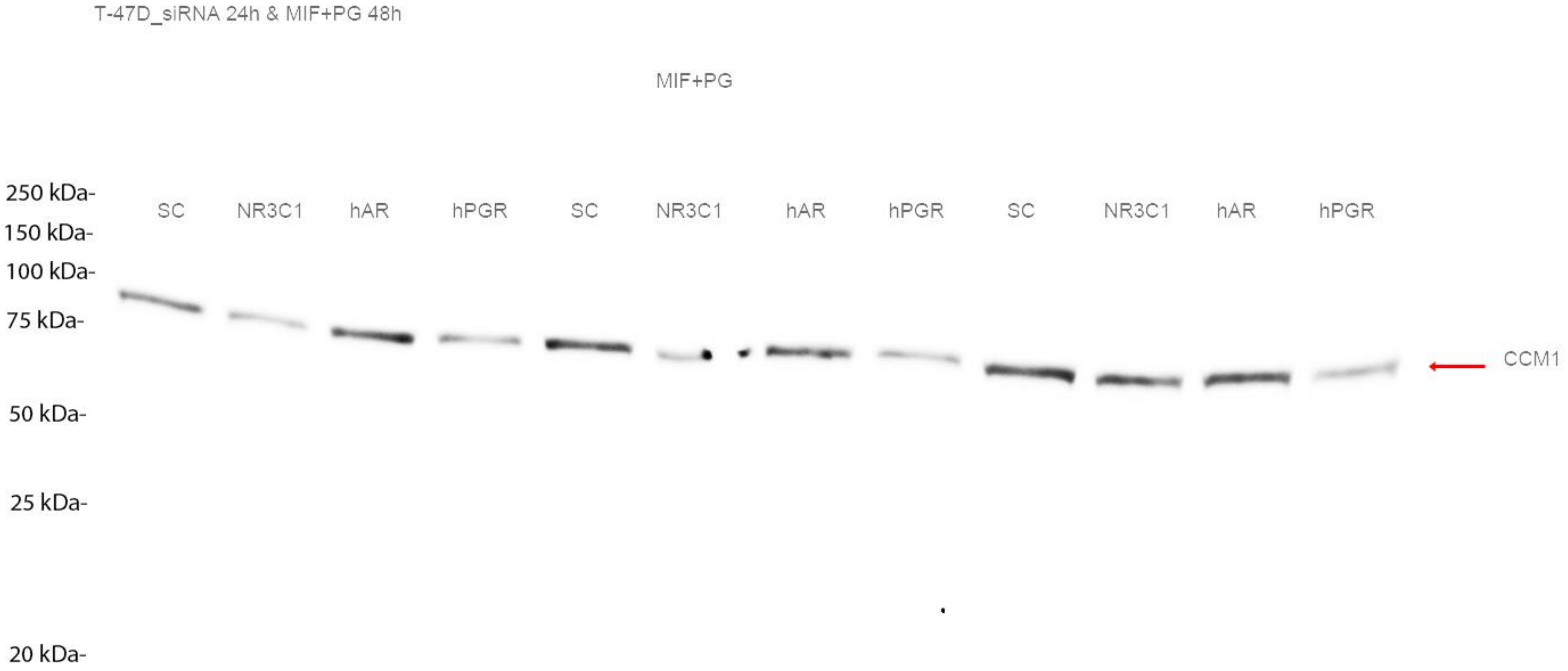
T-47D_siRNA 24h & MIF+PG 48h
MIF+PG
250 kDa-
150 kDa-
100 kDa-
75 kDa-
50 kDa-
25 kDa-
20 kDa-
SC
NR3C1
hAR
hPGR
SC
NR3C1
hAR
hPGR
SC
NR3C1
hAR
hPGR
CCM1

3G1-2

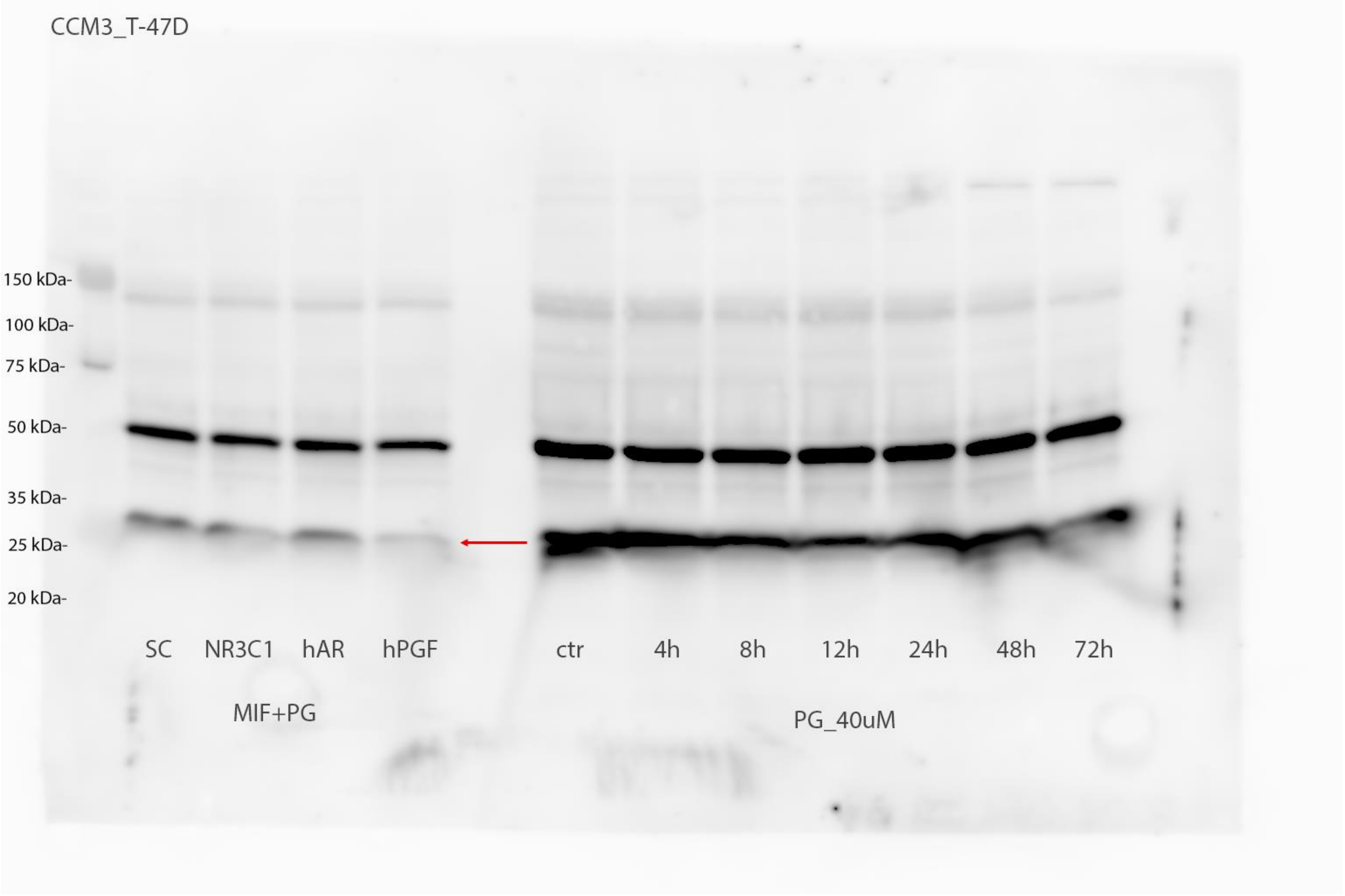

CCM3_T-47D
150 kDa-
100 kDa-
75 kDa-
50 kDa-
35 kDa-
25 kDa-
20 kDa-
SC
NR3C1
hAR
hPGF
ctr
4h
8h
12h
24h
48h
72h
MIF+PG
PG_40uM

3G1-3

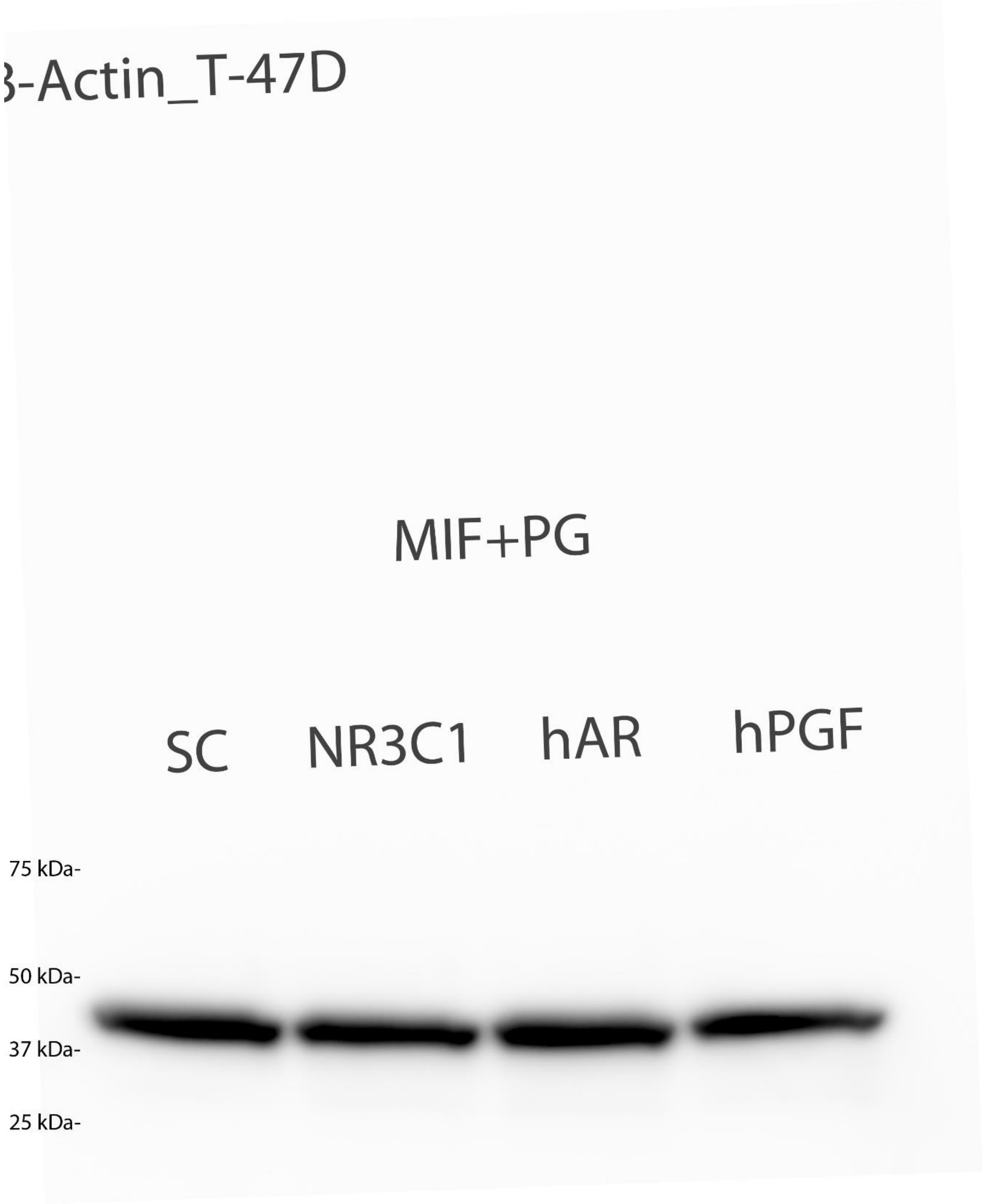

4B1-1

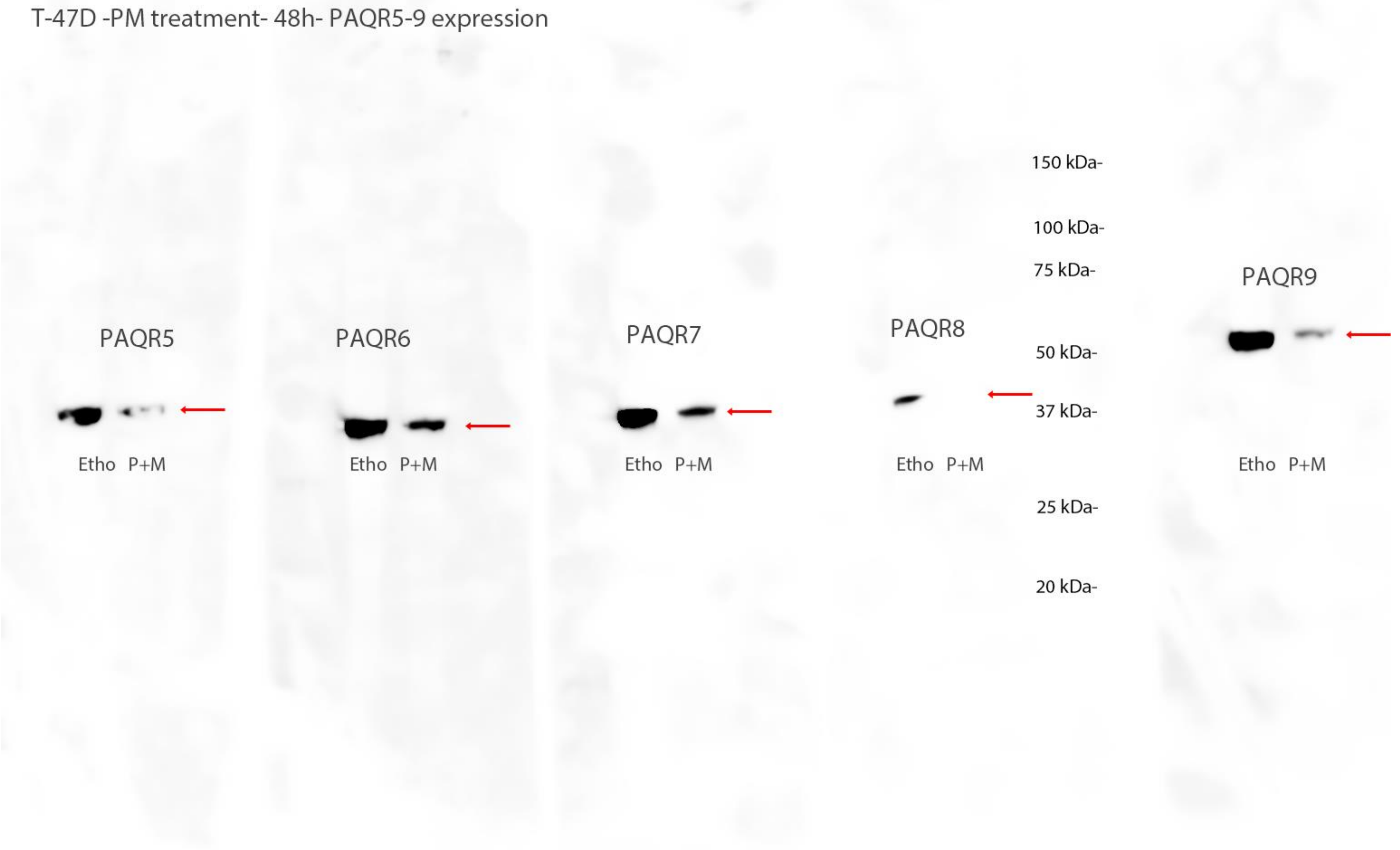

T-47D -PM treatment- 48h- PAQR5-9 expression
PAQR5
PAQR6
PAQR7
PAQR8
PAQR9
150 kDa-
100 kDa-
75 kDa-
50 kDa-
37 kDa-
25 kDa-
20 kDa-
Etho P+M
Etho P+M
Etho P+M
Etho P+M
Etho P+M

4B1-2

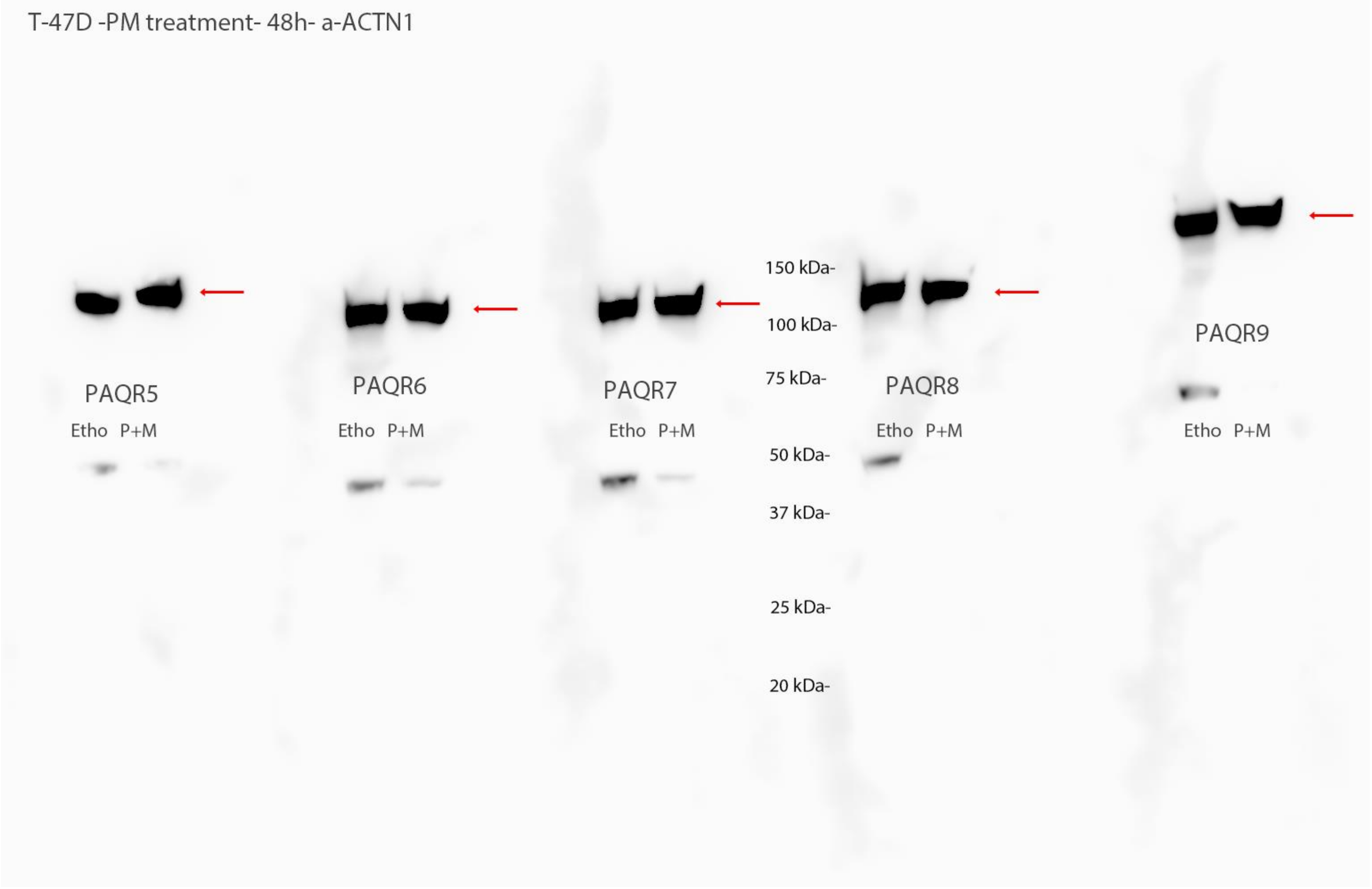
T-47D -PM treatment- 48h- a-ACTN1
150 kDa-
100 kDa-
75 kDa-
50 kDa-
37 kDa-
25 kDa-
20 kDa-
PAQR5
PAQR6
PAQR7
PAQR8
PAQR9
Etho P+M
Etho P+M
Etho P+M
Etho P+M
Etho P+M

4B2-1

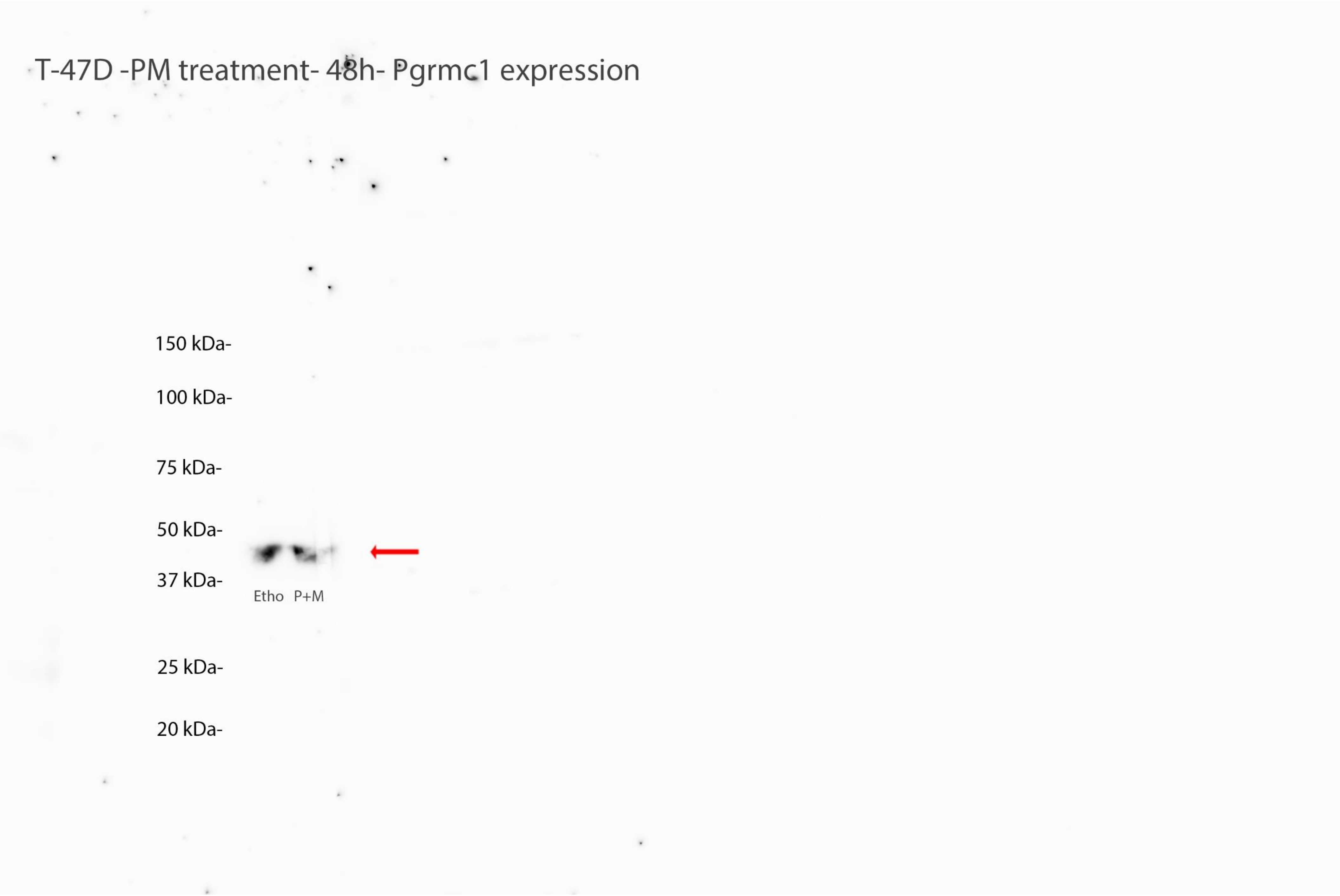
T-47D -PM treatment- 48h- Pgrmc1 expression
150 kDa-
100 kDa-
75 kDa-
50 kDa-
37 kDa-
Etho
P+M
25 kDa-
20 kDa-

4B2-2

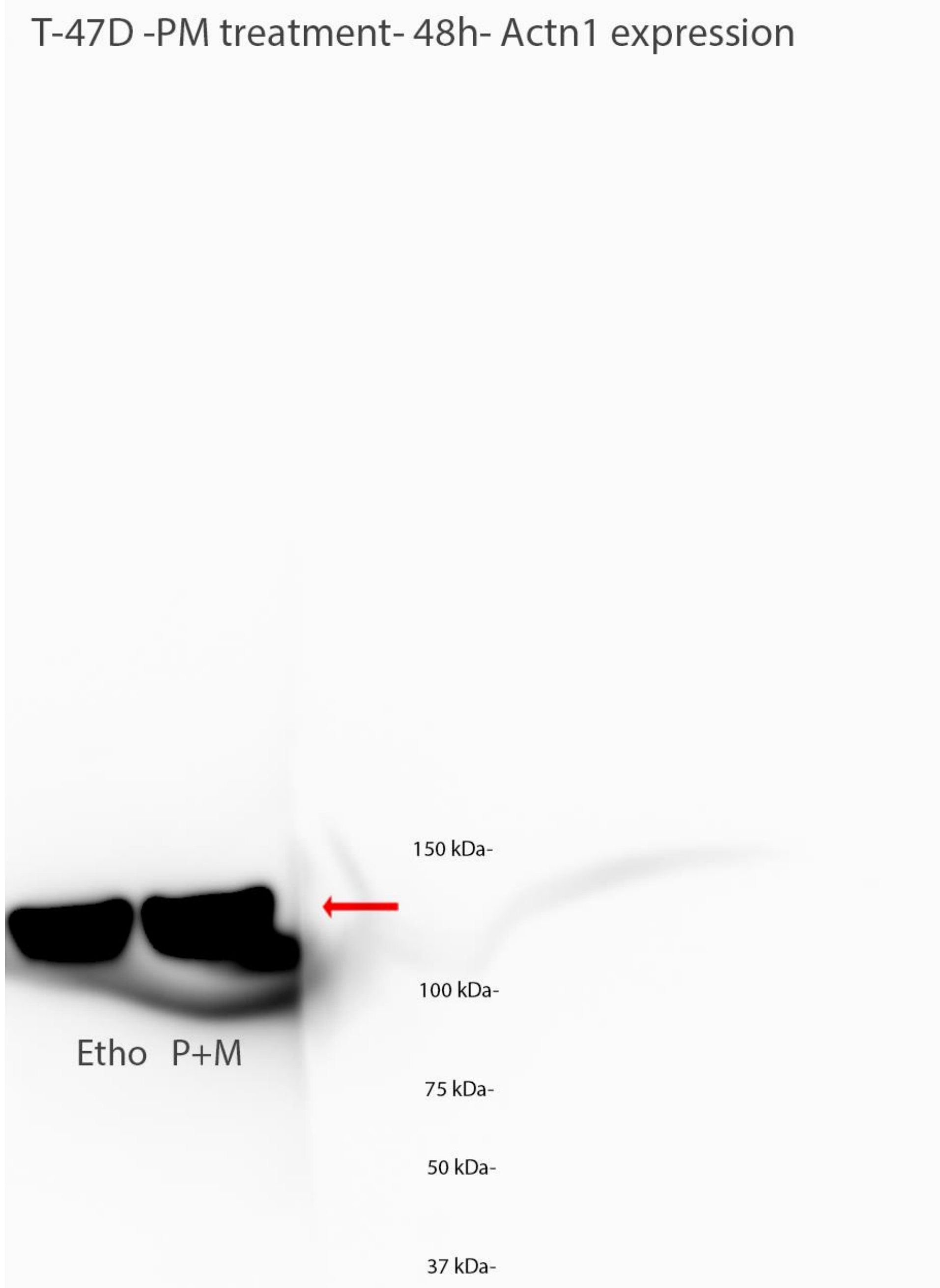
T-47D -PM treatment- 48h- Actn1 expression
150 kDa-
100 kDa-
75 kDa-
50 kDa-
37 kDa-
Etho
P+M

4C1-1

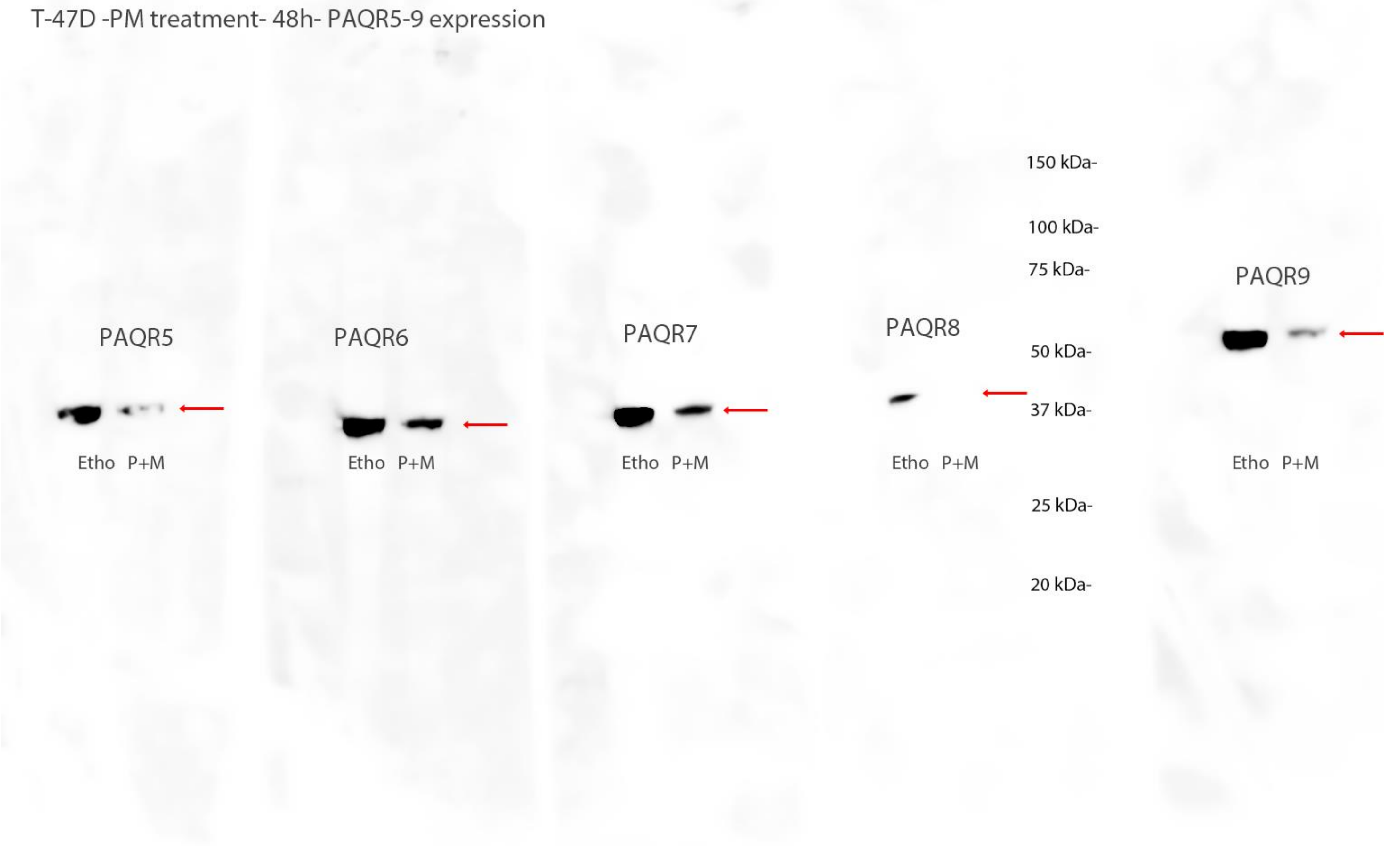
T-47D -PM treatment- 48h- PAQR5-9 expression
PAQR5
PAQR6
PAQR7
PAQR8
PAQR9
150 kDa-
100 kDa-
75 kDa-
50 kDa-
37 kDa-
25 kDa-
20 kDa-
Etho P+M
Etho P+M
Etho P+M
Etho P+M
Etho P+M

4C1-2

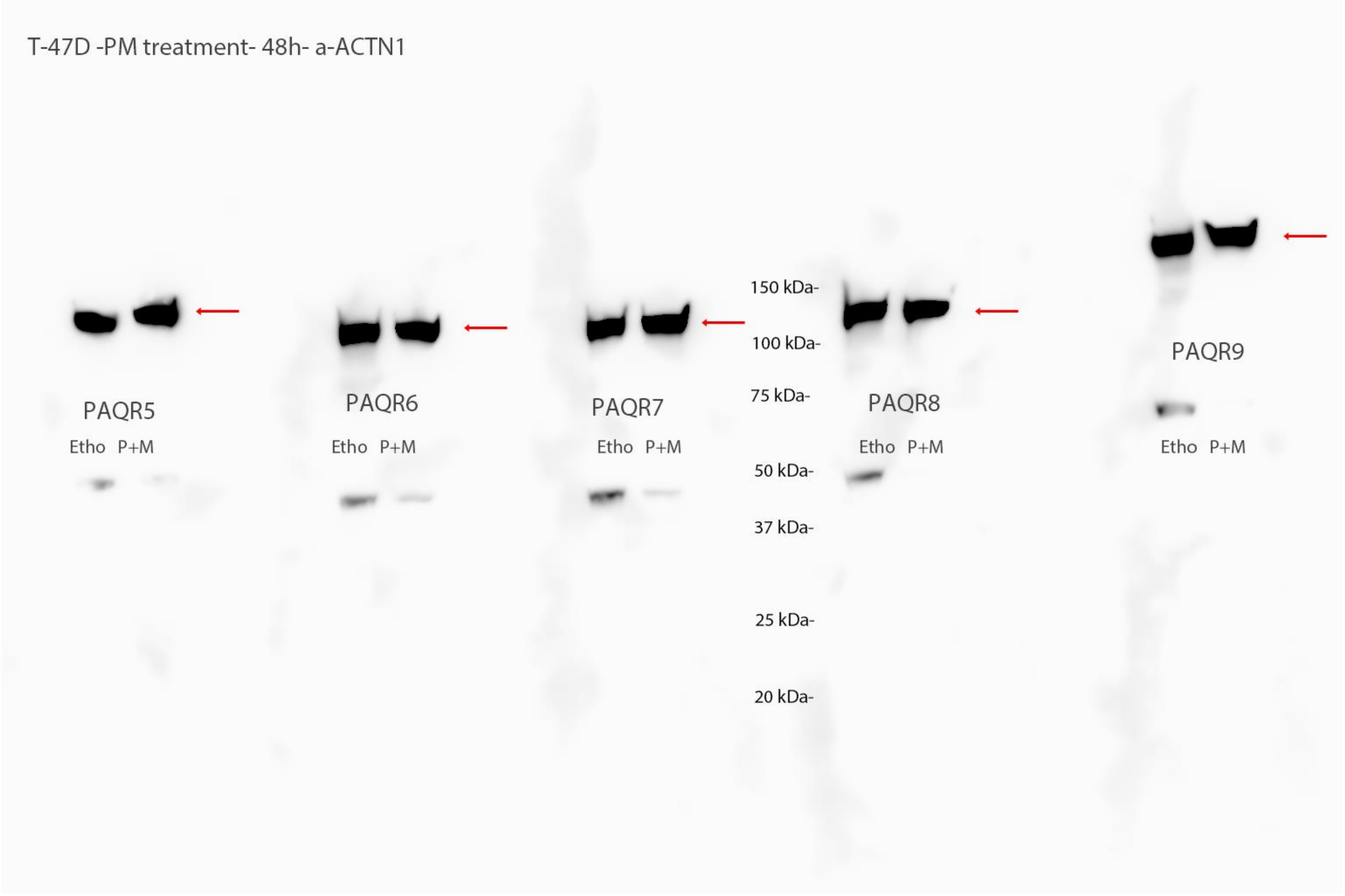
T-47D -PM treatment- 48h- a-ACTN1
150 kDa-
100 kDa-
75 kDa-
50 kDa-
37 kDa-
25 kDa-
20 kDa-
PAQR5
PAQR6
PAQR7
PAQR8
PAQR9
Etho P+M
Etho P+M
Etho P+M
Etho P+M
Etho P+M

Fig. 4G-1

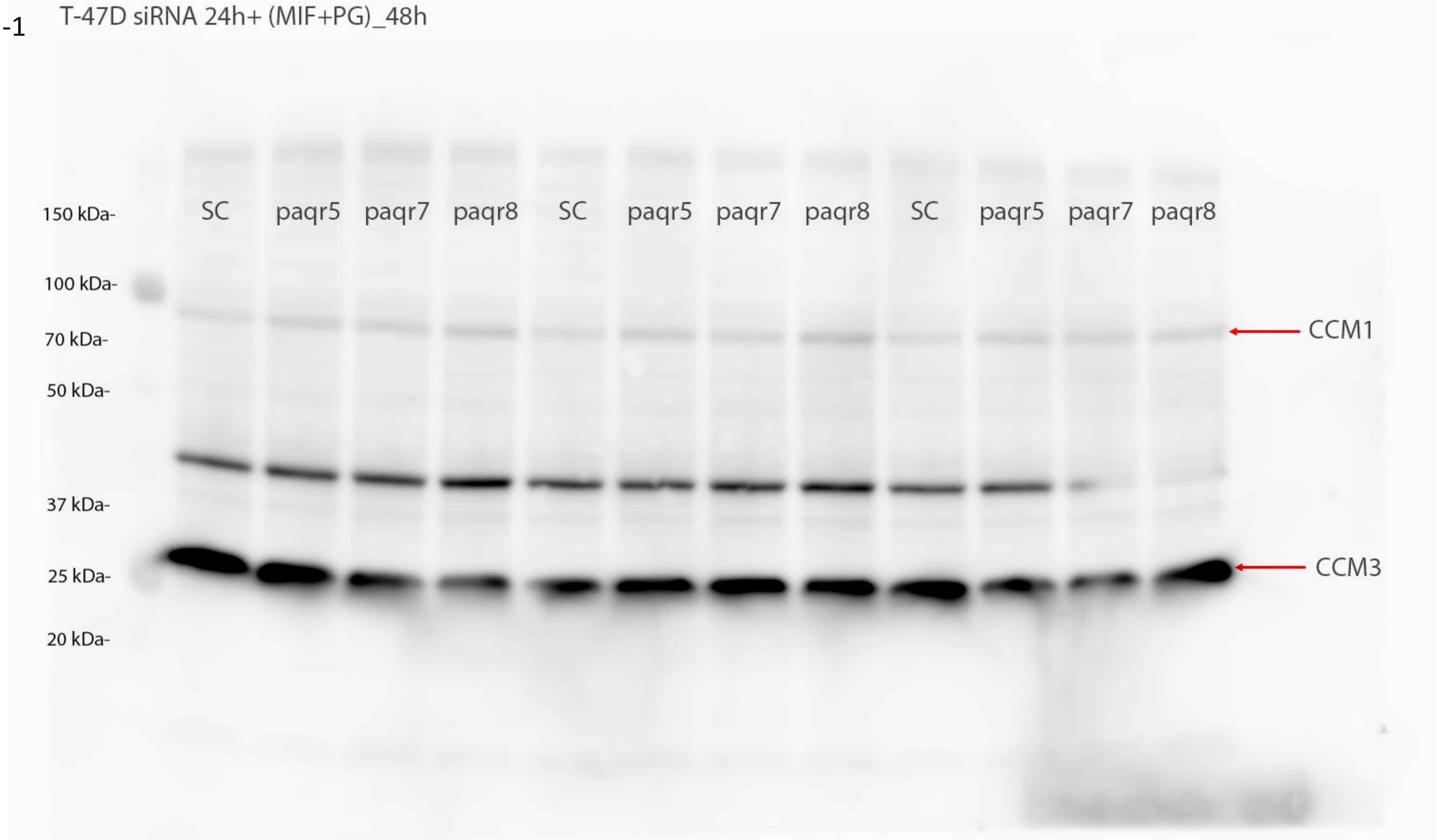

T-47D siRNA 24h+ (MIF+PG)_48h
150 kDa-
100 kDa-
70 kDa-
50 kDa-
37 kDa-
25 kDa-
20 kDa-
SC
paqr5
paqr7
paqr8
SC
paqr5
paqr7
paqr8
SC
paqr5
paqr7
paqr8
CCM1
CCM3

Fig. 4G-2

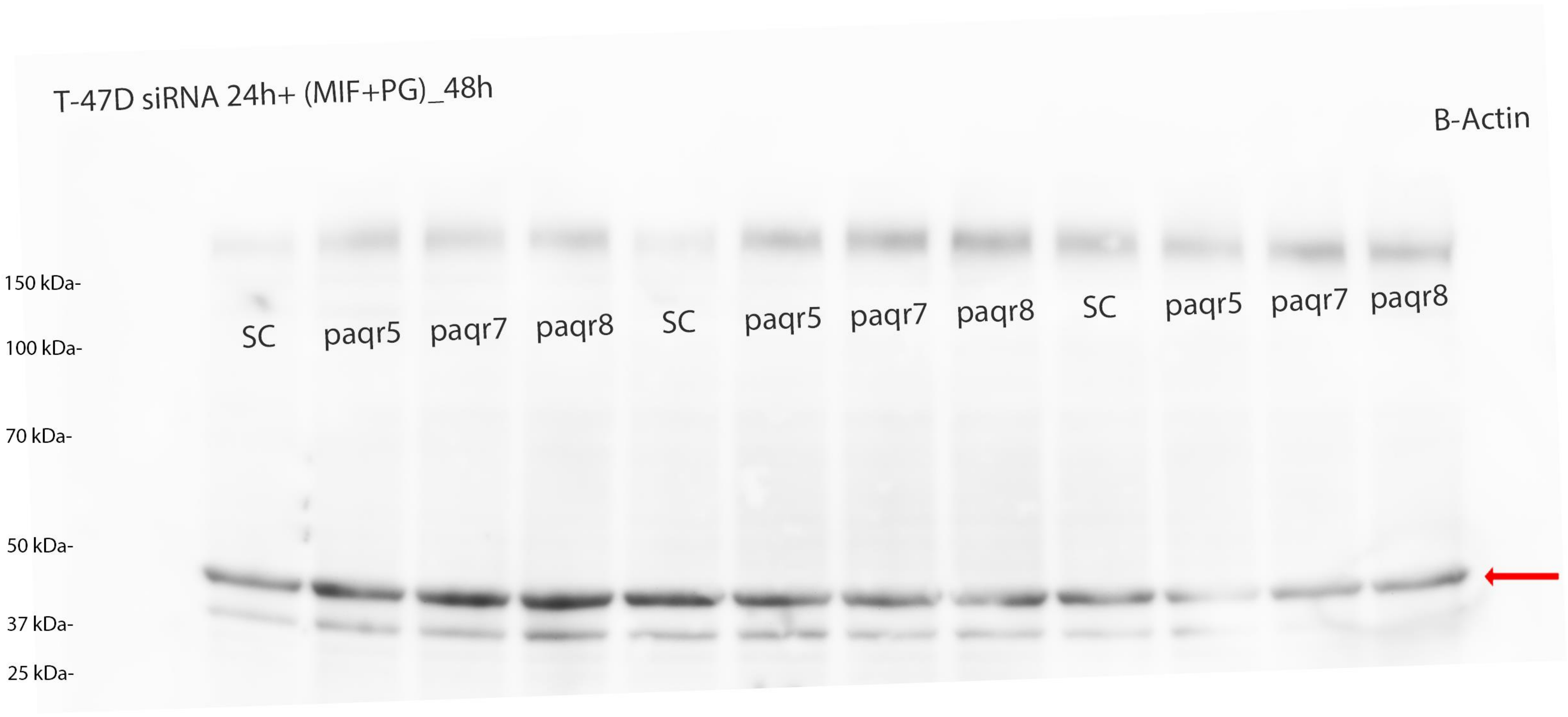
T-47D siRNA 24h+ (MIF+PG)_48h
B-Actin
150 kDa-
100 kDa-
70 kDa-
50 kDa-
37 kDa-
25 kDa-
SC
paqr5
paqr7
paqr8
SC
paqr5
paqr7
paqr8
SC
paqr5
paqr7
paqr8

5A1-1

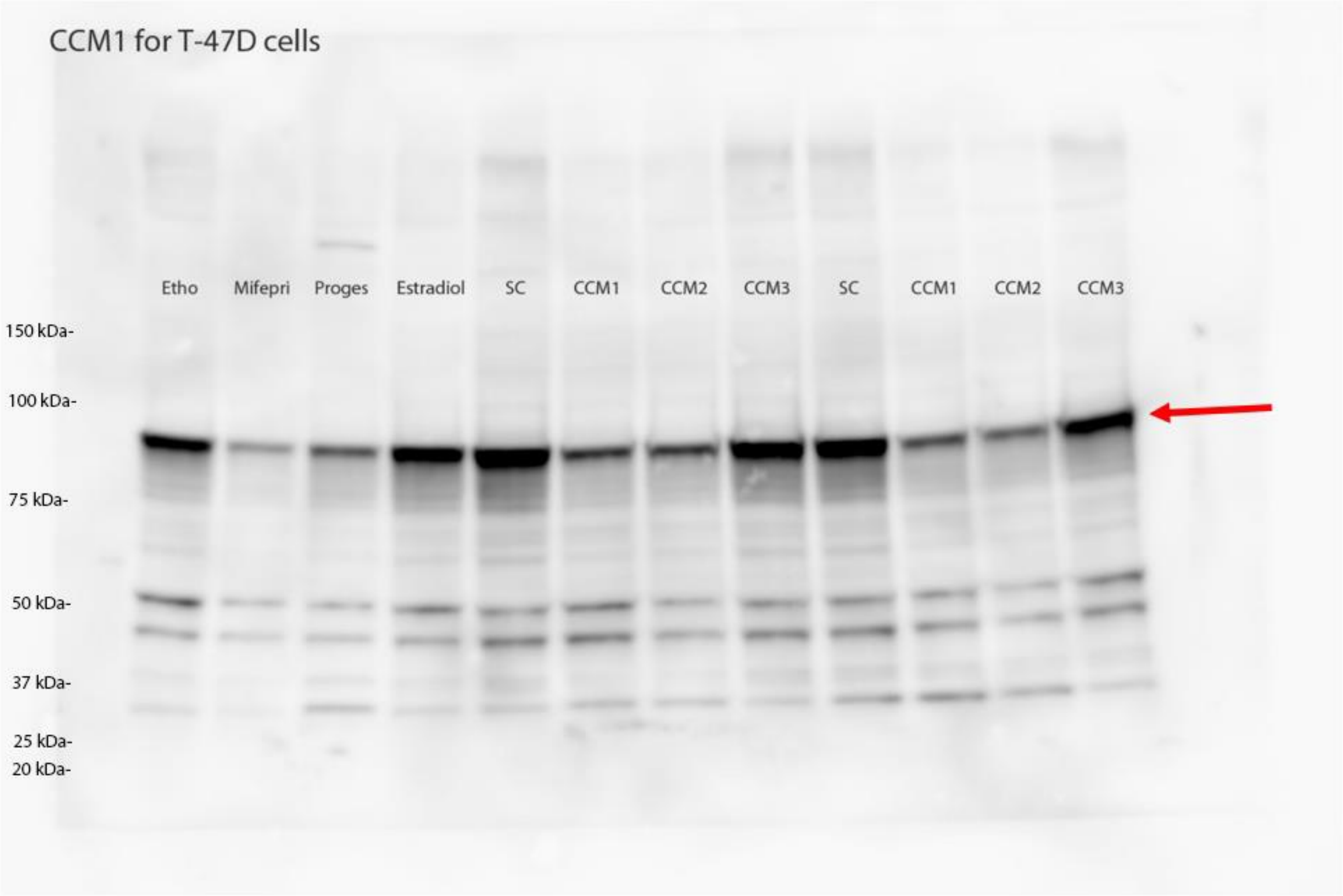
CCM1 for T-47D cells
Etho
Mifepri
Proges
Estradiol
SC
CCM1
CCM2
CCM3
SC
CCM1
CCM2
CCM3
150 kDa-
100 kDa-
75 kDa-
50 kDa-
37 kDa-
25 kDa-
20 kDa-

5A1-2

Wet transfer

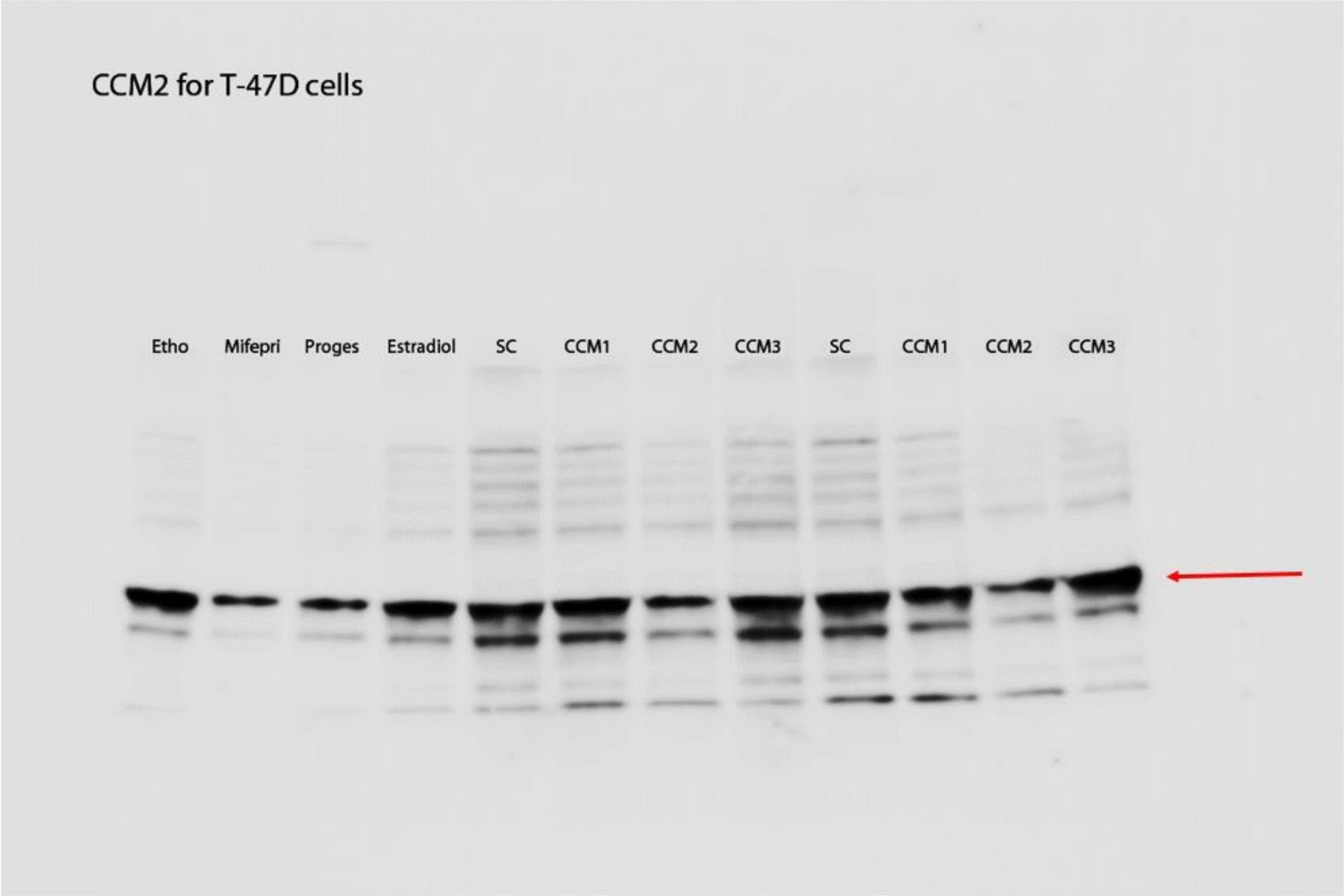

5A1-3

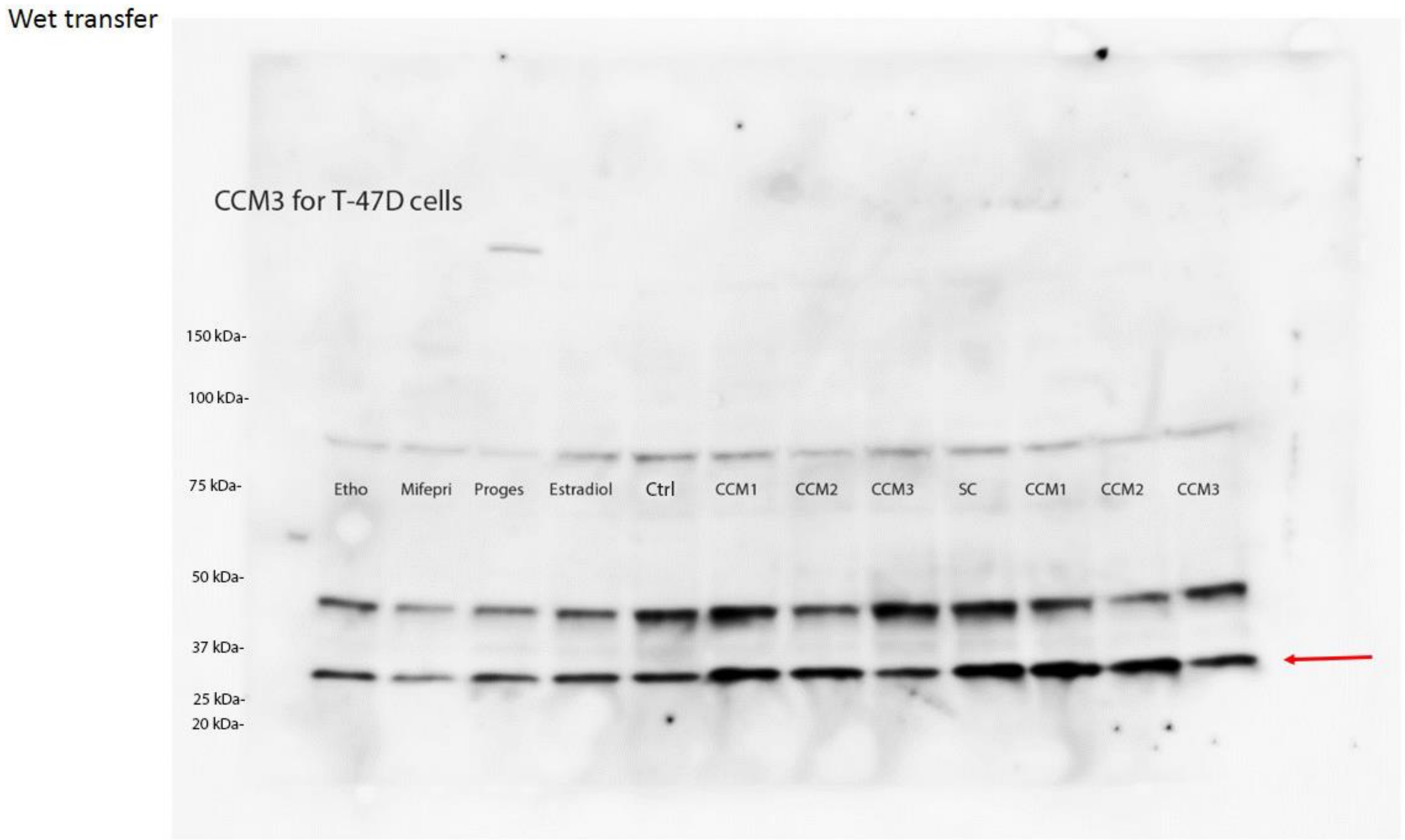
Wet transfer
CCM3 for T-47D cells
150 kDa-
100 kDa-
75 kDa-
50 kDa-
37 kDa-
25 kDa-
20 kDa-
Etho
Mifepri
Proges
Estradiol
Ctrl
CCM1
CCM2
CCM3
SC
CCM1
CCM2
CCM3

5A1-4

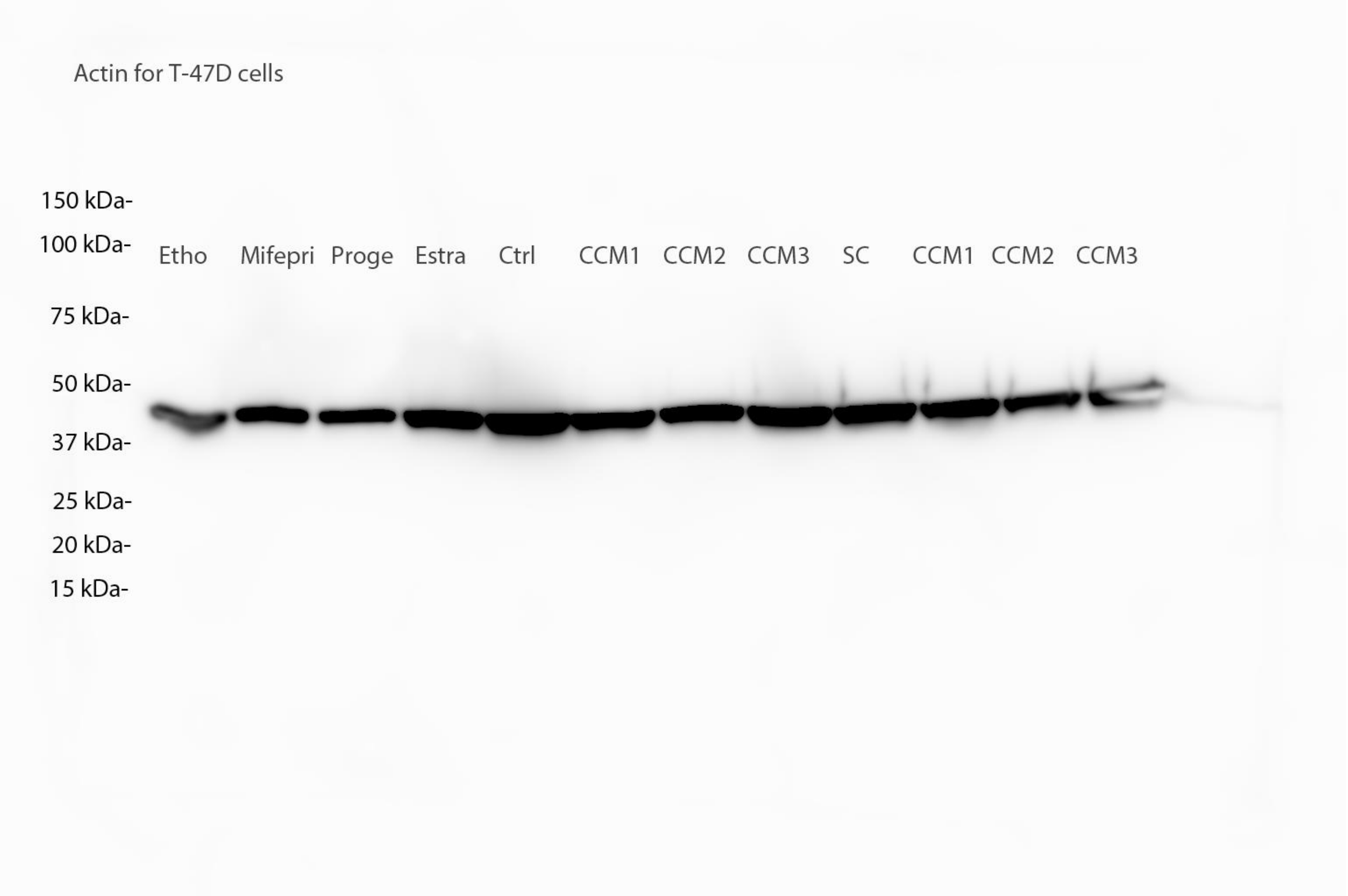
Actin for T-47D cells
150 kDa-
100 kDa-
75 kDa-
50 kDa-
37 kDa-
25 kDa-
20 kDa-
15 kDa-
Etho
Mifepri
Proge
Estra
Ctrl
CCM1
CCM2
CCM3
SC
CCM1
CCM2
CCM3

Suppl. 2-1

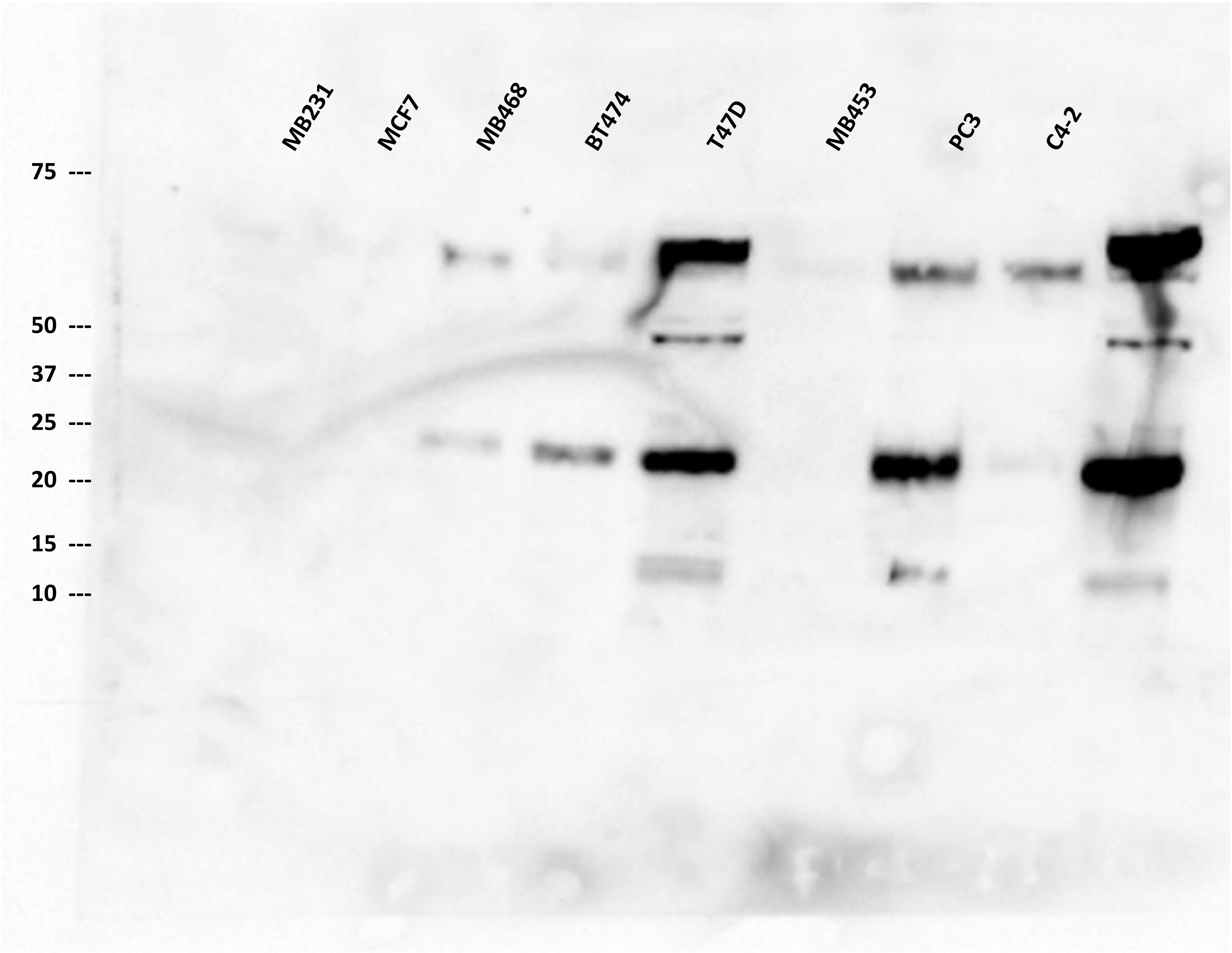

Suppl. 2-2

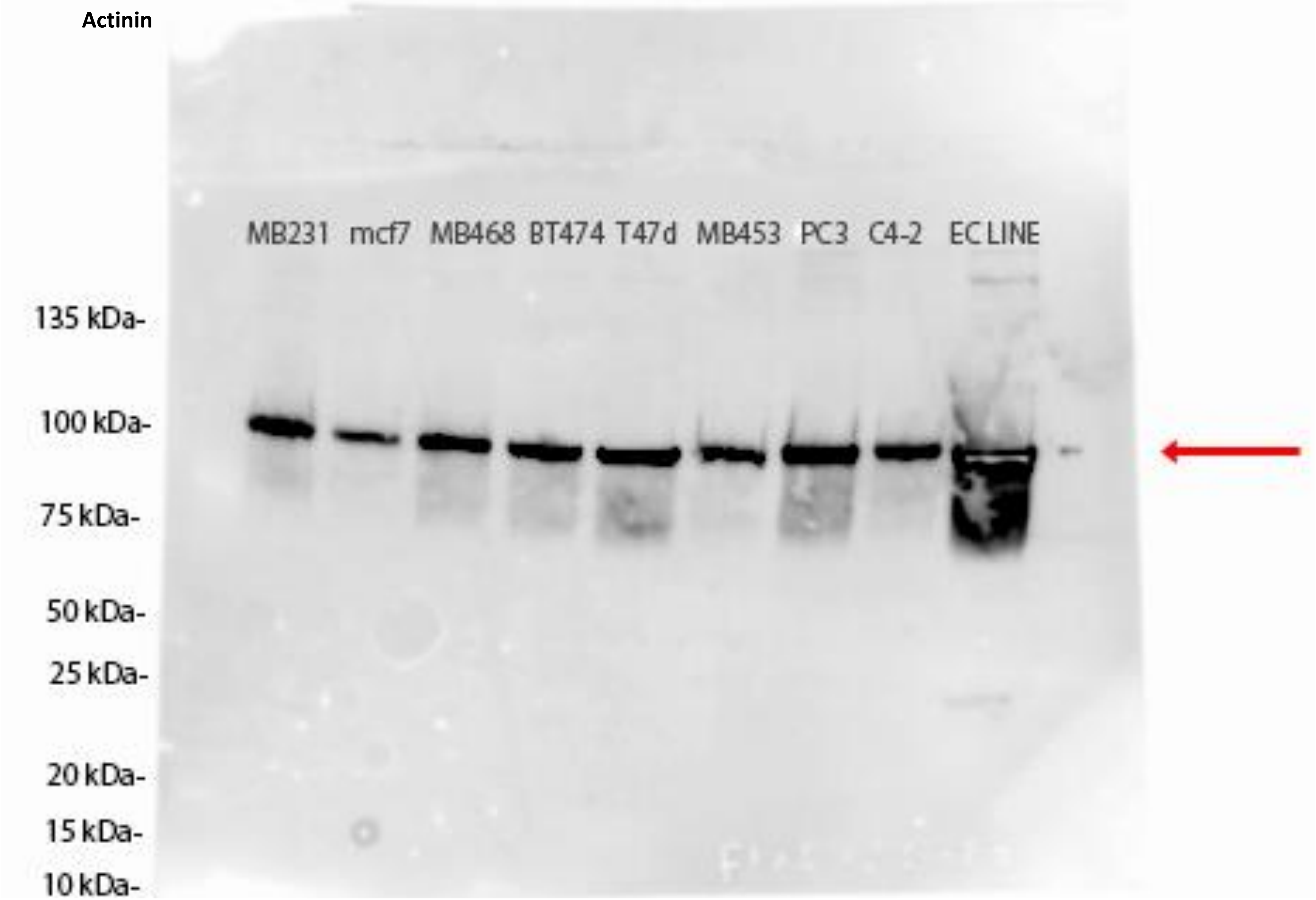
Actinin
MB231 mcf7 MB468 BT474 T47d MB453 PC3 C4-2 EC LINE
135 kDa-
100 kDa-
75 kDa-
50 kDa-
25 kDa-
20 kDa-
15 kDa-
10 kDa-

Suppl. 3A-1

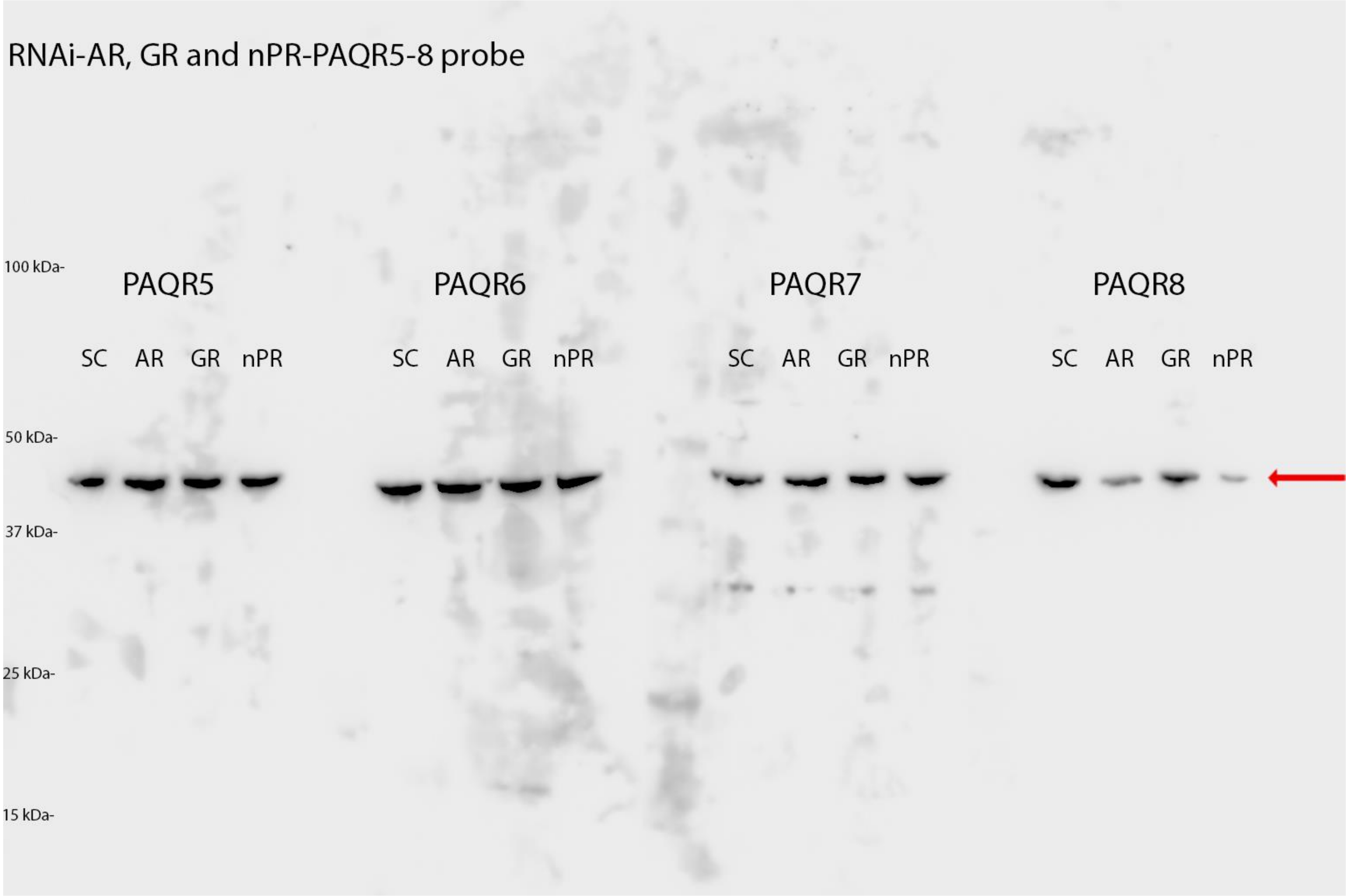
RNAi-AR, GR and nPR-PAQR5-8 probe
100 kDa-
50 kDa-
37 kDa-
25 kDa-
15 kDa-
PAQR5
PAQR6
PAQR7
PAQR8
SC AR GR nPR
SC AR GR nPR
SC AR GR nPR
SC AR GR nPR

Suppl. 3A-2

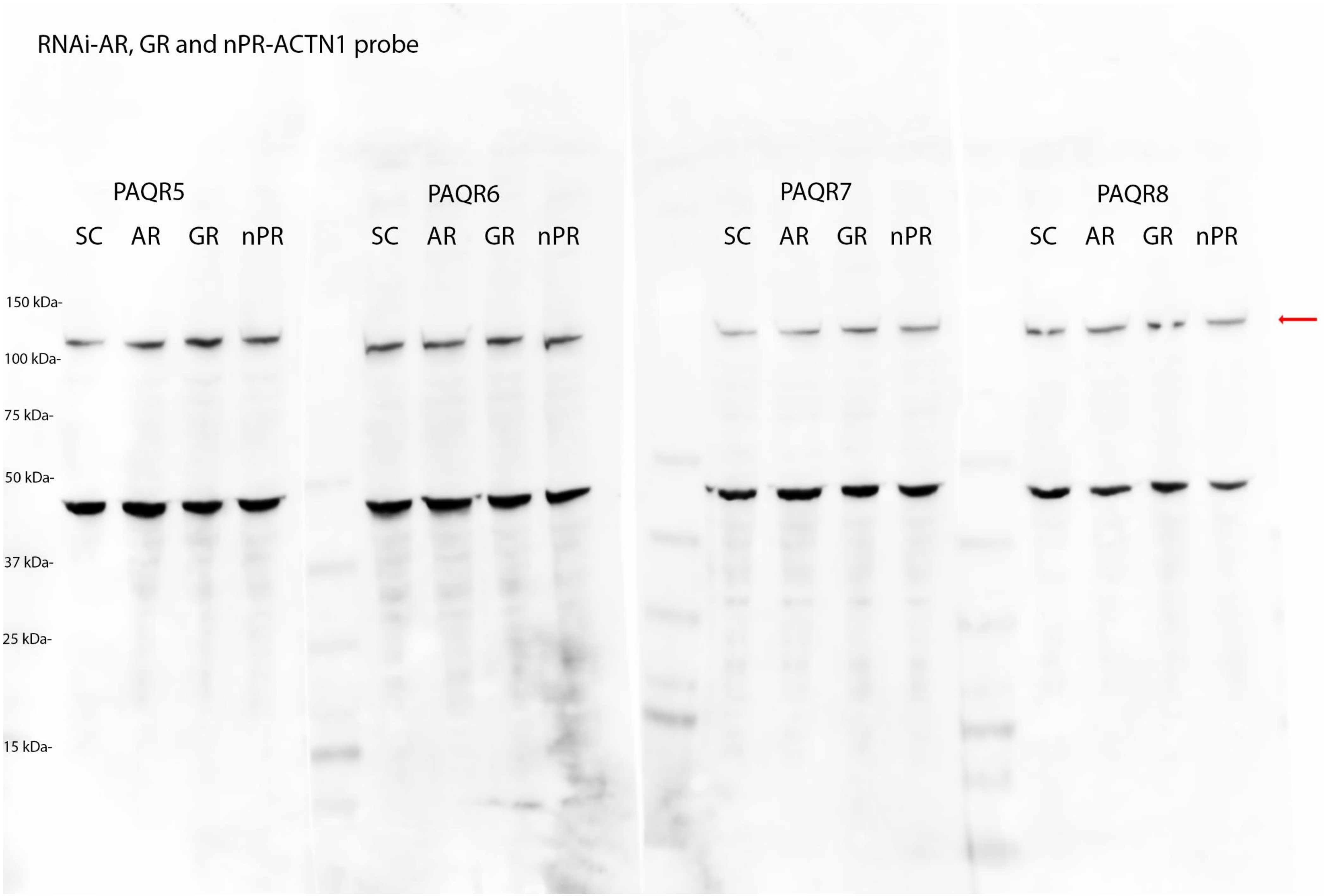
RNAi-AR, GR and nPR-ACTN1 probe
PAQR5
PAQR6
PAQR7
PAQR8
SC AR GR nPR
SC AR GR nPR
SC AR GR nPR
SC AR GR nPR
150 kDa-
100 kDa-
75 kDa-
50 kDa-
37 kDa-
25 kDa-
15 kDa-

Suppl. 3A-3

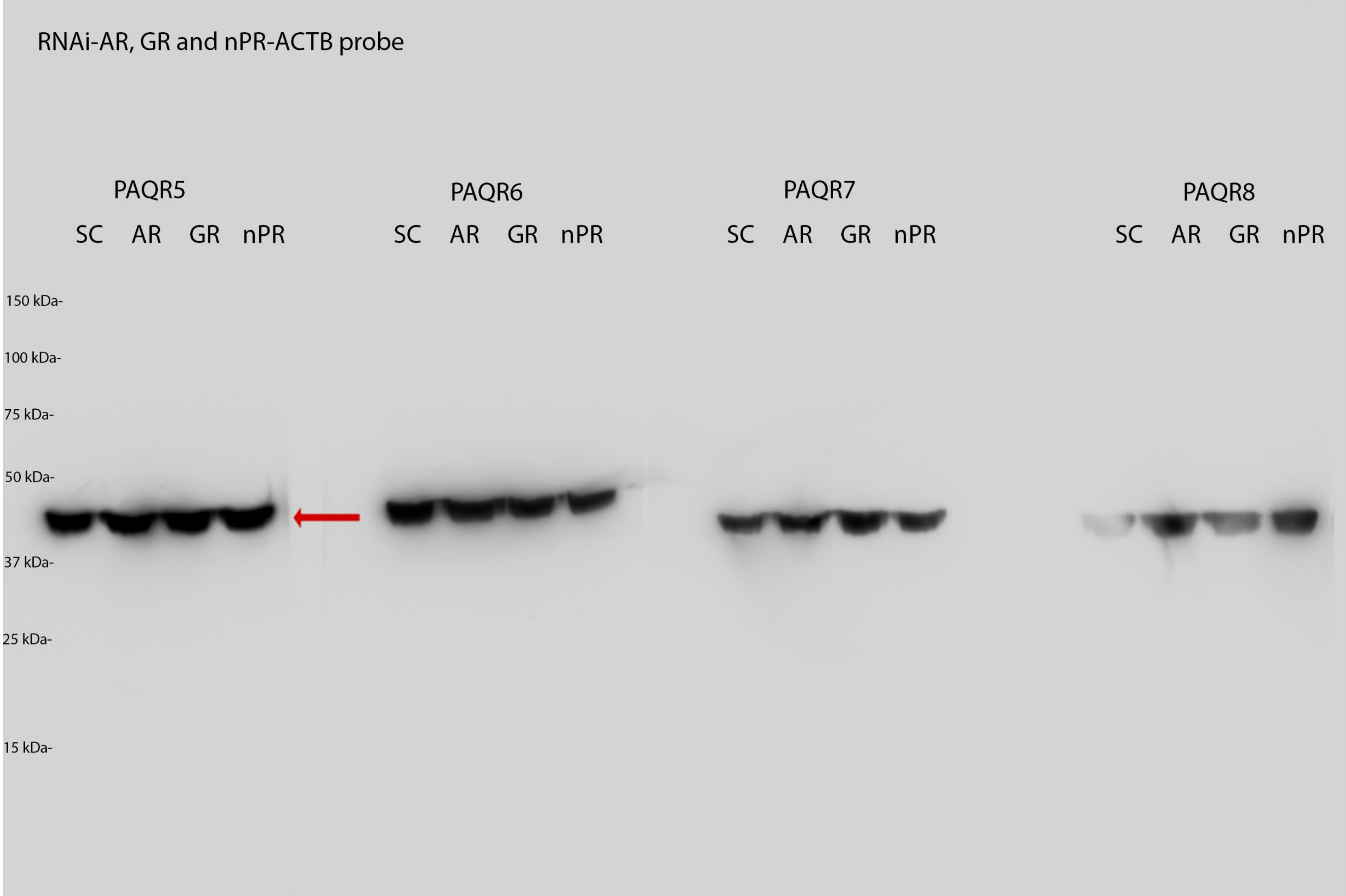

RNAi-AR, GR and nPR-ACTB probe
PAQR5
PAQR6
PAQR7
PAQR8
SC AR GR nPR
SC AR GR nPR
SC AR GR nPR
SC AR GR nPR
150 kDa-
100 kDa-
75 kDa-
50 kDa-
37 kDa-
25 kDa-
15 kDa-

Suppl. 3A-4

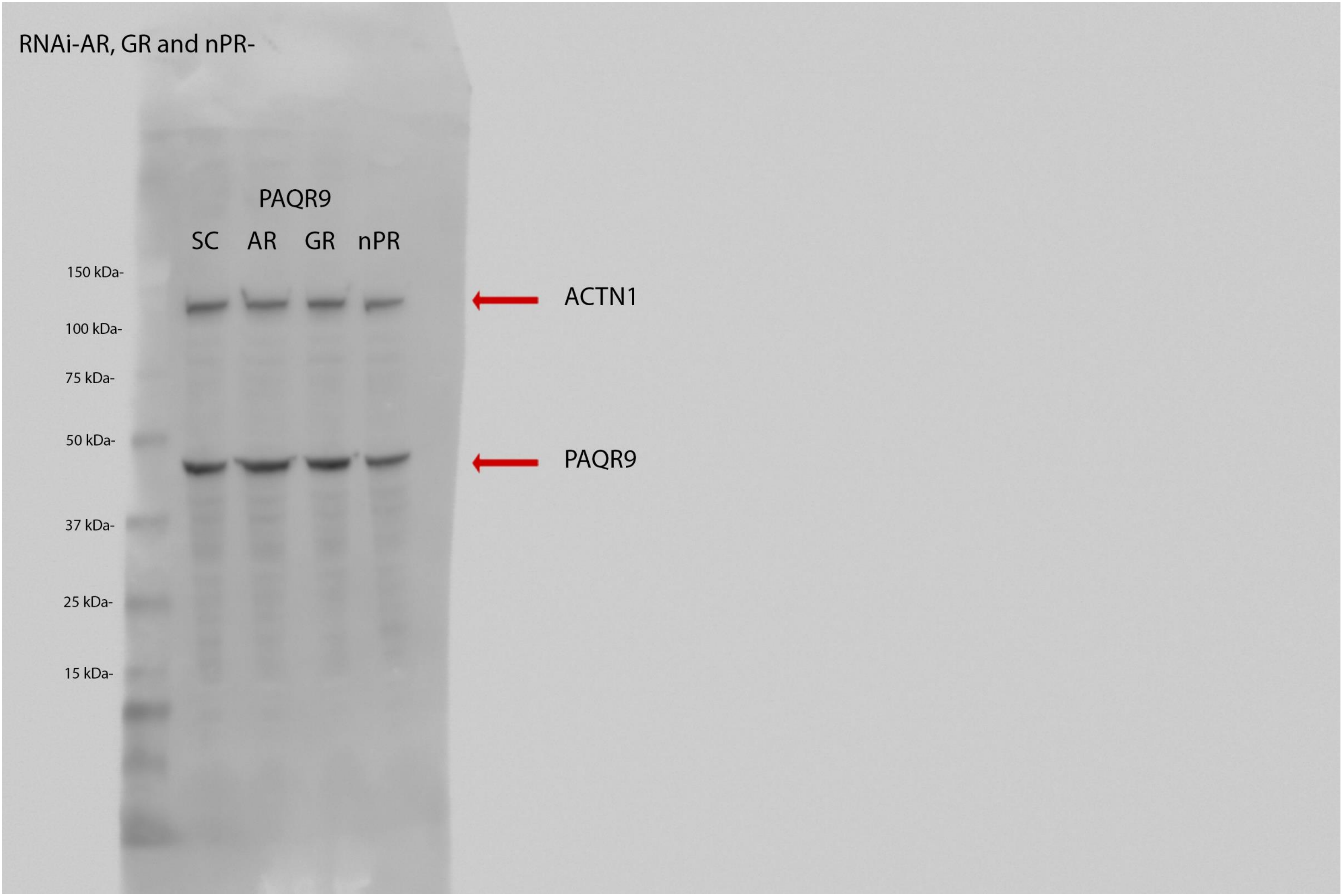

RNAi-AR, GR and nPR-
PAQR9
SC
AR
GR
nPR
150 kDa-
100 kDa-
75 kDa-
50 kDa-
37 kDa-
25 kDa-
15 kDa-
ACTN1
PAQR9

Suppl. 3A-5

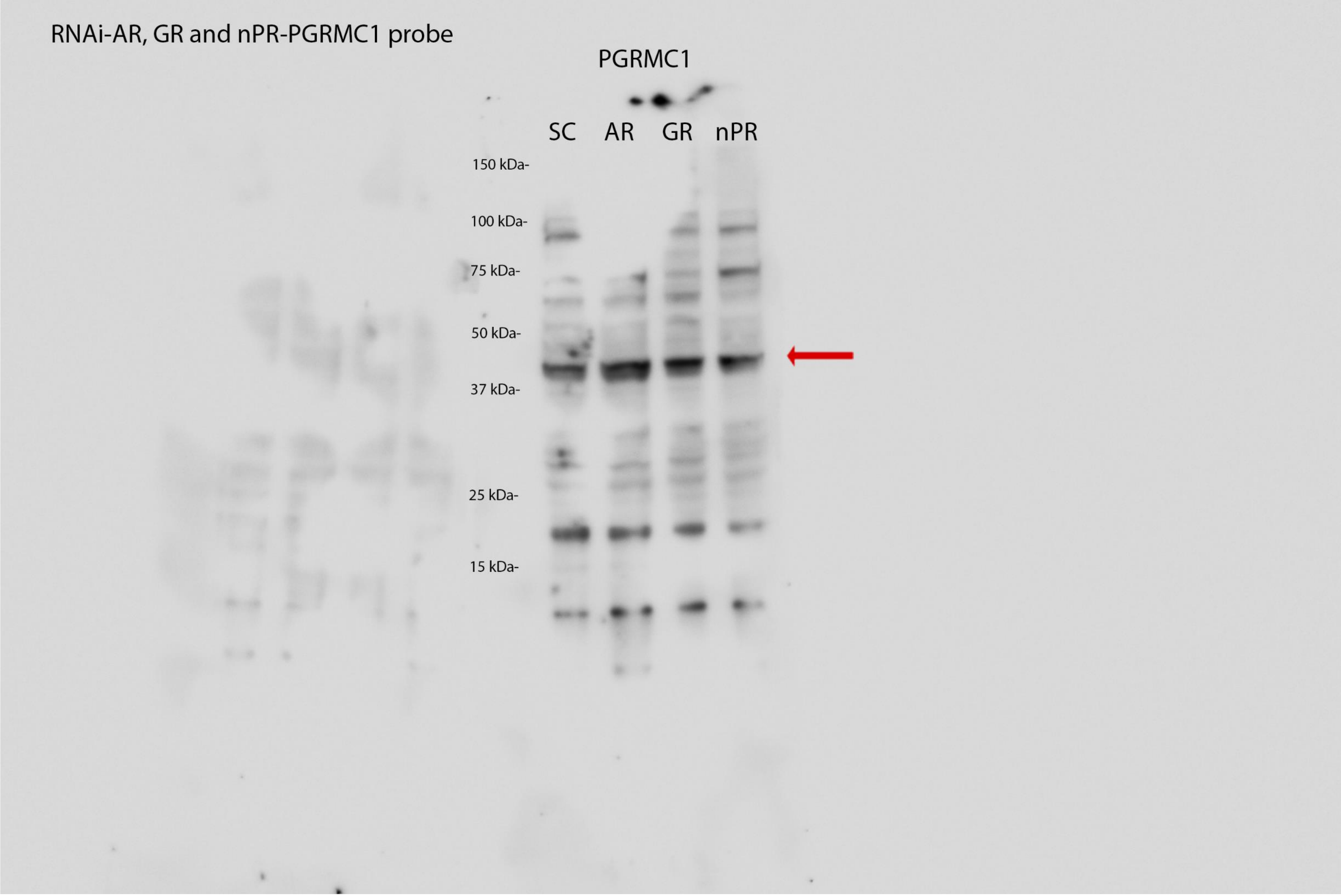
RNAi-AR, GR and nPR-PGRMC1 probe
PGRMC1
SC
AR
GR
nPR
150 kDa-
100 kDa-
75 kDa-
50 kDa-
37 kDa-
25 kDa-
15 kDa-

Suppl. 3A-6

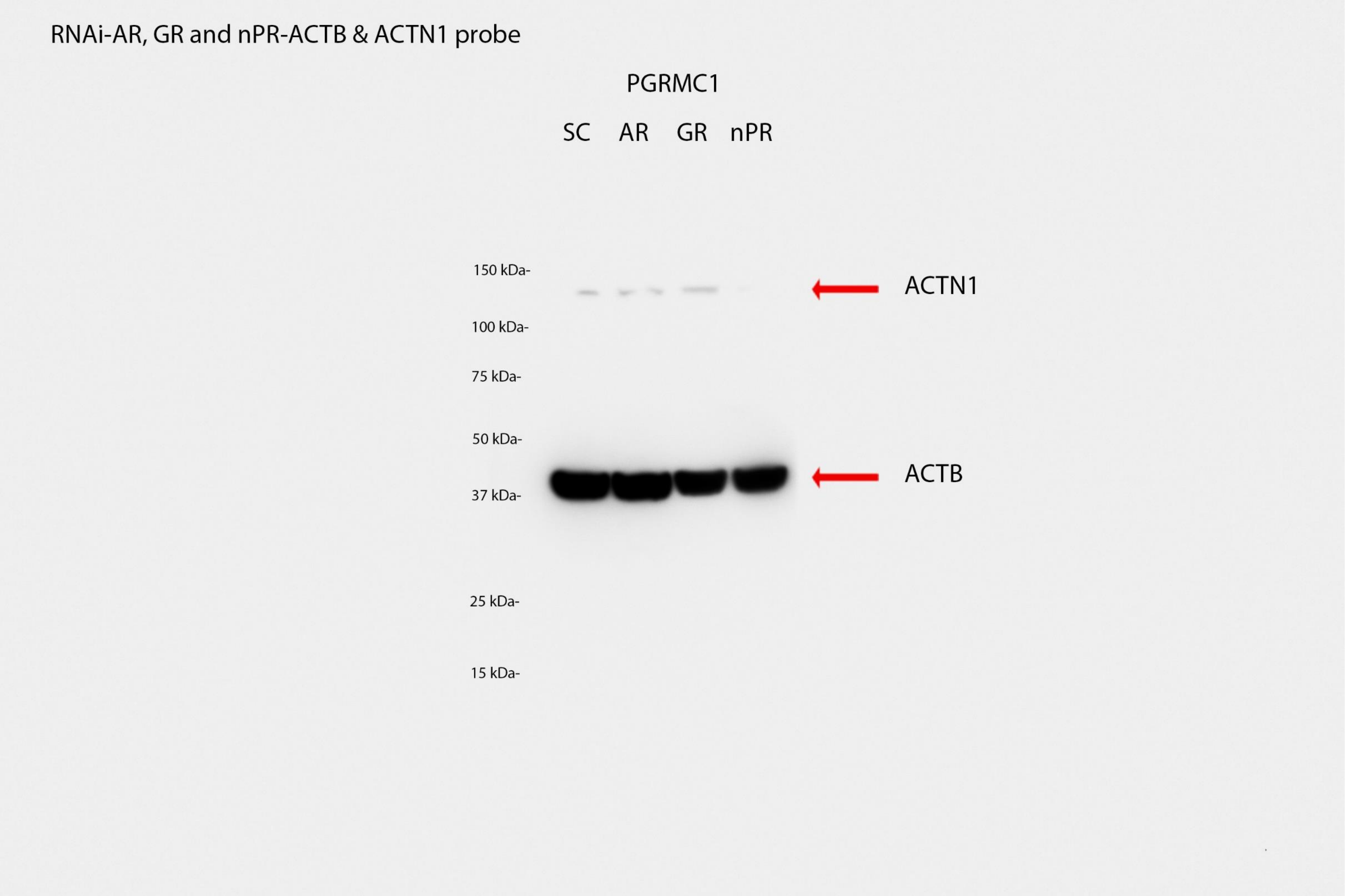
RNAi-AR, GR and nPR-ACTB & ACTN1 probe
PGRMC1
SC
AR
GR
nPR
150 kDa-
100 kDa-
75 kDa-
50 kDa-
37 kDa-
25 kDa-
15 kDa-
ACTN1
ACTB

Suppl. 4A-1

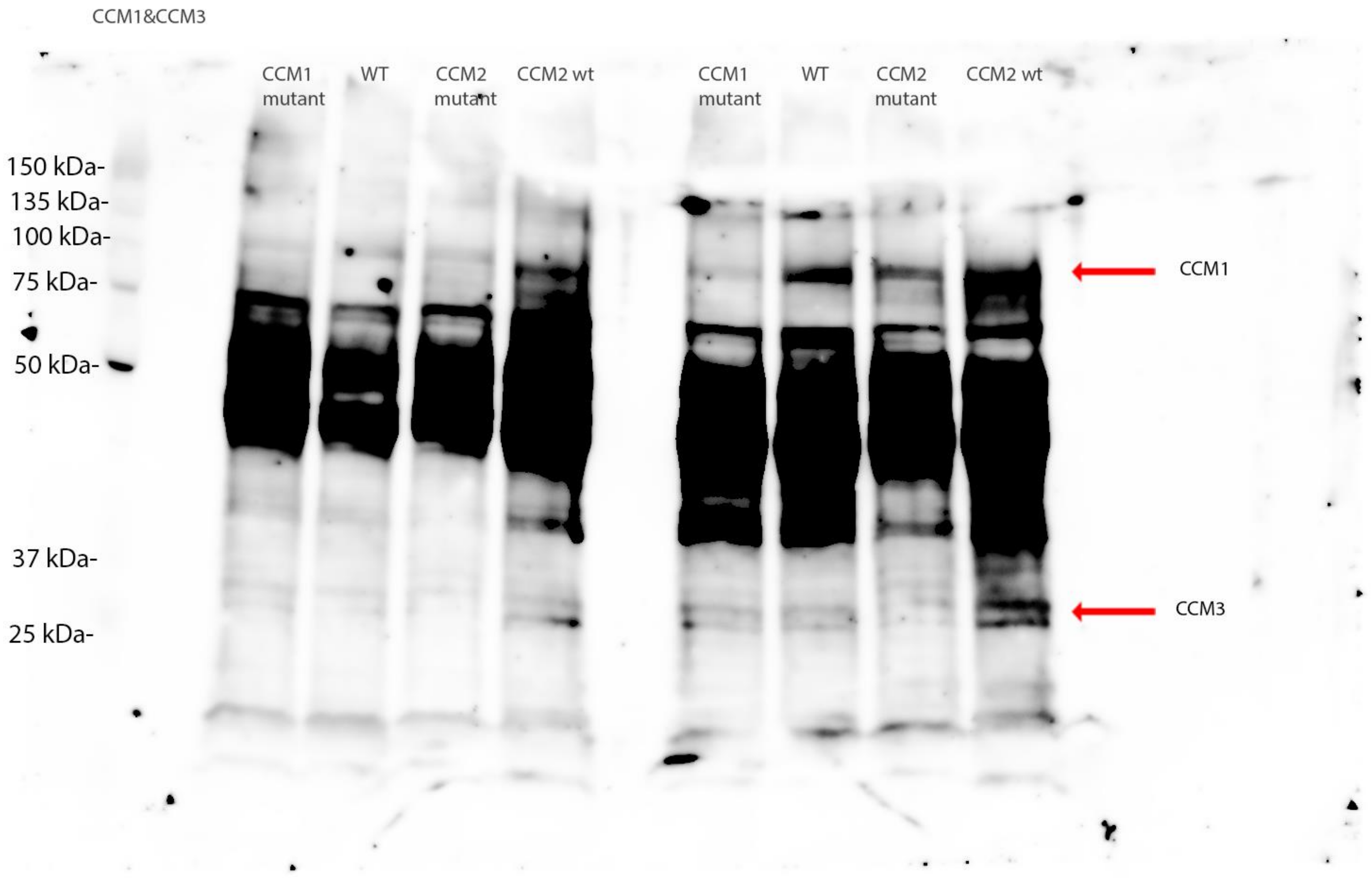
CCM1&CCM3
CCM1 mutant
WT
CCM2 mutant
CCM2 wt
CCM1 mutant
WT
CCM2 mutant
CCM2 wt
150 kDa-
135 kDa-
100 kDa-
75 kDa-
50 kDa-
37 kDa-
25 kDa-
CCM1
CCM3

Suppl. 4A-2

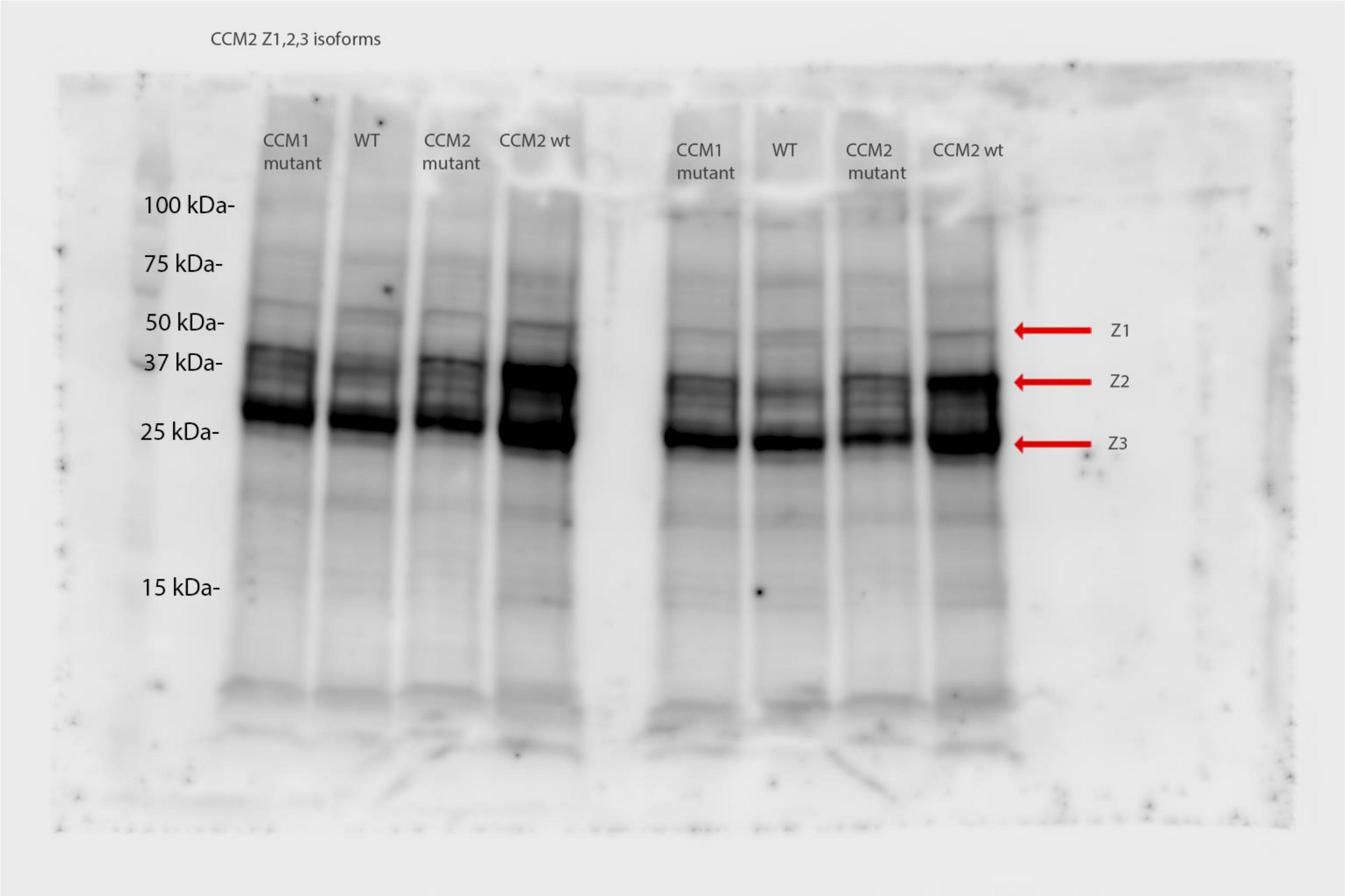

CCM2 Z1,2,3 isoforms
CCM1 mutant
WT
CCM2 mutant
CCM2 wt
CCM1 mutant
WT
CCM2 mutant
CCM2 wt
100 kDa-
75 kDa-
50 kDa-
37 kDa-
25 kDa-
15 kDa-
Z1
Z2
Z3

Suppl. 4A-3

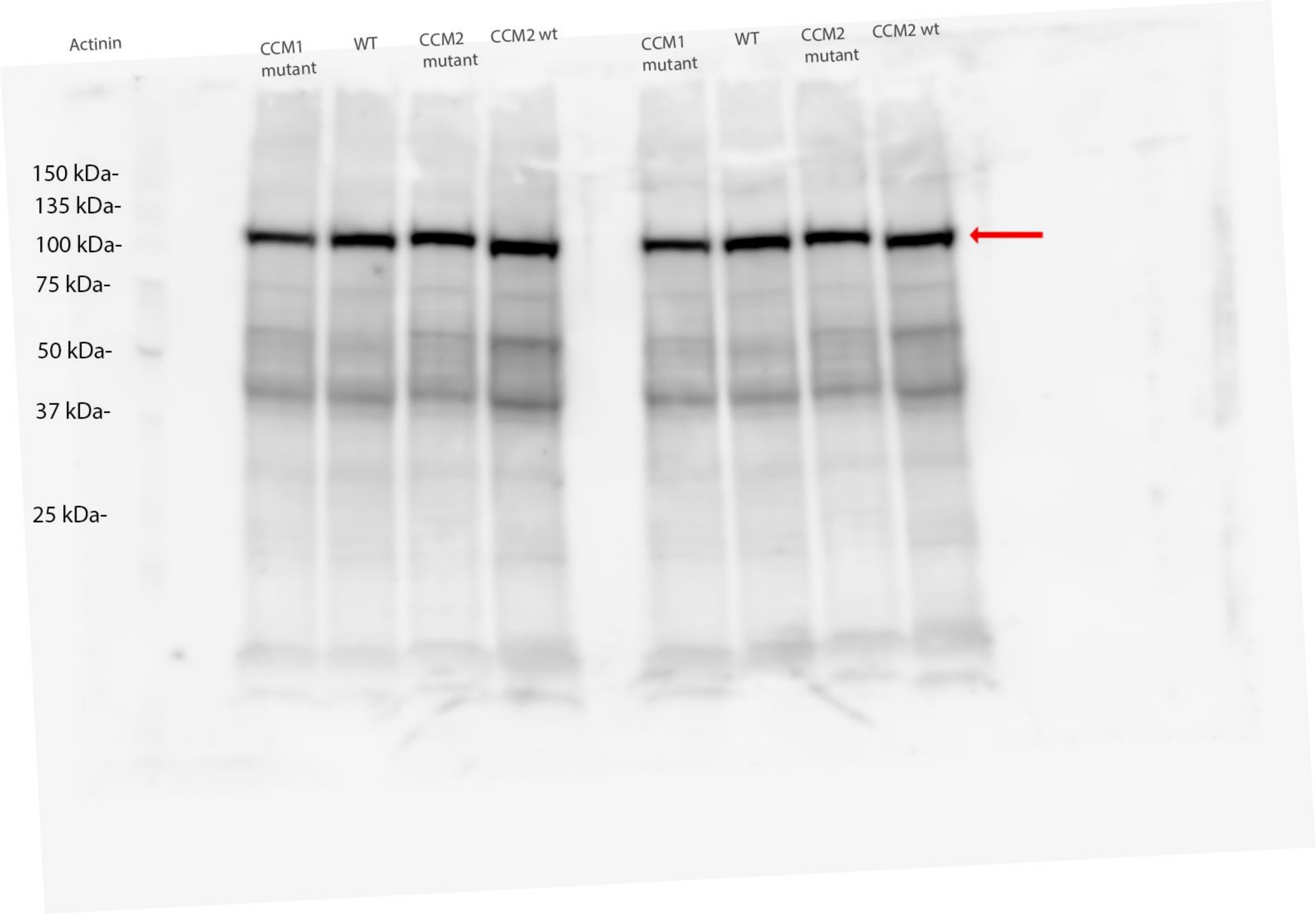

Actinin
CCM1 mutant
WT
CCM2 mutant
CCM2 wt
CCM1 mutant
WT
CCM2 mutant
CCM2 wt
150 kDa-
135 kDa-
100 kDa-
75 kDa-
50 kDa-
37 kDa-
25 kDa-
